# Total Synthesis of Calyciphylline F

**DOI:** 10.1002/anie.202517671

**Published:** 2025-09-15

**Authors:** Ryota Sato, Ryuichi Sumida, Masaki Inoue, Ryota Kotaka, Sangita Karanjit, Kosuke Namba

**Affiliations:** ^1^ Department of Chemistry Graduate School of Science The University of Osaka 1‐1 Machikaneyama Toyonaka Osaka 560‐0043 Japan; ^2^ Graduate School of Pharmaceutical Sciences Tokushima University 1‐78‐1 Shomachi Tokushima 770‐8505 Japan

**Keywords:** Alkaloids, Cycloaddition, Natural products, Radicals, Total synthesis

## Abstract

Calyciphylline F represents the final challenge in the total synthesis of the caged polycyclic‐type of *Daphniphyllum* alkaloids due to the strained 8‐azatricyclo[4.2.1.0^4,8^]nonane ring system. Here, we report the total synthesis of calyciphylline F. We construct the ring system by applying [4 + 3] cycloaddition reaction of pyrroles with 2‐oxyallyl cations to an intramolecular reaction, followed by an intramolecular aldol reaction and capture of the resulting alkoxide as the xanthate. The bridgehead quaternary carbon center is constructed by the intramolecular addition reaction of the bridgehead radical to the alkoxy‐acrylate, which is designed based on the proposed mechanism of by‐product formation. Finally, another 6‐*exo*‐radical cyclization reaction constructs the last remaining ring and achieves the total synthesis of calyciphylline F.

## Graphical abstract

The final challenge in the total synthesis of the caged polycyclic *Daphniphyllum* alkaloids was the Calyciphylline D‐type alkaloid, featuring a strained 8‐azatricyclo[4.2.1.0^4,8^]nonane ring system. Presented herein is the total synthesis of calyciphylline F, a member of the calyciphylline D‐type alkaloid family, by an intramolecular [4 + 3] cycloaddition reaction of pyrroles, an intramolecular aldol reaction, and three C─C bond‐forming radical reactions.

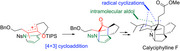

## Introduction

**Figure 1:**
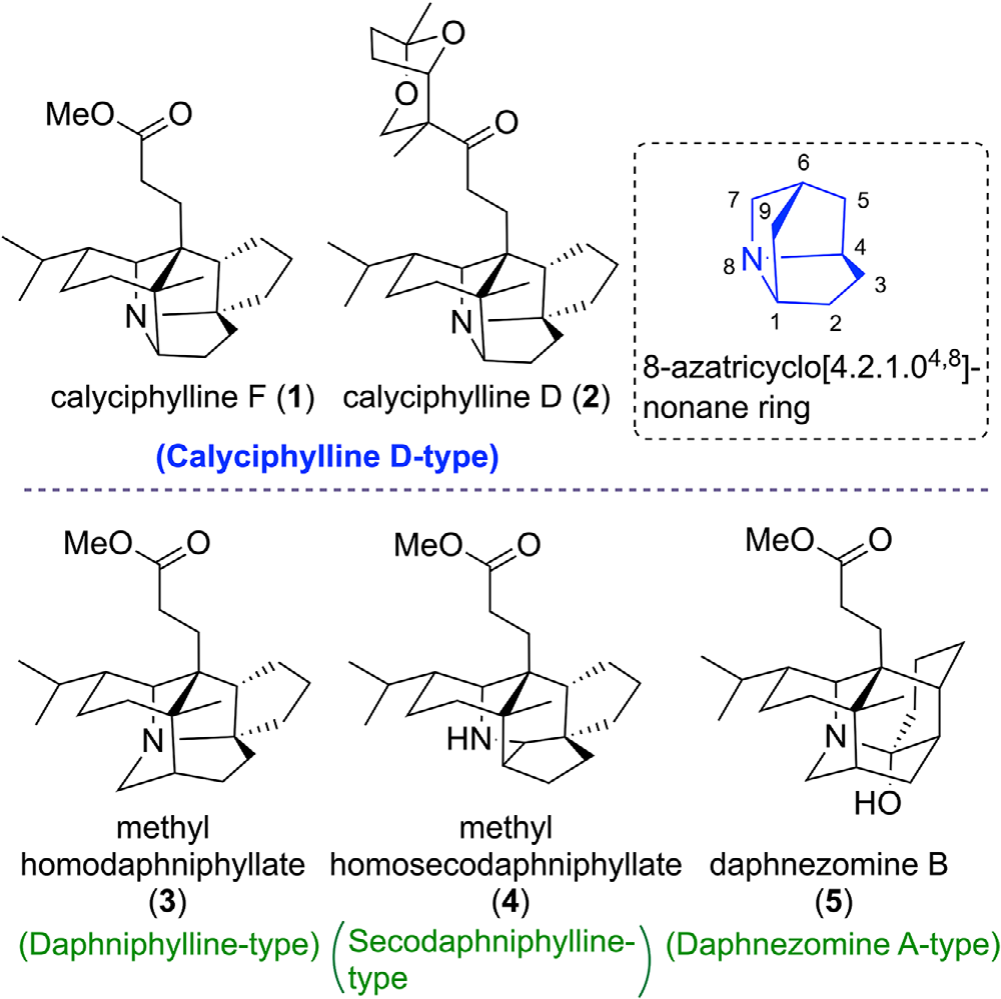
Structures of caged polycyclic *Daphniphyllum* alkaloids. Calyciphylline F (**1**) and calyciphylline D (**2**) contain strained 8‐azatricyclo[4.2.1.0^4,8^]nonane ring core in their complex pentacyclic ring system.

The *Daphniphyllum* alkaloids comprise a large family of natural products and have received a great deal of attention for their potent and various biological activities and complex molecular architectures.^[^ ^1^, ^2^ ^]^ In particular, the complex chemical structure of *Daphniphyllum* alkaloids has attracted the interest of many synthetic chemists.^[^ ^3^, ^4^ ^]^ In recent years, the total synthesis of complex *Daphniphyllum* alkaloids has been achieved one after another,^[^ ^5^, ^6^, ^7^, ^8^, ^9^, ^10^, ^11^, ^12^, ^13^, ^14^, ^15^, ^16^, ^17^, ^18^, ^19^, ^20^, ^21^, ^22^, ^23^, ^24^, ^25^, ^26^, ^27^, ^28^, ^29^, ^30^, ^31^, ^32^ ^]^ and *Daphniphyllum* alkaloids are currently one of the most remarkable natural product families in the field of natural product synthesis. Calyciphylline F (**1**) was isolated as a member of the caged polycyclic *Daphniphyllum* alkaloids from the plant *Daphniphyllum calycinum* in 2007 by Kobayashi et al.^[^ ^33^ ^]^ Calyciphylline D (**2**), in which the bridgehead side chain is replaced with a ketone having a bicyclic acetal,^[^ ^34^ ^]^ and caldaphnidine M, in which the methyl ester is converted to a carboxylic acid,^[^ ^35^ ^]^ have also been isolated as related natural products (Figure 1), and **1** is classified as a calyciphylline D‐type alkaloid. Although the biological activity of **1** is unknown, the related **2** has been shown to promote mRNA expression of nerve growth factor and is expected to be developed as a lead compound for the treatment of neurodegenerative diseases.^[^ ^1^ ^]^ The structural features of calyciphylline F include seven contiguous stereogenic centers containing quaternary carbon centers; a polycyclic skeleton with extremely few functional groups; and a complex caged pentacyclic ring system. Other related *Daphniphyllum* alkaloids with a caged polycyclic skeleton include methyl homodaphniphyllate (**3**) as a daphniphylline‐type alkaloid,^[^ ^36^, ^37^, ^38^ ^]^ methyl homosecodaphniphyllate (**4**) as a secodaphniphylline‐type alkaloid,^[^ ^39^ ^]^ and daphnezomine B (**5**) as a daphnezomine A‐type alkaloid^[^ ^40^ ^]^ (Figure 1), and the total syntheses of these other alkaloids have already been achieved.^[^ ^41^, ^42^, ^43^, ^44^, ^45^, ^46^, ^47^, ^48^, ^49^ ^]^ In contrast, the construction of the corresponding pentacyclic ring skeleton of calyciphylline D‐type alkaloids has not yet been achieved, let alone the total synthesis of calyciphylline F. To our knowledge, the only synthetic study of the calyciphylline D‐type alkaloids is the report of Wang et al.^[^ ^50^ ^]^ This is because calyciphylline D‐type alkaloids contain a strained 8‐azatricyclo[4.2.1.0^4,8^]nonane ring skeleton in their complex pentacyclic ring system, which is a feature not found in any other natural product. Indeed, synthesis of the 8‐azatricyclo[4.2.1.0^4,8^]nonane ring has been performed for only a few simple azatricycles,^[^ ^51^, ^52^ ^]^ and functionalized azatricycles or azatricycles with the quaternary carbon center at the bridgehead positions have never been synthesized. There is also a concern that the exposed bridgehead nitrogen will be easily oxidized. Therefore, the complex calyciphylline D‐type alkaloid, which contains the difficult‐to‐synthesize 8‐azatricyclo[4.2.1.0^4,8^]nonane ring skeleton, is the final hurdle in total synthesis of the caged polycyclic *Daphniphyllum* alkaloids. Herein, we report the first total synthesis of calyciphylline F as a calyciphylline D‐type alkaloid, utilizing the [4 + 3] cycloaddition reaction of pyrroles with oxyallyl cations, which was originally developed in our laboratory.

## Results and Discussion

**Figure 2:**
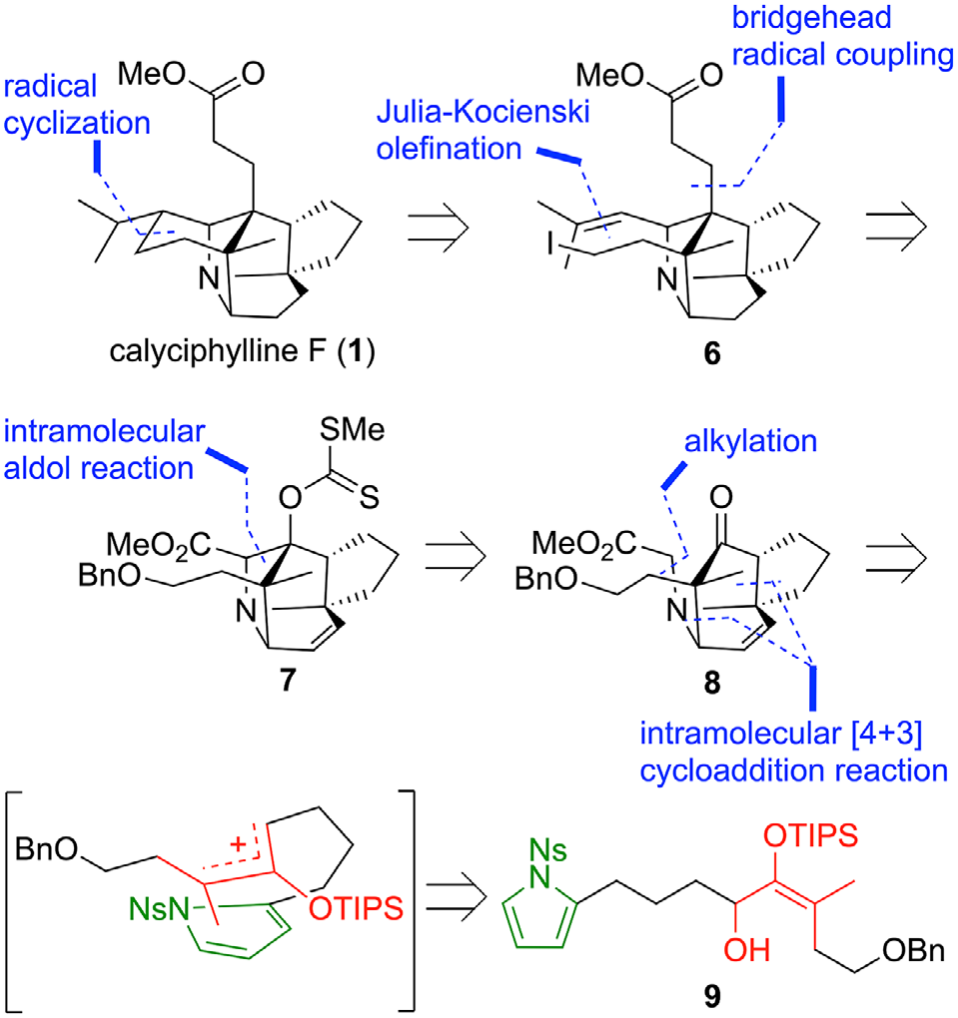
Synthetic plan for calyciphylline F (**1**). Calyciphylline F (**1**) would be synthesized by three carbon─carbon bond formations via radical reaction and an intramolecular [4 + 3] cycloaddition reaction of a pyrrole with an oxyallyl cation.

The retrosynthetic analysis is shown below (Figure 2). Since calyciphylline F (**1**) has almost no functional groups that can serve as a foothold for the formation of carbon─carbon bonds, the cyclohexane ring of **1** would be constructed by a 6‐*exo* radical cyclization reaction of the isopropylidene group of **6**. We considered that the isopropylidene group of **6** could be formed by the Julia–Kocienski reaction^[^ ^53^ ^]^ and that the quaternary carbon center at the bridgehead position could be constructed by the addition reaction of the bridgehead radical derived from xanthate **7**. We reasoned that the tetracyclic ring system of **7** would be constructed by the intramolecular aldol reaction of the tricyclic compound **8**, and that this approach would allow us to synthesize the 8‐azatricyclo[4.2.1.0^4,8^]nonane ring system. We planned to construct the tricyclic ring system of **8** in a single step by the intramolecular [4 + 3] cycloaddition reaction of the pyrrole with the 2‐oxyallyl cation. In earlier studies, we had already developed an intermolecular [4 + 3] cycloaddition reaction of pyrroles with oxyallyl cations as an original method to efficiently construct the tropane skeleton.^[^ ^54^, ^55^ ^]^ Therefore, we planned to apply this reaction to the intramolecular reaction of **9**. There are very few examples of intramolecular [4 + 3] cycloaddition reactions of pyrroles leading to polycyclic tropanes,^[^ ^56^ ^]^ and this is especially true for intramolecular reactions tethered by carbon atoms, for which there has been only one pioneering example, reported by Chiu in 2018.^[^ ^57^ ^]^

**Scheme 1:**
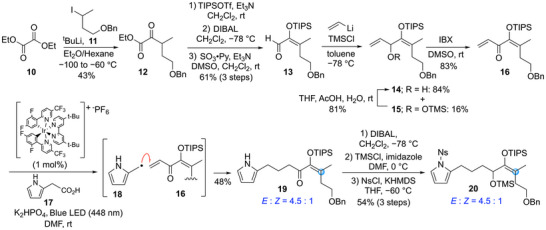
Synthesis of precursor **20** for intramolecular [4 + 3] cycloaddition reaction.

First, the precursor **9** for the intramolecular [4 + 3] cycloaddition reaction was synthesized. Starting with diethyl oxalate **10**, the addition of the carbanion derived from the halogen–lithium exchange reaction of the iodide **11** afforded the keto ester **12**. After treatment of **12** with triisopropylsilyl trifluoromethanesulfonate (TIPSOTf) and triethylamine to form silyl enol ether, the ethyl ester of **12** was reduced with diisobutylaluminium hydride (DIBAL) followed by Parikh–Doering oxidation to afford aldehyde **13**. Only the *E*‐form was obtained in the silyl enol ether formation reaction, probably due to the steric hindrance of TIPS group. Although the addition reaction of vinyl Grignard reagent to **13** did not proceed, the desired allyl alcohol **14** was obtained by treating **13** with vinyl lithium in the presence of trimethylsilyl chloride (TMSCl) in toluene. Since a small amount of TMS ether **15** was also obtained, it was converted to allyl alcohol **14** by removal of the TMS group. Allyl alcohol **14** was converted to enone **16** by oxidation with 2‐iodoxybenzoic acid (IBX). We then applied the photoredox reaction conditions developed by MacMillan et al.^[^ ^58^ ^]^ to the reaction of pyrrolecarboxylic acid **17** ^[^ ^59^ ^]^ with enone **16**, in which the pyrrolemethyl radical **18** generated from **17** is added to enone **16**, and the desired adduct **19** was obtained in modest yield. During this process, the isomerization of the silyl enol ether partially occurred, resulting in an *E*/*Z* ratio of 4.5/1. As the longer reaction time induced further isomerization, the reaction was conducted in small batches over a short period of time, about 20 min. Incidentally, although we tried several methods to assemble the pyrrole unit and the silyloxyenone unit, including aldol reactions, alkylation reactions, and olefin metathesis, the photoredox reaction provided the coupling product most efficiently (Scheme S1). The coupling product **19** was converted into the precursor **20** for the intramolecular [4 + 3] cycloaddition reaction by sequential reactions of DIBAL reduction of the ketone group, protection of the resulting secondary alcohol with the trimethylsilyl group, and introduction of a nosyl group to the pyrrole nitrogen. Since the [4 + 3] cycloaddition reaction developed in our laboratory permits the generation of oxyallyl cations not only from free allyl alcohols but also from allyl ethers, we performed the cycloaddition reaction without removal of the TMS group (Scheme 1).

**Scheme 2:**
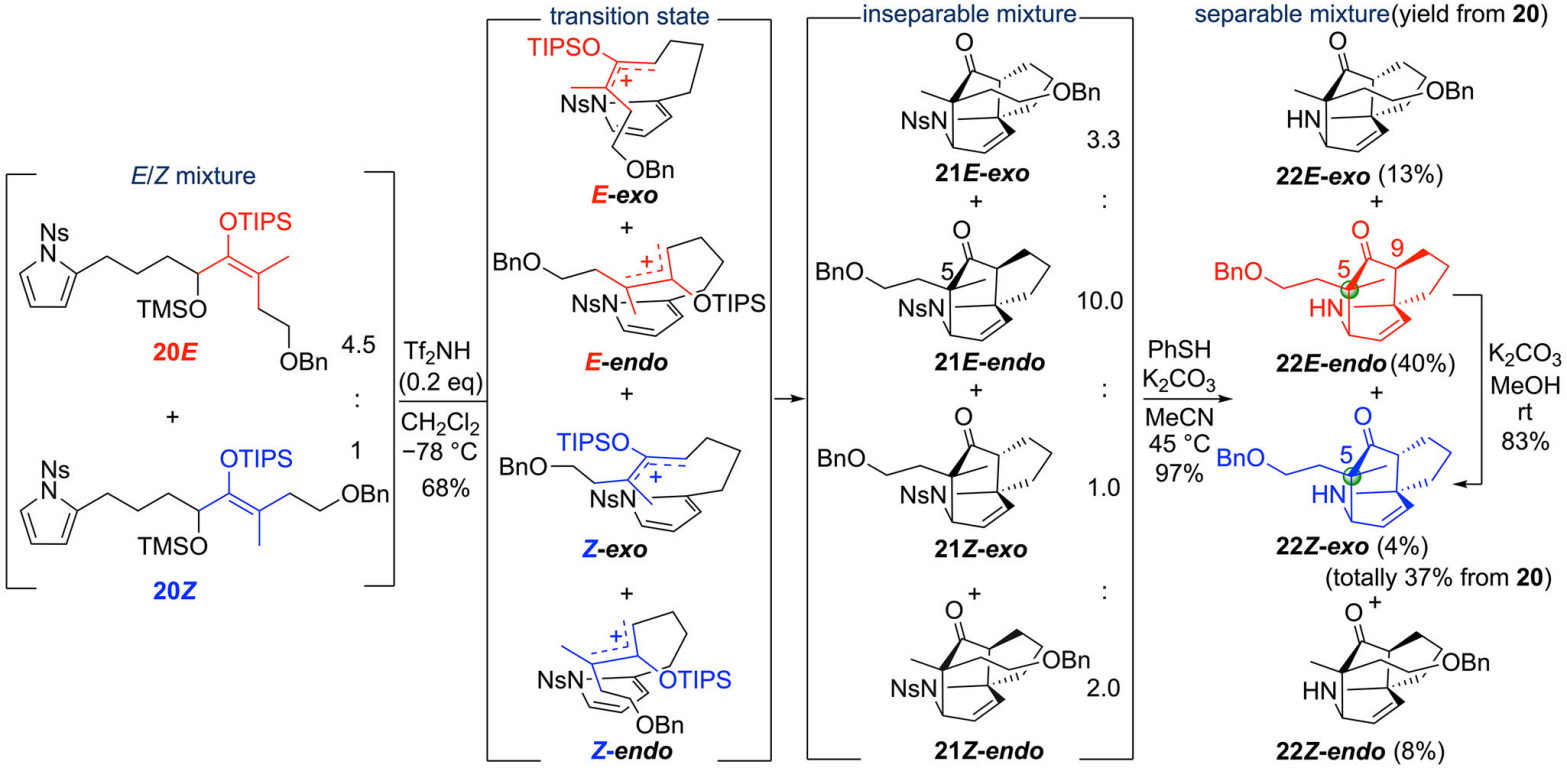
Intramolecular [4 + 3] cycloaddition reaction of **20**.

Having prepared precursor **20**, we next examined the intramolecular [4 + 3] cycloaddition reaction. After investigating various factors such as acid, solvent, and temperature (Table S1), we found that treatment of **20** with 0.2 equiv of Tf_2_NH in dichloromethane at −78 °C allowed the reaction to proceed smoothly, yielding the cycloadduct **21** in 68% yield as an inseparable diastereomeric mixture. Verification of the diastereomeric ratio showed that the ratio of **21*E*‐*exo***:**21*E***‐***endo***:**21*Z*‐*exo***:**21*Z***‐***endo*** was 3.3:10:1.0:2.0. After separation and purification of **20*E*** and **20*Z***, each reaction of **20*E*** and **20*Z*** under similar conditions afforded the 3:1 mixture of **21*E*‐*endo*** and **21*E*‐*exo*** and the 2:1 mixture of **21*Z*‐*endo*** and **21*Z*‐*exo***, respectively (Scheme S2). This result indicated that the ratio of *endo*:*exo* in the transition state of the cyclization reaction was 3:1 for the *E*‐form and 2:1 for the *Z*‐form. Thus, the conversion of **20**, consisting of the 4.5:1 mixture of *E*‐ and *Z*‐forms, proceeded with *endo*:*exo* ratios of 10:3.3 (ca 3:1) and 2:1, respectively, resulting in the above ratios. Specifically, the ratio of the sum of **21*E*‐*endo*** and **21*E*‐exo** (10 + 3.3), derived from **20*E***, to the sum of **21*Z*‐*endo*** and **21*Z*‐*exo*** (2 + 1), derived from **20*Z***, is 13.3:3, which is approximately 4.5:1. Unlike intermolecular cycloadditions, which proceed through a stepwise mechanism with high endo‐selectivity,^[^ ^54^, ^55^ ^]^ intramolecular reactions did not give the similar endo‐selectivity, probably due to the steric strain of endo‐products. There was no difference in the reaction rate between the *E*‐ and *Z*‐forms, although the yield was slightly higher for *E*‐form. Since it was difficult to further improve the endo selectivity, we drove the total synthesis forward from the diastereomeric mixture with this ratio.^[^ ^60^ ^]^ The removal of the nosyl group of **21** proceeded smoothly, and a diastereomeric mixture of amine **22** was obtained in 97% yield. At this stage, since it became possible to separate the diastereomers, we isolated **22*E*‐*endo*** and **22*Z*‐*exo***, which have the same stereochemistry at the quaternary carbon center at the C‐5 position as the natural product. Subsequent isomerization of **22*E*‐*endo*** at the C‐9 position converged to **22*Z*‐*exo***. Therefore, this intramolecular [4 + 3] cycloaddition reaction approach enabled the single‐step construction of the complex tricyclic skeleton of calyciphylline F and the quaternary carbon center at the C‐5 position (Scheme 2).

**Scheme 3:**
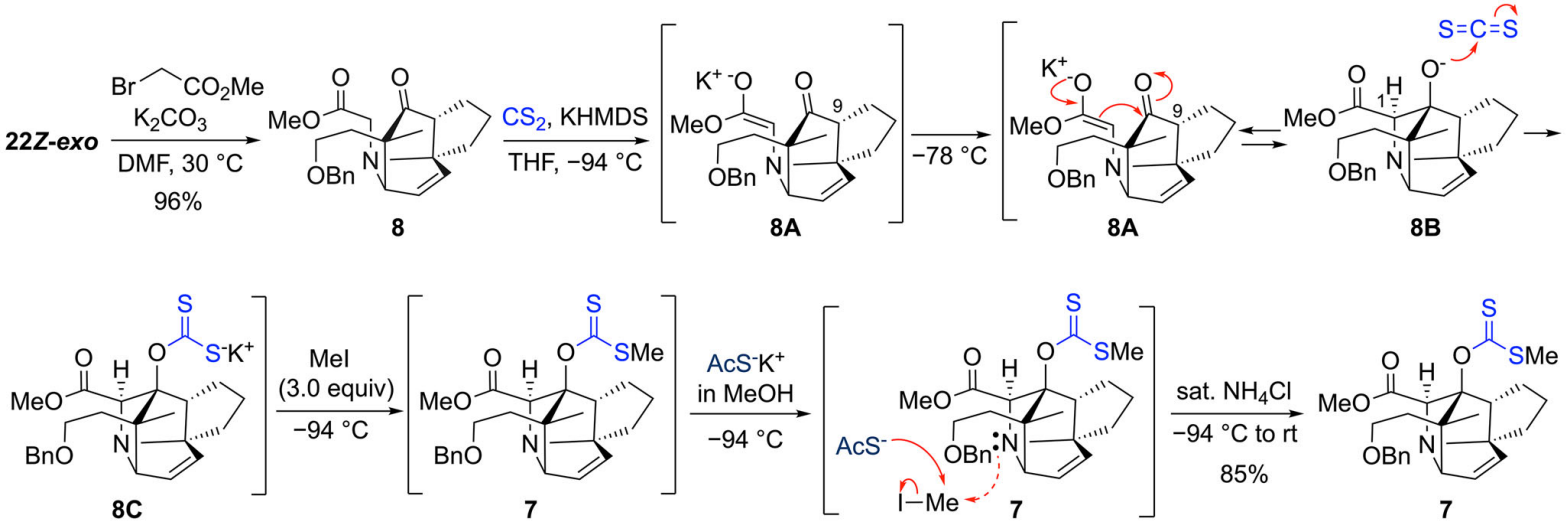
Construction of the strained 8‐azatricyclo[4.2.1.0^4,8^]nonane ring core.

Having established the synthesis of the tricyclic ring core, we next set out to construct the 8‐azatricyclo[4.2.1.0^4,8^]nonane ring skeleton. Since the ketone groups of **21** and **22** were sterically shielded and did not react with any reducing agents or nucleophiles, the intramolecular nucleophilic addition reaction was applied. The methoxycarbonylmethyl group was introduced into the amine nitrogen of **22*Z*‐*exo*** by alkylation to form **8**, and the cyclization was attempted by an intramolecular aldol‐type reaction from the methyl ester moiety to the ketone of **8**. However, even when **8** was treated with various strong bases such as LDA and KHMDS, none of the obtained cyclization products underwent the desired intramolecular aldol reaction. Because this was attributed to the 8‐azatricyclo[4.2.1.0^4,8^]nonane ring of the aldol adduct being strained, favoring the retroaldol reaction, we next attempted to capture the alkoxide generated by the addition reaction. Considering the construction of a bridgehead quaternary carbon center by radical reactions, the capture as a xanthate was investigated. After various examinations, the following conditions were established to give the desired tetracyclic compound **7** in high yield. A mixture of **8** and CS_2_ was treated with KHMDS at −94 °C to selectively generate the kinetically favorable ester enolate **8A** rather than the enolate at the C9 position, and then the mixture was warmed to −78 °C to allow the intramolecular aldol reaction to proceed. The aldol adduct **8B** and the ester enolate **8A** were in equilibrium, and the alkoxide of **8B** was captured by CS_2_ and converged from the equilibrium to potassium xanthate **8C**. Then, after cooling the reaction mixture again to −94 °C, 3.0 equiv of methyl iodide was added and the alkylation proceeded to form xanthate **7**. The lower temperature of −94 °C and 3.0 equiv of methyl iodide were required, respectively, to avoid the methylation of the bridgehead nitrogen and to complete the reaction. Addition of potassium thioacetate to decompose the remaining methyl iodide followed by quenching the reaction with sat. NH_4_Cl aqueous solution afforded the xanthate **7**, which contained the desired 8‐azatricyclo[4.2.1.0^4,8^]nonane ring skeleton, in 85% yield (Scheme 3). The stereochemistry at the C1 position was probably constructed to avoid steric repulsion with the rigid five‐membered ring. The supporting information explains the details of the complex operation in this intramolecular aldol reaction, including examinations of various nucleophiles that decompose methyl iodide (Table S2).

**Scheme 4:**
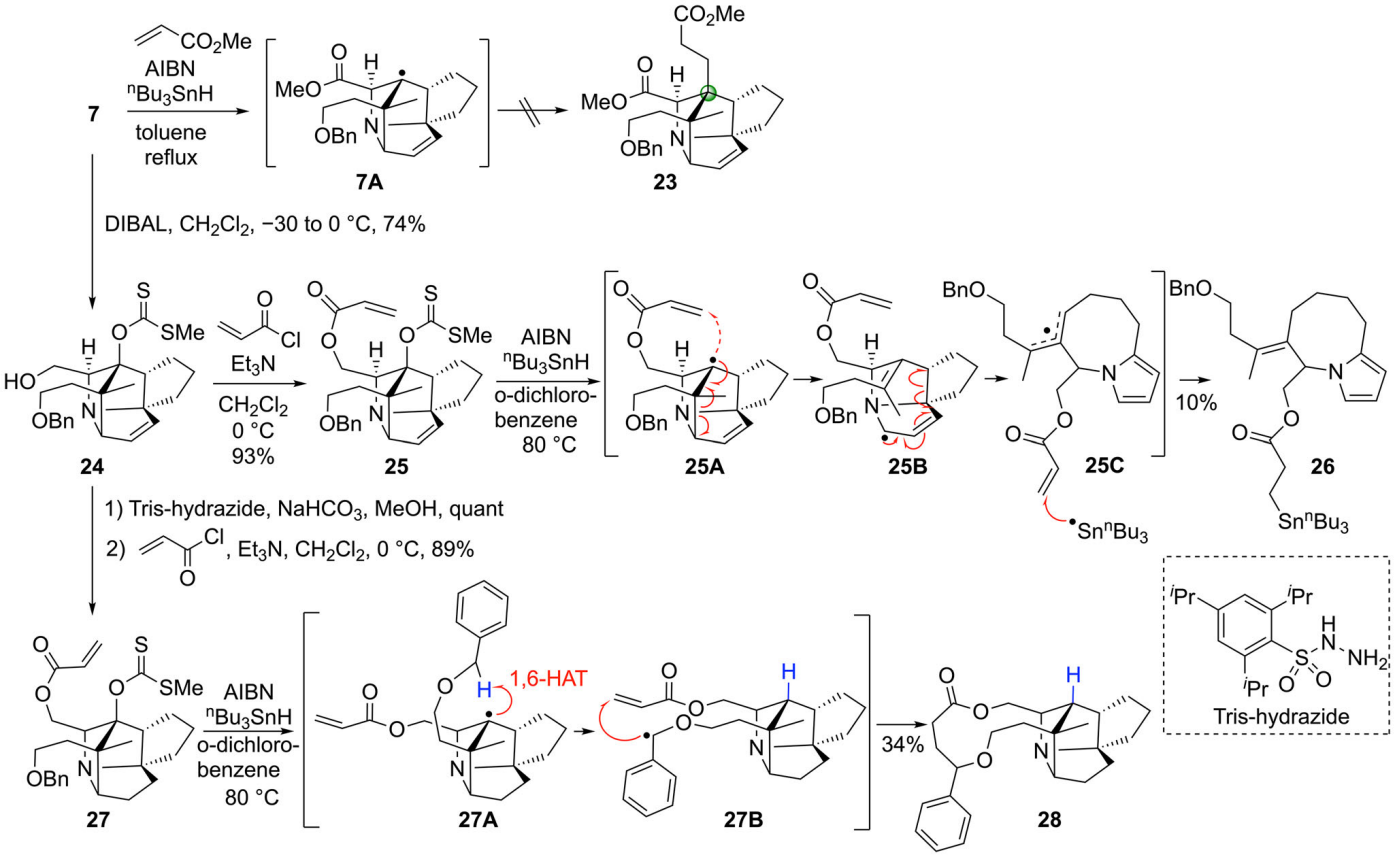
Investigation of the introduction of the bridgehead side chain.

Having constructed the strained 8‐azatricyclo[4.2.1.0^4,8^]nonane ring core, we next investigated the introduction of a side chain at the bridgehead position by the radical addition reaction (Scheme 4). Numerous conditions, such as using various radical acceptors, including methyl acrylate, or changes to the radical initiators, reducing agents, solvents, temperature, and application of microwave‐accelerated method developed in our laboratory,^[^ ^61^ ^]^ were attempted, but either a reduced product was obtained at the bridgehead position, or the starting material was decomposed. This indicated that although the bridgehead radical was formed, the reaction with the reducing agents or decomposition proceeded preferentially rather than the addition to radical acceptors. To solve this problem, intramolecular placement of a radical acceptor was attempted. The methyl ester of **7** was reduced to the alcohol **24**, and subsequent condensation with acryloyl chloride afforded **25** with the acrylic ester as the radical acceptor. With the precursor **25** in hand, intramolecular radical reaction was attempted under various conditions (Table S3). However, the desired cyclization product was not obtained under any of the conditions and the major product was a tropane ring cleavage product **26**. It was assumed that **26** was obtained by four sequential steps: generation of the bridgehead radical **25A** from **25**, cleavage of **25A** to form the allyl radical **25B**, further cleavage of **25B** to give **25C**, and hydride reduction of **25C**. Since the formation of the stable allyl radical **25B** was considered to be the driving force behind the cleavage reaction of **25A**, we attempted to reduce the double bond of the pyrroline ring before the radical reaction. The double bond of alcohol **24** was reduced by tris‐hydrazide, and acrylic acid was similarly introduced to lead to **27**. However, although the similar radical cyclization reactions using **27** were performed under various conditions, the desired cyclization product was not obtained at all, even with this substrate. The major product of this reaction was a macrocyclic lactone **28**, and we proposed the following reaction mechanism. After formation of the bridgehead radical **27A**, there was a 1,6‐shift of the hydrogen at the benzylic position, and the resulting benzyl radical of **27B** was added to the acrylate ester moiety, leading to **28**. In addition, when the protecting group was changed from a benzyl group to a TBS group in order to suppress the 1,6‐HAT, only reduction at the bridgehead position proceeded, and the desired cyclization product was not obtained (Scheme 4).

**Figure 3:**
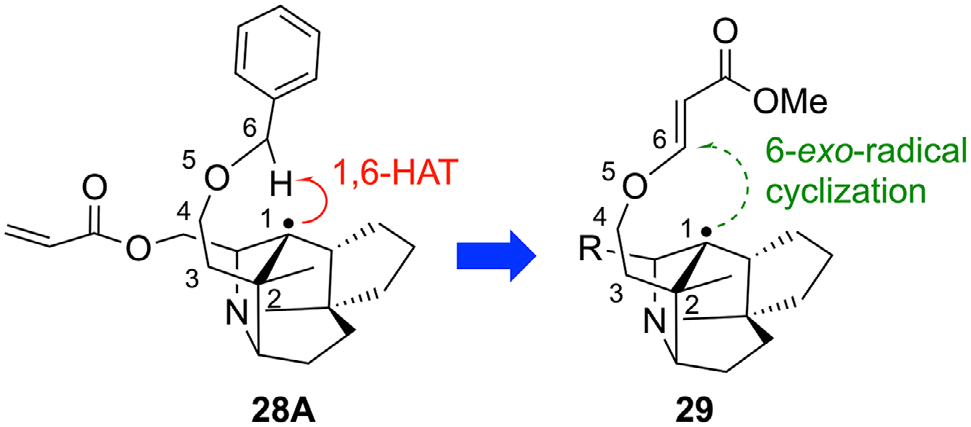
Redesign of the cyclization precursor.

Although the above study did not allow us to construct the bridgehead quaternary carbon center, we focused on the 1,6‐HAT that occurred in the cyclization reaction of **27**. The progression of this 1,6‐HAT suggests that the hydrogen at the benzylic position is sterically close to the bridgehead radical. Therefore, we considered that placing a radical acceptor at the benzylic position might promote an intramolecular radical addition reaction. As such a radical acceptor, the readily prepared alkoxy acrylate **29** was designed (Figure 3).

**Scheme 5:**
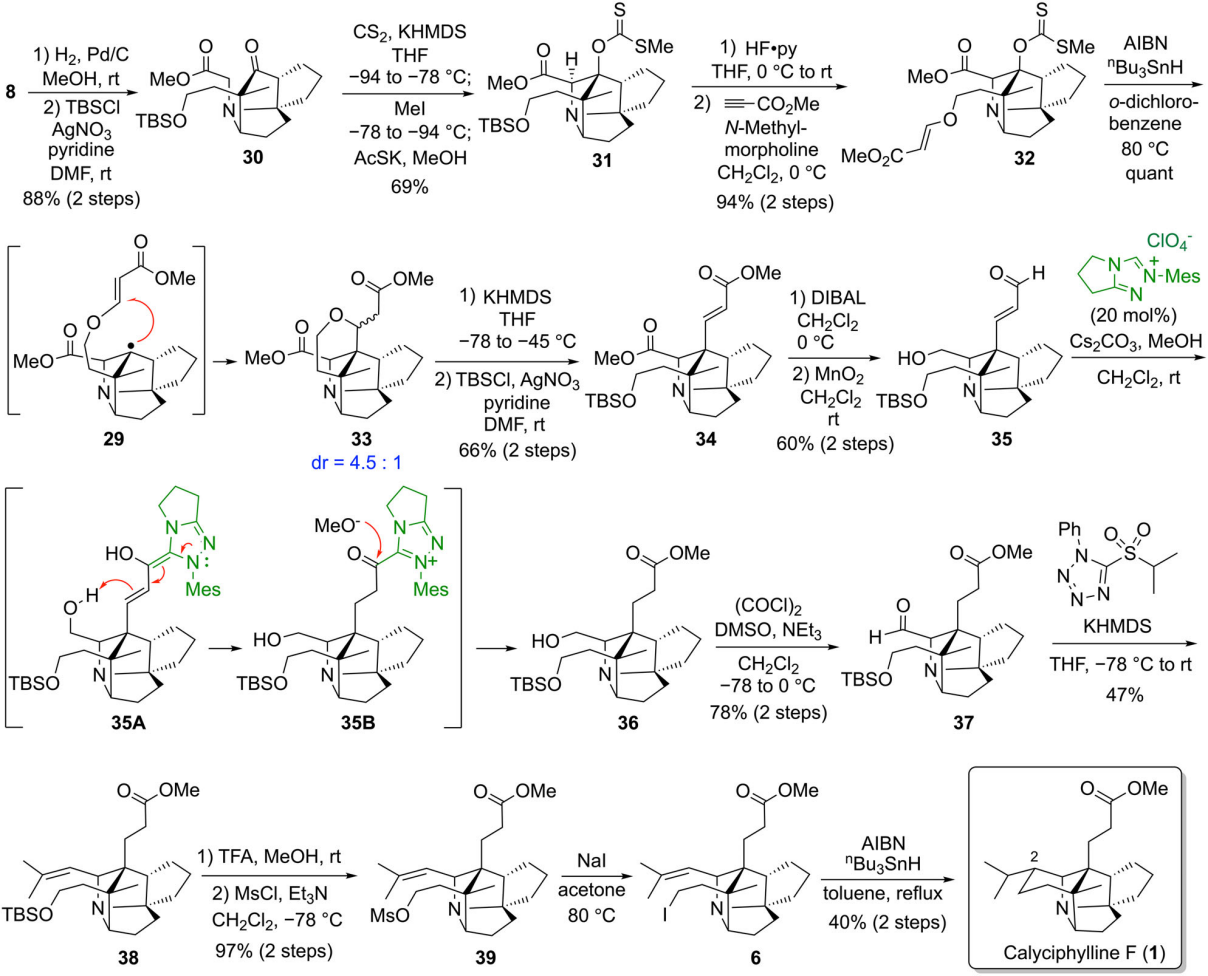
Total synthesis of calyciphylline F.

Since it was difficult to remove the benzyl protecting group of the primary hydroxy group in the presence of the xanthate at the bridgehead position, the benzyl group was converted to the TBS group along with hydrogenation of the double bond at the stage of the intramolecular aldol reaction precursor **8**, leading to **30**. The similar intramolecular aldol reaction of **30** afforded the tetracyclic xanthate **31** in 69% yield. After removal of the TBS group of **31** by HF‐pyridine treatment, the addition reaction of the resulting primary alcohol to methyl propiolate afforded the radical cyclization precursor **32** in good yield. With the new precursor in hand, the radical cyclization reaction of **32** was carried out under conditions similar to those used before. This time the formation of the bridgehead radical **29** followed by its addition to the alkene moiety proceeded smoothly to give the desired cyclized product **33** as a 4.5:1 diastereomeric mixture in quantitative yield. This approach allowed us to construct the two contiguous quaternary carbon centers containing a bridgehead quaternary carbon. The pyran ring of the cyclized product **33** was cleaved by E1cB elimination using KHMDS, and the resulting primary alcohol was protected with a TBS group to give **34**. The DIBAL reduction of **34** converted two methyl esters to alcohols, and the allylic alcohol was selectively oxidized by MnO_2_ to afford an unsaturated aldehyde **35** in 60% yield in two steps. Next, the unsaturated aldehyde of **35** was converted to a saturated methyl ester by the application of the NHC‐catalyzed method reported by Berkessel et al.^[^ ^62^ ^]^ The reaction proceeded through the formation of the Breslow intermediate **35A** from the condensation of **35** with an NHC catalyst, protonation of **35A** to give **35B**, and addition of methanol to **35B** to give the saturated methyl ester **36**. The resulting **36** was used for the subsequent Swern oxidation without purification due to its high polarity, and the aldehyde **37** was obtained in 78% yield from **35**. Then, the isopropylidene group was formed by Julia–Kocienski reaction to give **38** in 47% yield. The TBS group of **38** was removed and the resulting primary alcohol was converted to mesylate **39**. After roughly and quickly purifying the unstable **39** by short‐pass silica gel column chromatography, the mesylate was replaced with iodide to give the radical cyclization precursor **6**. Since synthetic intermediates **39** and **6** were highly unstable and decomposed over time, they had to be immediately used for the next reaction without purification. Finally, treatment of crude **6** with *^n^* Bu_3_SnH and AIBN under the reflux conditions in toluene allowed the radical cyclization reaction to proceed smoothly to give calyciphylline F (**1**). In this reaction, the stereoisomer of the isopropyl group at the C2 position was also obtained as a 1:1 mixture, and the pure **1** was obtained in 40% yield from the mesylate **39** after the separation of the diastereomer at the C2 position using amine‐coated preparative TLC.^[^ ^63^ ^]^ The spectral data (^1^H and ^13^C NMR, HRMS) of synthetic **1** were identical to those reported previously for natural **1**.^[^ ^4^, ^33^ ^]^ Thus, the first total synthesis of calyciphylline F was accomplished (Scheme 5).

## Conclusion

We succeeded in constructing a strained 8‐azatricyclo[4.2.1.0^4,8^]nonane ring skeleton through the intramolecular [4 + 3] cycloaddition reaction followed by the intramolecular aldol reaction. Based on the proposed mechanism of by‐product formation, the bridgehead quaternary carbon center was constructed, and the first total synthesis of calyciphylline F was achieved. The total synthesis in this study does not align with recent trends such as short‐step synthesis, large‐scale synthesis, applications of novel catalytic asymmetric reactions and new devices, and so on. However, for truly challenging compounds, achieving synthesis is the first critical step. Information in the first total synthesis can often be a key factor in future efficient and innovative total syntheses. Calyciphylline D‐type alkaloids promote expression of the mRNA encoding neuronal growth factor. We plan to elucidate the mode of action and the development of lead compounds for the treatment of neurodegenerative disease.

## Supporting Information

The authors have cited additional references within the Supporting Information.

## Conflict of Interests

The authors declare no conflict of interest.

## Supporting information



Supporting Information

## Data Availability

The data that support the findings of this study are available in the Supporting Information of this article.
