# Supplementary material for: Total Synthesis of Calyciphylline F
Source: Angew Chem Int Ed Engl. 2025 Sep 15;64(45):e202517671. doi: 10.1002/anie.202517671 (PMC12582000; doi:10.1002/anie.202517671)

Supporting Information

Total Synthesis of Calyciphylline F

Ryota Sato, Ryuichi Sumida, Masaki Inoue, Ryota Kotaka, Sangita Karanjit, Kosuke Namba*

[1]Department of Pharmaceutical Sciences, Tokushima University, 1-78-1 Shomachi, Tokushima 770-8505, Japan,

Table of Contents

General Procedures……………………………………………………………… S2
Supporting Information Tables……………………………………………………....S3
Supporting Information Schemes……………………………………………..…S5
Supporting Information Discussion…………………………………………..…S8
Experimental Procedure for Compounds……………………………………...S9
^{1}H NMR Comparison of Synthetic and Natural Calyciphylline F…………………..S30
Spectra for Compounds………………………………………………………..S31

General Procedures.

All the reaction were carried out in a round-bottomed flask with an appropriate number of necks and side arms connected to a three-way stopcock and/or a rubber septum cap under an argon atmosphere. All vessels were first evacuated by rotary pump and then flushed with argon prior to use. Solution and solvent were introduced by hypodermic syringe through a rubber septum. During the reaction, the vessel was kept under a positive pressure of argon. Dry THF was freshly prepared by distillation from benzophenone ketyl before use. Anhydrous CH_2Cl_2, DMF, ethanol, MeCN, methanol, pyridine and toluene were purchased from Kanto Chemical Co. Inc. Photoreactions were carried out in a test tube. A 10 W Blue LED (PER-448 nm, Techno Sigma) was plugged directly into the reaction mixture in a test tube.

Infrared (IR) spectra were recorded on JASCO FT/IR-4100 spectrophotometer using 5 mm KBr plate. Wavelengths of maximum absorbance are quoted in cm^{-1}. ^{1}H-NMR spectra were recorded on a JEOL ECZ500R (500 MHz) in $CDCl_3$, CD_3OD and C_6D_6. Chemical shifts are reported in part per million (ppm), and signal are expressed as singlet (s), doublet (d), triplet (t), quartet (q), multiplet (m) and broad (br). ^{13}C-NMR spectra were recorded on a JEOL ECZ500R (500 MHz) in $CDCl_3$, CD_3OD and $(CD_3)_2CO$. Chemical shifts are reported in part per million (ppm). Mass spectra were recorded on a Waters/Micromass LCT PREMIER (TOF). Analytical thin layer chromatography (TLC) was performed using 0.25 mm E. Merck Silica gel (60F-254) plates. Reaction components were visualized phosphomolybdic acid or ninhydrin or *p*-anisaldehyde in 10% sulfuric acid in ethanol. Kanto Chem. Co. Silica Gel 60N (particle size 0.040–0.050 mm) was used for column chromatography.

Supporting Information Tables

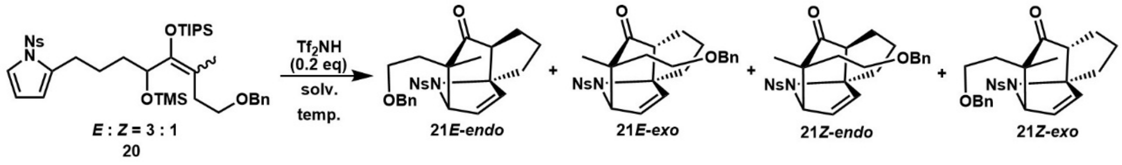

entry	solv.	temp.	yield	21*E*-*endo*	:	21*E*-*exo*	:	21*Z*-*endo*	:	21*Z*-*exo*
1	DCM	−78 ℃	68%	7.8		3		3		1
2	HFIP	0 ℃	trace	3		18		2		1
3	DCM/toluene	−78 ℃	56%	5.1		3		2.5		1
4	$MeNO_2$	0 ℃	48%	12		3		6		1
5	$CHCl_3$	−50 ℃	65%	5.1		3		2		1
6	DCM/$MeNO_2$	−50 ℃	59%	10		3		3.3		1
7	$EtNO_2$	−78 ℃	57%	15		3		2		0.5
8	$^nPrNO_2$	−78 ℃	57%	15		3		2		0.5
9	^nPrCN	−78 ℃	decomp.							
10	DCM	−94 to −50 ℃	68%	9		3		2.5		1

Table S1. Optimization of intramolecular [4+3] cycloaddition reaction. In this optimization study, the precursor **20** was used in a 3 : 1 *E*/*Z* mixture. After investigating reactions at various temperature and in various solvent, it was found that -78 °C in dichloromethane gave the best selectivity and yield. HFIP, hexafluoroisopropanol; $MeNO_2$, nitromethane; $EtNO_2$, nitroethane; $^nPrNO_2$, 1-nitropropane; ^nPrCN, 1-butyronitrile.

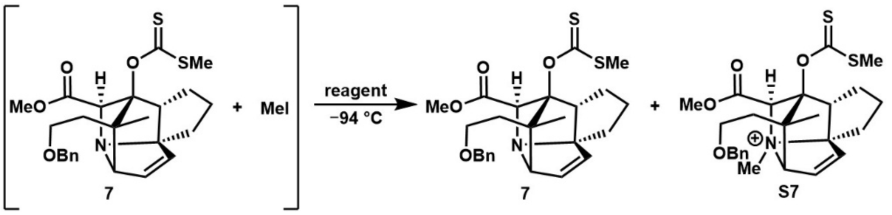

entry	reagent	7	:	S7
1	AcOK	-		major
2	DABCO	-		major
3	*N*-methylmorpholine	-		major
4	sparteine	-		major
5	MeOK	-		major
6	AcSK	-		major
7	AcSK in MeOH	major		-

Table S2. Investigation of a trapping reagent for methyl iodide. Various nucleophiles were investigated to react with excess methyl iodide, and only potassium thioacetate (AcSK) reacted with excess methyl iodide before bridgehead nitrogen of **7**. AcSK takes a while to dissolve in tetrahydrofuran, and during this time the bridgehead nitrogen is methylated, so it was necessary to add it as a solution in methanol. AcOK, potassium acetate; DABCO, 1,4-diazabicylco[2.2.2]octane. MeOK, potassium methoxide.

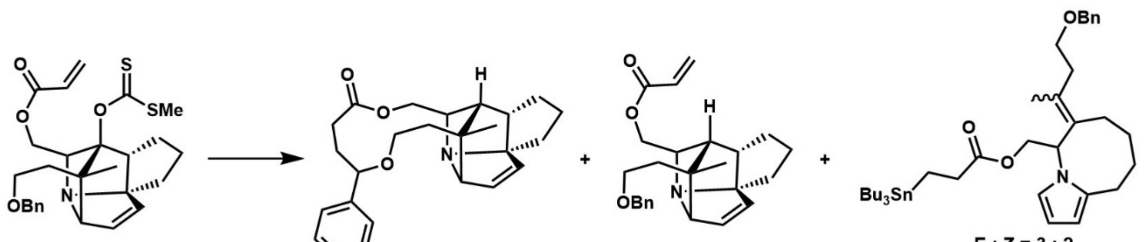

E : Z = 3 : 2

25 S8 S9 26

entry	reagent	solv.	temp. (°C)	ratio		
				S8	S9	26
1	V-40, Bu_3SnH	benzene	180, MW	-	major	-
2	AIBN, Bu_3SnH	benzene	80	-	major	-
3[a]	AIBN, Bu_3SnH	benzene	80	trace	-	major
4	AIBN, Bu_3SnH	o-dichlobenzene	80	trace	-	major
5	AIBN, Bu_3SnH	MeCN	80	1	trace	1
6	AIBN, Bu_3SnH	DMF	80	trace	trace	major
7	AIBN, Bu_3SnH	1,4-dioxane	80	trace	trace	major
8	AIBN, Bu_3SnH	^{t}BuOH	80	trace	trace	major
9	BPO, Bu_3SnH, UV	benzene	0 °C to rt	N.R.		
10	Et_3B, Bu_3SnH, O_2	benzene	rt ro 70 °C	SM and partly decomp.		

[a] syringe pump

Table S3. Investigation of 7-endo-radical cyclization reaction. Although the reaction was affected by the solvent, the desired cyclization product was not obtained at all. V-40, 1,1'-azobis(cyclohexane-1-carbonitrile); AIBN, 2,2'-azabis(isobutyronitrile); BPO, benzoyl peroxide; DMF, *N,N*-dimethylformamide .

Supporting Information Schemes

S1-1 Mukaiyama aldol route

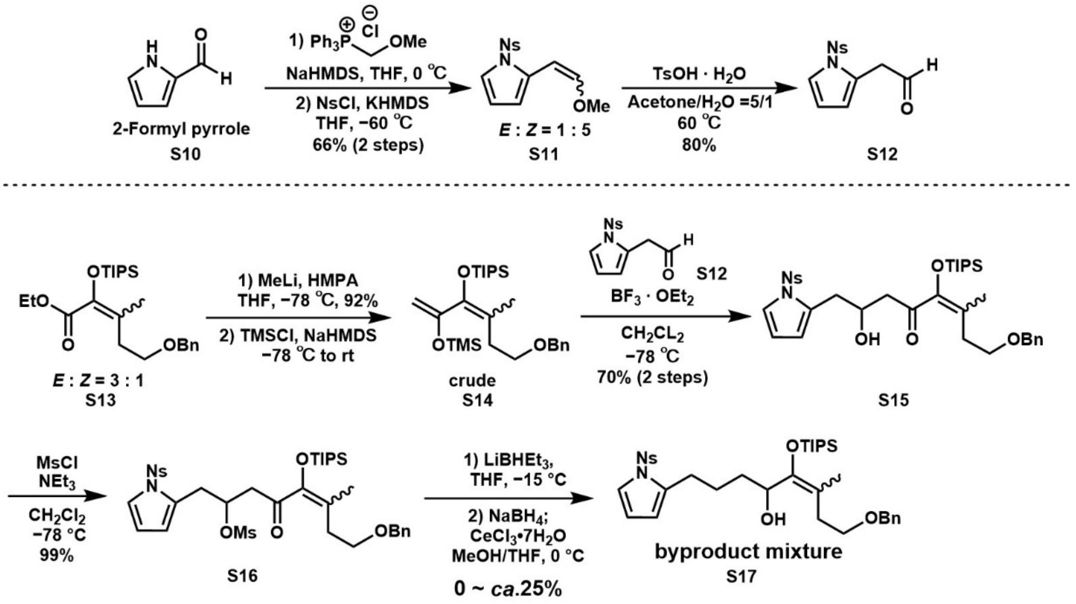

S1-2 Alkylation route

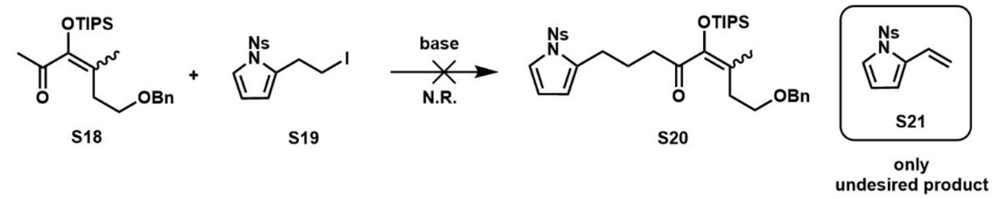

S1-3 Anion coupling route

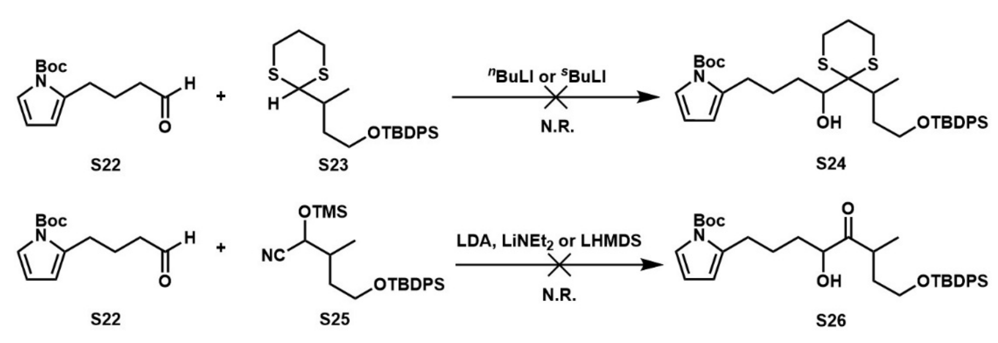

S1-4 Ynone route

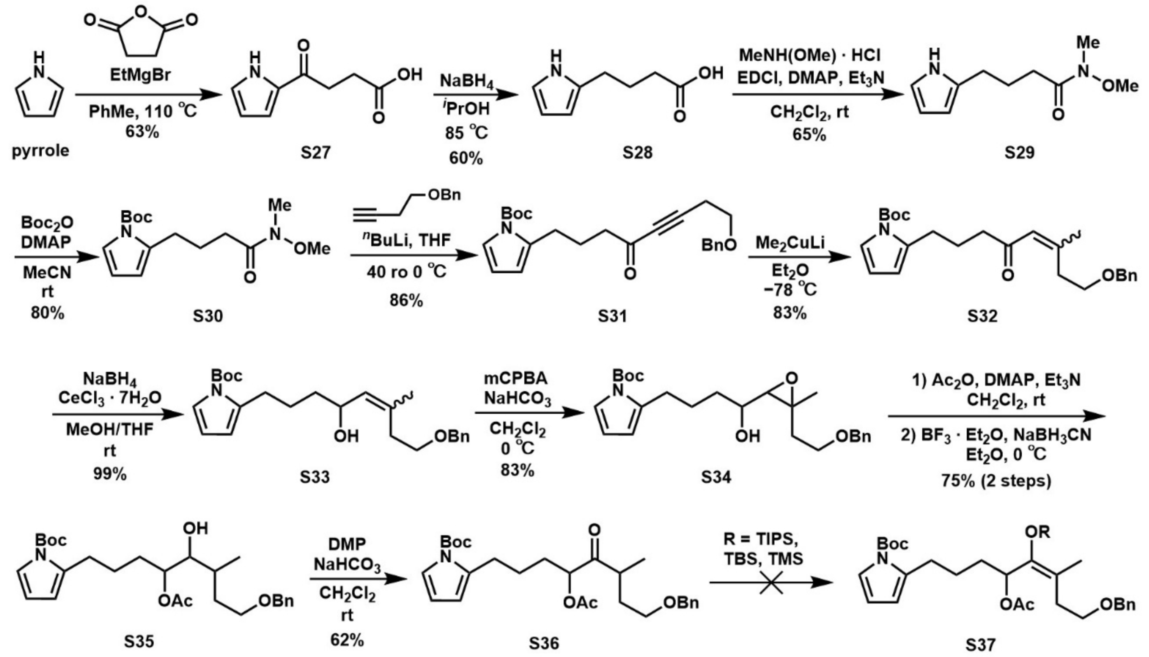

S1-5 cross metathesis route

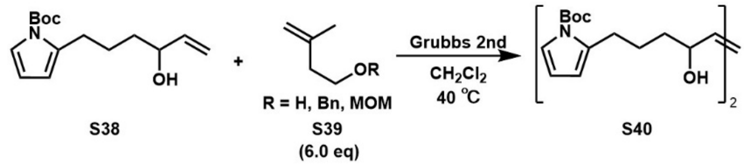

Scheme S1. Other routes that were not used for the synthesis of the [4+3] cycloaddition reaction precursor. Although the Mukaiyama aldol route could provide the cycloaddition precursor, the reproducibility was not good due to competition with the elimination reaction of mesylate, which preferentially gave the alkene conjugated with pyrrole. Other routes could not reach the cyclization precursor. NaHMDS, sodium bis(trimethylsilyl)amide; KHMDS, potassium bis(trimethylsilyl)amide; NsCl, 2-nitrobenzenesulfonyl chloride; TsOH, p-toluenesulfonic acid; HMPA, hexamethyl phosphorictriamide; TMSCl, trimethylsilyl chrolide; MsCl, methanesulfonic chloride; LDA, lithium diisopropylamide; EDCI, 1-(3-dimethylaminopropyl)-3-ethylcarbodiimide; DMAP, 4-dimethylaminopyridine; *m*CPBA, 3-chloroperoxybenzoic acid; DMP, Dess-Martin periodinane.

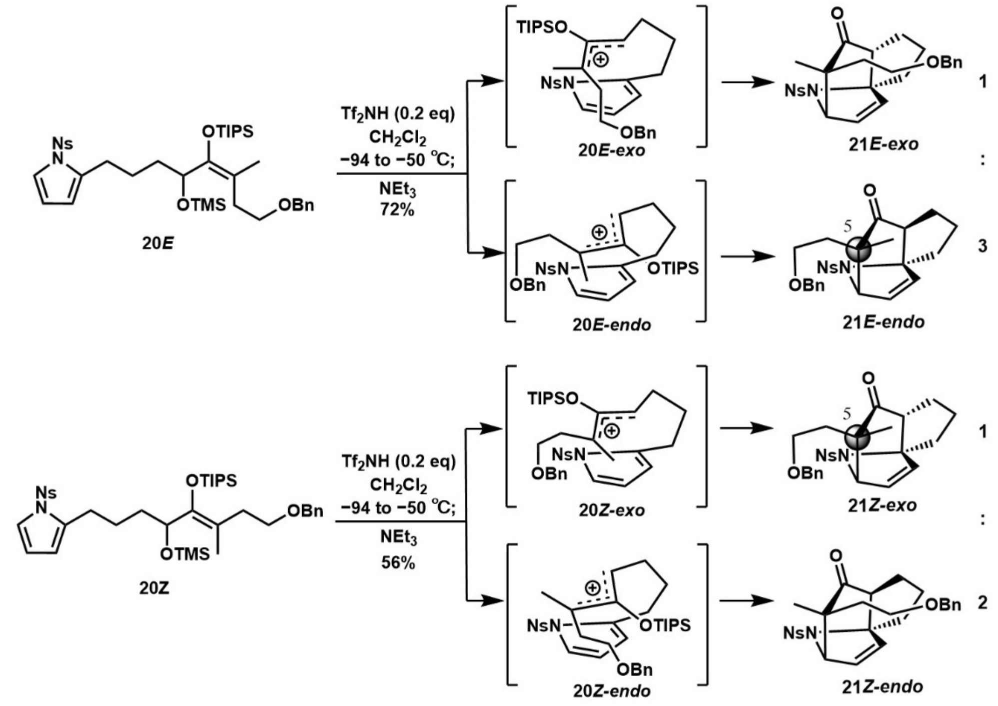

Scheme S2. Intramolecular [4+3] cycloaddition reaction from each isomer. In the intramolecular cycloaddition reaction of **20*E*,** the ratio of exo- to endo-isomers was 1 : 3. In the reaction of **20*Z***, the ratio of exo- to endo-isomers was 2 : 1.

Tf_2NH, bis(trifluoromethanesulfonyl)imide; NEt_3, triethylamine.

Supporting Information Discussions

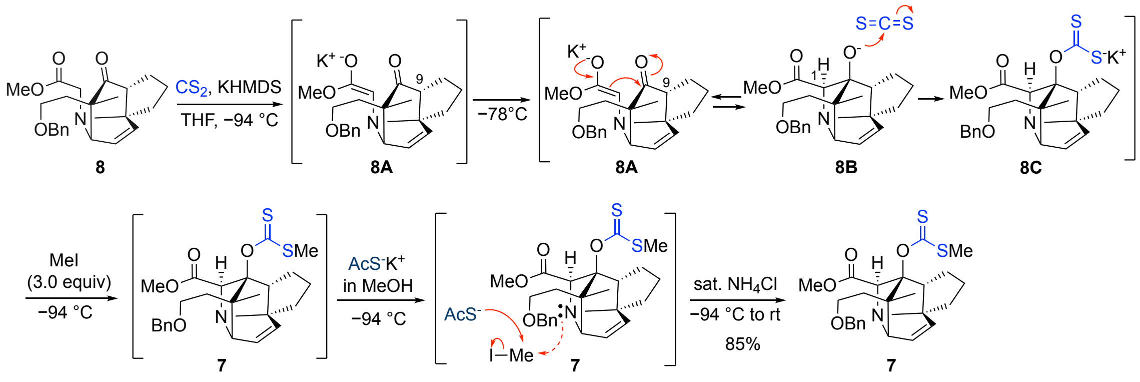

Explanation of the details of the complex operation in the intramolecular aldol reaction to construct the strained 8-azatricyclo[4.2.1.0^{4,8}]nonane ring core.

A mixture of **8** and CS_2 was treated with KHMDS at -94°C to generate ester enolate **8A**, and the mixture was warmed to -78°C to allow the intramolecular aldol reaction to proceed. The aldol adduct **8B** and the ester enolate **8A** were in equilibrium, and the alkoxide of **8B** was captured by CS_2 and converged from the equilibrium to potassium xanthate **8C**. Then, after cooling the reaction mixture again to -94°C, 3.0 equiv of methyl iodide was added and the alkylation proceeded to form xanthate **7**. The subsequent sequential operation of adding potassium thioacetate, stirring, and quenching the reaction with sat. NH_4Cl aqueous solution afforded the xanthate **7**, which contained the desired 8-azatricyclo[4.2.1.0^{4,8}]nonane ring skeleton, in 85% yield.

The details of the complex operation in this intramolecular aldol reaction are explained below. In the case that the initial treatment of KHMDS was performed at -78°C, enolization at the C9 position was also induced. Thus, it was necessary to raise the temperature to -78°C after selectively generating the kinetically favorable ester enolate at -94°C. After the intramolecular aldol reaction and subsequent formation of potassium xanthate **8C** at -78°C, the subsequent addition of methyl iodide at the same temperature induced the methylation of the bridgehead nitrogen. Thus, the methylation reaction had to be performed after cooling to -94°C again. After confirming the formation of **7**, the direct quench of the reaction by the addition of sat. NH_4Cl solution also induced the methylation of the nitrogen by the remaining methyl iodide due to the increase in temperature. Since 3.0 equiv of methyl iodide was required to complete the reaction, it was necessary to decompose the remaining methyl iodide before quenching. After examining various nucleophiles that decompose methyl iodide (Table S2), we found that potassium thioacetate was the most efficient for this purpose.

Experimental Procedure for Compounds

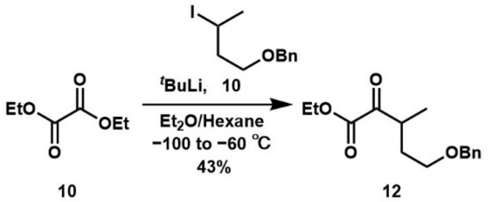

Compound **12**:

To a solution of *t*-BuLi (1.64 M solution in *n*-pentane, 100 mL, 157.2 mmol) in hexane (160 mL) and Et_2O (55 mL) was added a solution of iodoalkane **10** (19.0 g, 65.5 mmol) in hexane (80 mL) and Et_2O (25 mL) at –100 °C. The reaction mixture was stirring at –100 °C for 10 min and warmed up to –60 °C. After stirring at –60 °C for 30 min, to the mixture was slowly added diethyl oxalate **10** (15.9 mL, 117.9 mmol). The reaction mixture was stirred at –60 °C for 30 min, the reaction was quenched with saturated aqueous NH_4Cl at –60 °C and the mixture was extracted with AcOEt (x3). The combined organic layers were washed with brine, dried over $MgSO_4$, filtered, and concentrated under reduced pressure. The residue was purified by flash silica gel column chromatography (hexane/AcOEt = 50/1 to 10/1) to give **12** (7.15 g, 43%) as a yellow oil. ^1H NMR (500 MHz, $CDCl_3$): δ 7.35-7.30 (m, 2H), 7.29-7.24 (m, 3H), 4.42 (d, *J* = 12.1 Hz, 1H), 4.38 (d, *J* = 12.0 Hz, 1H), 4.23 (ddd, *J* = 14.3, 10.9, 7.2 Hz, 1H), 4.16 (ddd, *J* = 14.4, 10.9, 7.2 Hz, 1H), 3.52-3.38 (m, 3H), 2.06 (dtd, *J* = 14.4, 7.6, 5.0 Hz, 1H), 1.83 (ddd, *J* = 14.3, 10.6, 5.7 Hz, 1H), 1.27 (t, *J* = 7.2 Hz, 3H), 1.16 (d, *J* = 7.0 Hz, 3H); ^{13}C NMR (125 MHz, $CDCl_3$): δ 197.2, 161.4, 138.1, 128.4, 127.7, 127.7, 72.9, 67.6, 62.2, 39.5, 33.5, 15.9, 14.1; IR (KBr): 2978, 2873, 1725, 1454, 1365, 1273, 1096, 738, 698 cm^{-1}; HRMS-ESI (*m/z*): $[M+Na]^+$ calcd for $C_{15}H_{20}O_4Na$, 287.1254; found, 287.1261; R_f = 0.6 (silica gel, AcOEt/hexane = 1/4).

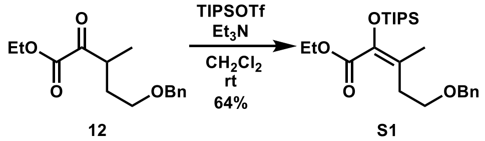

Compound **S1**:

To a solution of **12** (13.8 g, 52.0 mmol) in CH_2Cl_2 (134 mL) were added Et_3N (21.8 mL, 156 mmol) and TIPSOTf (21.0 mL, 78.0 mmol) at 0 °C. After stirring at room temperature for 12 h, the reaction was quenched with saturated aqueous $NaHCO_3$ at 0 °C and the mixture was extracted with CH_2Cl_2 (x3). The combined organic layers were dried over $MgSO_4$, filtered, and concentrated under reduced pressure. The residue was purified by flash silica gel column chromatography (hexane/Et_2O = 40/1 to 20/1) to give **S1** (14.0 g, 64%) as a yellow oil. ^1H NMR (500 MHz, $CDCl_3$): δ 7.34-7.31 (m, 4H), 7.27 (m, 1H), 4.50 (s, 2H), 4.19 (q, *J* = 7.2 Hz, 2H), 3.60 (t, *J* = 7.2, 2H), 2.70 (t, *J* = 7.2 Hz, 2H), 1.87 (s, 3H), 1.29 (t, *J* = 7.2 Hz, 3H), 1.23-1.14 (m, 3H), 1.08 (d, *J* = 7.2 Hz, 18H); ^{13}C NMR (125 MHz,

CDCl$_3$): δ 164.9, 138.6, 138.2, 128.2, 127.9, 127.5, 127.4, 72.7, 69.6, 60.4, 33.8, 19.2, 18.0, 14.1, 13.5; IR (KBr): 2944, 2866, 1714, 1627, 1464, 1389, 1293, 1179, 1084, 734, 681 cm^{-1}; HRMS-ESI (*m/z*): [M+Na]$^+$ calcd for $C_{24}H_{40}O_4SiNa$, 443.2588; found, 443.2591; R_f = 0.8 (silica gel, AcOEt/hexane = 1/4).

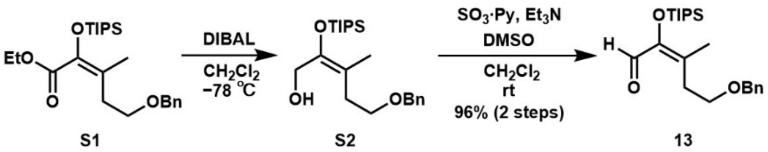

Compound **13**:

To a solution of **S1** (10.4 g, 24.8 mmol) in CH_2Cl_2 (124 mL) was added DIBAL (1.03 M solution in *n*-hexane, 57.8 mL, 59.5 mmol) at −78 °C. After stirring at −78 °C for 30 min, the reaction was quenched with MeOH (52 mL). To the mixture was added saturated aqueous solution of potassium tartrate (93 mL) at −78 °C and the mixture was warmed up to room temperature. The reaction mixture was stirred at room temperature for 45 min and extracted with Et_2O (x3). The combined organic layers were washed with brine, dried over $MgSO_4$, filtered, and concentrated under reduced pressure to give crude **S2** (10.0 g) as a colorless oil. The crude **S2** was used for next reaction without further purification.

To a solution of **S2** in CH_2Cl_2 (124 mL) were added Et_3N (34.6 mL, 248 mmol), DMSO (38.4 mL, 496 mmol) and SO_3·Py (19.7 g, 124 mmol) at 0 °C. After stirring at room temperature for 10 h, the reaction was quenched with saturated aqueous NH_4Cl at 0 °C and the mixture was extracted with Et_2O (x3). The combined organic layers were dried over $MgSO_4$, filtered, and concentrated under reduced pressure. The residue was purified by flash silica gel column chromatography (AcOEt/hexane = 1/40) to give **13** (9.0 g, 96% for 2 steps) as a yellow oil. ^{1}H NMR (500 MHz, CDCl$_3$): δ 9.85 (s, 1H), 7.36-7.30 (m, 2H), 7.30-7.25 (m, 3H), 4.50 (s, 2H), 3.59 (t, *J* = 6.5 Hz, 2H), 2.78 (t, *J* = 6.5 Hz, 2H), 1.96 (s, 3H), 1.25 (sept, *J* = 7.5 Hz, 3H), 1.05 (d, *J* = 7.5 Hz, 18H); ^{13}C NMR (125 MHz, CDCl$_3$): δ 184.9, 147.9, 138.2, 137.1, 128.5, 127.7, 127.6, 73.1, 68.6, 32.2, 18.7, 18.3, 14.1; IR (KBr): 2943, 2865, 1674, 1620, 1463, 1381, 1311, 1205, 1100, 734, 678 cm^{-1}; HRMS-ESI (*m/z*): [M+Na]$^+$ calcd for $C_{22}H_{36}O_3SiNa$, 399.2326; found, 399.2315; R_f = 0.7 (silica gel, AcOEt/hexane = 1/4).

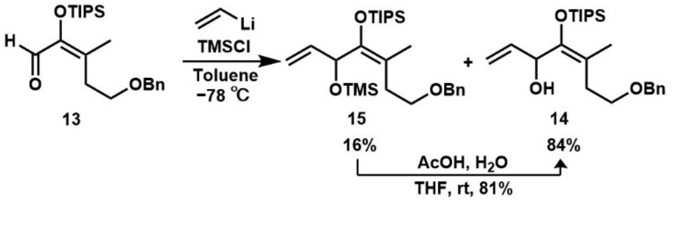

Compound **14, 15**:

To a solution of **13** (9.0 g, 23.9 mmol) in toluene (221 mL) was added TMSCl (10.8 mL, 71.7 mmol) at −78 °C. After stirring for 2 h at −78 °C, to the mixture was slowly added a solution of vinyl lithium (1.45 M solution in THF, 1.32 mL, 1.91 mmol). After stirring for 15 min at −78 °C, the reaction was quenched with saturated aqueous $NaHCO_3$ solution, and the mixture was extracted with AcOEt (x3). The combined organic layers were dried over $MgSO_4$, filtered, and concentrated under reduced pressure. The residue was purified by flash silica gel column chromatography (AcOEt/hexane = 1/50 to 1/20) to give **15** (1.8 g, 16%) and **14** (8.1 g, 84%) as a colorless oil. **15**: ^{1}H NMR (500 MHz, $CDCl_3$): δ 7.36-7.31 (m, 4H), 7.28 (m, 1H), 5.96 (ddd, *J* = 16.9, 10.2, 6.3 Hz, 1H), 5.11 (d, *J* = 17.2 Hz, 1H), 5.03 (d, *J* = 10.2 Hz, 1H), 4.93 (d, *J* = 6.3 Hz, 1H), 4.50 (s, 2H), 3.47 (t, *J* = 7.5 Hz, 2H), 2.46 (dt, *J* = 13.6, 7.4 Hz, 1H), 2.33 (dt, *J* = 13.6, 7.4 Hz, 1H), 1.65 (s, 3H), 1.26 (sept, *J* = 7.6 Hz, 1H), 1.09 (d, *J* = 7.4 Hz, 9H), 1.08 (d, *J* = 7.5 Hz, 9H) 0.09 (s, 9H); ^{13}C NMR (125 MHz, $CDCl_3$): δ 147.6, 139.6, 138.6, 128.5, 127.8, 127.7, 114.9, 110.8, 73.0, 71.8, 69.4, 33.4, 18.6, 18.4, 17.4, 13.8, 0.41; IR (KBr): 2945, 2866, 1661, 1465, 1362, 1251, 1196, 1099, 882, 734, 697 cm^{-1}; HRMS-ESI (*m/z*): $[M+H]^+$ calcd for $C_{27}H_{49}O_3Si_2$, 477.3215; found, 477.3230; R_f = 0.8 (silica gel, AcOEt/hexane = 1/4).

14: ^{1}H NMR (500 MHz, $CDCl_3$): δ 7.36-7.25 (m, 5H), 6.02 (ddd, *J* = 17.1, 10.4, 4.8 Hz, 1H), 5.34 (dt, *J* = 17.2, 1.8 Hz, 1H), 5.12 (dt, *J* = 10.5, 1.8 Hz, 1H), 4.71 (brs, 1H), 4.52 (d, *J* = 12.0 Hz, 1H), 4.49 (d, *J* = 11.9 Hz, 1H), 3.62 (brd, *J* = 4.4 Hz, 1H), 3.53-3.45 (m, 2H), 2.78 (ddd, *J* = 14.4, 8.5, 5.8 Hz, 1H), 2.06 (dt, *J* = 14.5, 4.5 Hz, 1H), 1.66 (s, 3H), 1.25-1.14 (m, 3H), 1.09 (d, *J* = 7.1 Hz, 18H); ^{13}C NMR (125 MHz, $CDCl_3$): δ 148.5, 138.9, 137.7, 128.5, 128.0, 127.9, 114.3, 111.7, 73.5, 73.5, 67.6, 32.4, 18.3, 18.2, 16.7, 13.7; IR (KBr): 3447, 2944, 2891, 1628, 1458, 1363, 1251, 1196, 1098, 884, 840, 733, 681 cm^{-1}; HRMS-ESI (*m/z*): $[M+Na]^+$ calcd for $C_{24}H_{40}O_3SiNa$, 427.2639; found, 427.2639; R_f = 0.65 (silica gel, AcOEt/hexane = 1/4).

To a solution of **15** (1.8 g, 3.88 mmol) in THF (38.5 mL) was added H_2O (8.64 mL) and AcOH (8.64 mL) at room temperature. After stirring for 24 h at room temperature, the reaction was quenched with saturated aqueous $NaHCO_3$ solution at 0 °C, and the mixture was extracted with AcOEt (x3). The combined organic layers were dried over anhydrous $MgSO_4$, filtered, and concentrated under reduced pressure. The residue was purified by flash silica gel column chromatography (AcOEt/hexane = 1/50 to 1/20) to give **14** (1.3 g, 81%) as a colorless oil.

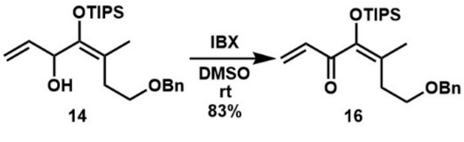

Compound **16**:

To a solution of **14** (8.6 g, 21.3 mmol) in DMSO (213 mL) was added IBX (11.9 g, 42.5 mmol) at room temperature. After stirring for 1 h, the reaction mixture was quenched with saturated aqueous $NaHCO_3$ solution, and the mixture was extracted with Et_2O (x3). The combined organic layers were dried over $MgSO_4$, filtered, and concentrated under reduced pressure. The residue was purified by flash silica gel column chromatography (AcOEt/hexane = 1/40) to give **16** (7.1 g, 83%) as a yellow oil. ^{1}H NMR (500 MHz, $CDCl_3$): δ 7.35-7.30 (m, 4H), 7.27 (m, 1H), 6.74 (dd, J = 17.4, 10.4 Hz, 1H), 6.33 (dd, J = 17.5, 1.7 Hz, 1H), 5.78 (dd, J = 10.5, 1.8 Hz, 1H), 4.49 (s, 2H), 3.61 (t, J = 6.9 Hz, 2H), 2.49 (d, J = 6.9 Hz, 2H), 1.85 (s, 3H), 1.16-1.02 (m, 3H), 1.06 (d, J = 6.3 Hz, 18H); ^{13}C NMR (125 MHz, $(CD_3)_2CO$): δ 191.3, 146.4, 139.9, 136.1, 129.4, 129.0, 128.2, 128.1, 124.6, 73.1, 70.2, 33.8, 18.3, 17.8, 14.0; IR (KBr): 2945, 2867, 1679, 1608, 1464, 1272, 1198, 1100, 990, 883, 734, 696 cm^{-1}; HRMS-ESI (m/z): $[M+H]^+$ calcd for $C_{24}H_{39}O_3Si$, 403.2663; found, 403.2678; R_f = 0.7 (silica gel, AcOEt/hexane = 1/4).

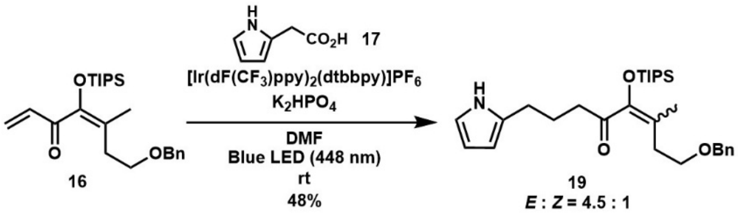

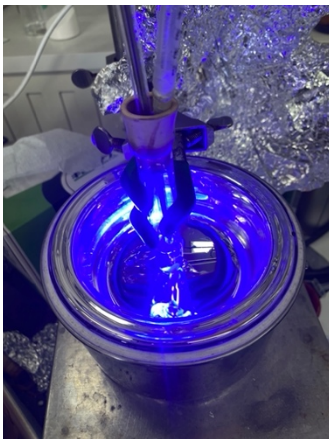

Compound **19**:

To a solution of **16** (100 mg, 0.248 mmol) and **17** (93.0 mg, 0.744 mmol) in DMF (3.5 mL) were added K_2HPO_4 (130 mg, 0.744 mmol) and $Ir[dF(CF_3)ppy)]_2(dtbbpy)PF_6$ (2.5 mg, 2.48 μmol) at room temperature. The mixture was stirred under irradiation of 448 nm LED light in a water bath for 20 min as shown in the picture above, the reaction mixture was quenched with saturated aqueous $NaHCO_3$ solution and extracted with Et_2O (x3). The combined organic layers were dried over $MgSO_4$, filtered, and concentrated under reduced pressure. The residue was purified by flash silica gel column chromatography (AcOEt/hexane = 1/40 to 1/20) to give **19** (57.8 mg, 48%, E:Z = 4.5:1) as a yellow oil. ^{1}H NMR (500 MHz, $CDCl_3$): δ 8.14 (brs, 0.22H), 8.05 (brs, 1H), 7.37-7.30 (m, 4.88H), 7.30-7.23 (m, 1.22H), 6.64 (m, 1H), 6.63 (m, 0.22H), 6.10 (dd, J = 5.8, 2.9 Hz, 1.22H), 5.90 (brs, 1.22H), 4.51 (s, 0.44H), 4.49 (s, 2H), 3.60 (t, J = 6.8 Hz, 2H), 3.58 (t, J = 7.1 Hz, 0.44H), 2.65 (t, J = 7.3 Hz, 2H), 2.63 (t, J = 8.6 Hz, 0.44H), 2.59 (t, J = 7.5 Hz, 0.44H), 2.58 (t, J = 7.5 Hz, 2H), 2.53 (t, J = 7.0 Hz, 0.44H), 2.50 (t, J = 6.8 Hz, 2H), 1.89 (m, 0.44H), 1.88 (quint, J = 7.3 Hz, 2H), 1.82 (s, 0.66H), 1.81 (s, 3H), 1.16-1.00 (m, 25.62H); ^{13}C NMR (125 MHz, $CDCl_3$): δ 201.9, 146.5, 138.7, 131.7, 128.4, 127.7, 127.5, 123.8, 116.4, 108.3, 105.4, 72.9, 70.1, 39.7, 33.5, 27.0, 24.2, 18.4,

18.0, 13.6; IR (KBr): 3383, 2944, 1684, 1609, 1465, 1366, 1274, 1189, 1096, 1027, 883, 790, 697, 684 cm^{-1}; HRMS-ESI (*m/z*): [M+H]$^+$ calcd for $C_{29}H_{46}NO_3Si$, 484.3241; found, 484.3231; R$_f$ = 0.5 (silica gel, AcOEt/hexane = 1/4).

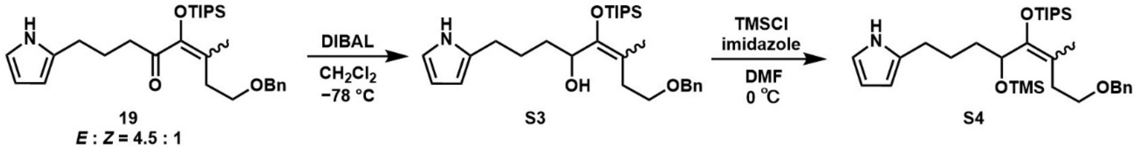

Compound **S4**:

To a solution of **19** (6.6 g, 13.6 mmol) in CH_2Cl_2 (97.4 mL) was slowly added DIBAL (1.0 M solution in hexane, 32.7 mL, 32.7 mmol) at −78 °C. After stirring at −78 °C for 25 min, the reaction mixture was quenched with MeOH (31.0 mL), and to the mixture was added saturated aqueous solution of potassium tartrate (48.0 mL) at −78 °C and the mixture was warmed up to room temperature. After stirring at room temperature for 45 min, the reaction mixture was extracted with Et_2O (x3). The combined organic layers were dried over $MgSO_4$, filtered, and concentrated under reduced pressure to give crude **S3** (7.3 g) as a colorless oil. The crude **S3** was used for next reaction without further purification.

To a solution of **S3** in DMF (68.2 mL) were added imidazole (2.2 g, 32.7 mmol) and TMSCl (3.8 mL, 30.0 mmol) at 0 °C, and the mixture was stirred for 20 min. The reaction was quenched with saturated aqueous $NaHCO_3$ solution, and the mixture was extracted with Et_2O (x3). The combined organic layers were dried over $MgSO_4$, filtered, and concentrated under reduced pressure. The residue was purified by flash silica gel column chromatography (AcOEt/hexane =1/50 to 1/30) to give **S4** (7.6 g, quant in 2 steps) as a brown oil. ^{1}H NMR (500 MHz, $CDCl_3$): δ 8.04 (brs, 0.22H), 7.92 (brs, 1H), 7.37-7.30 (m, 4.88H), 7.30-7.25 (m, 1.22H), 6.61 (brs, 1H), 6.53 (brs, 0.22H), 6.10 (dd, *J* = 5.6, 2.7 Hz, 1H), 6.06 (dd, *J* = 5.3, 2.5 Hz, 0.22H), 5.87 (brs, 1H), 5.85 (brs, 0.22H), 4.49 (s, 2.44H), 4.46 (t, *J* = 6.8 Hz, 1.22H), 3.53 (t, *J* = 7.3 Hz, 0.44H), 3.51-3.41 (m, 2H), 2.80 (dt, *J* = 13.2, 7.6 Hz, 0.22H), 2.53 (t, *J* = 7.2 Hz, 2.44H), 2.44 (ddd, *J* = 13.9, 8.9, 6.6 Hz, 1H), 2.33 (ddd, *J* = 14.2, 8.5, 6.0 Hz, 1H), 2.03 (dt, *J* = 13.3, 6.9 Hz, 0.22H), 1.76-1.66 (m, 1.22H), 1.70 (s, 0.66H), 1.64-1.40 (m, 3.66H), 1.55 (s, 3H), 1.24 (sept, *J* = 7.6 Hz, 3.66H), 1.08 (d, *J* = 7.4 Hz, 21.96H), 0.08 (s, 1.98H), 0.07 (s, 9H); ^{13}C NMR (125 MHz, $CDCl_3$): δ 147.9, 138.6, 132.6, 128.5, 127.7, 127.7, 116.1, 110.6, 108.2, 105.0, 73.0, 69.5, 35.2, 33.4, 27.7, 26.0, 18.5, 18.4, 17.0, 13.7, 0.38; IR (KBr): 3434, 2945, 2866, 1660, 1455, 1250, 1196, 1096, 1050, 884, 839, 697, 664 cm^{-1}; HRMS-ESI (*m/z*): [M+Na]$^+$ calcd for $C_{32}H_{55}NO_3Si_2Na$, 580.3613; found, 580.3616; R$_f$ = 0.7 (silica gel, AcOEt/hexane = 1/4).

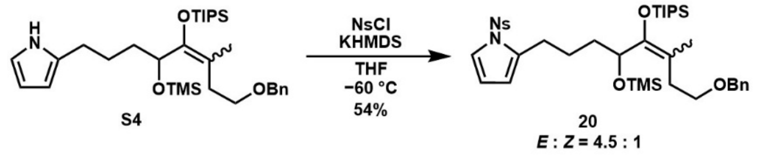

Compound **20**:

To a solution of **S4** (7.6 g, 13.6 mmol) in THF (136 mL) was added KHMDS (1.0 M solution in THF, 34.1 mL, 34.1 mmol) at –60 °C, and the mixture was warmed up to –50 °C. After the reaction mixture was stirred at –50 °C for 1 h and cooled to –78 °C, to the mixture was added a solution of NsCl (9.7 g, 43.7 mmol) in THF (20 mL). After stirring at –78 °C for 30 min, the reaction was quenched with saturated aqueous $NaHCO_3$ at –78 °C and the mixture was extracted with AcOEt (x3). The combined organic layers were dried over $MgSO_4$, filtered, and concentrated under reduced pressure. The residue was purified by flash silica gel column chromatography (AcOEt/hexane = 1/40 to 1/10) to give **20** (5.5 g, 54%, 4.5:1 *E*:*Z* mixture) as a brown oil. ^{1}H NMR (500 MHz, $CDCl_3$): δ 7.85 (d, *J* = 8.0 Hz, 0.22H), 7.84 (t, *J* = 7.0 Hz, 1H), 7.70 (t, *J* = 7.6 Hz, 0.22H), 7.70 (t, *J* = 8.5 Hz, 1H), 7.58 (t, *J* = 7.6 Hz, 1H), 7.56 (t, *J* = 8.0 Hz, 0.22H), 7.36-7.22 (m, 7.32H), 7.05 (d, *J* = 8.0 Hz, 1H), 7.01 (d, *J* = 8.1 Hz, 0.22H), 6.27 (t, *J* = 3.4 Hz, 1H), 6.25 (t, *J* = 3.4 Hz, 0.22H), 6.06 (brs, 1.22H), 4.48 (s, 2H), 4.47 (s, 0.44H), 4.39 (t, *J* = 6.8 Hz, 1.22H), 3.49-3.38 (m, 2.44H), 2.73 (dt, *J* = 13.0, 7.6 Hz, 0.22H), 2.51 (t, *J* = 7.6 Hz, 2.44H), 2.38 (ddd, *J* = 13.7, 8.9, 6.5 Hz, 1H), 2.29 (ddd, *J* = 14.5, 8.6, 6.2 Hz, 1H), 2.04 (dt, *J* = 13.6, 7.7 Hz, 0.22H), 1.72-1.33 (m, 4.88H), 1.63 (s, 0.66H), 1.61 (s, 3H), 1.29-1.15 (m, 3.66H), 1.05 (d, *J* = 6.5 Hz, 21.96H), 0.06 (s, 1.98H), 0.05 (s, 9H); ^{13}C NMR (125 MHz, $CDCl_3$); δ 147.7, 147.7, 138.6, 136.6, 134.5, 133.9, 132.9, 128.7, 128.5, 127.8, 127.7, 125.4, 123.7, 112.5, 111.7, 110.7, 73.1, 69.5, 35.2, 33.4, 27.3, 24.4, 18.6, 18.4, 17.1, 13.7, 0.4; IR (KBr): 2945, 2865, 1660, 1547, 1465, 1454, 1379, 1262, 1186, 1150, 1118, 1065, 883, 850, 741, 678 cm^{-1}; HRMS-ESI (*m/z*): [M+Na]$^+$ calcd for $C_{38}H_{58}N_2O_7SSi_2Na$, 765.3395; found, 765.3401; R_f = 0.2 (silica gel, AcOEt/hexane = 1/4).

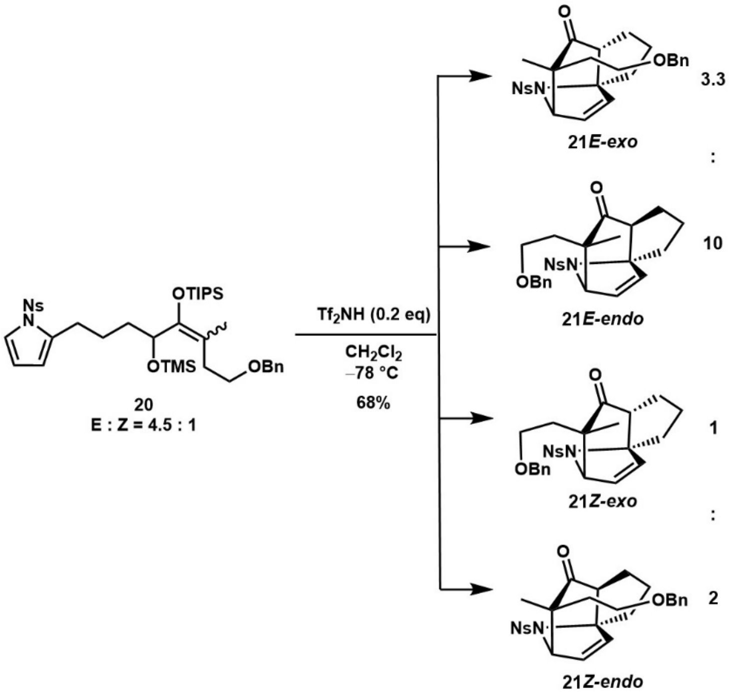

Compound **21*E-exo***, **21*E-endo***, **21*Z-exo***, **21*Z-endo***:

To a solution of **20** (2.0 g, 2.69 mmol) in CH_2Cl_2 (53.8 mL) was added a solution of Tf_2NH (151 mg, 0.538 mmol) in CH_2Cl_2 (1.13 mL) at –78 °C. After stirring at –50 °C for 30 min, to the mixture was slowly added NEt_3 (1.87 mL, 117.9 mmol). The reaction mixture was stirred at –50 °C for 30 min, the reaction was quenched with saturated aqueous $NaHCO_3$ at –50 °C and the mixture was extracted with AcOEt (x3). The combined organic layers were dried over $MgSO_4$, filtered, and concentrated under reduced pressure. The residue was purified by flash silica gel column chromatography (AcOEt/hexane = 1/40 to 1/4) to give an inseparable mixture of **21*E-exo***, **21*E-endo***, **21*Z-exo***, **21*Z-endo*** (0.91 g, **21*E-exo***, **21*E-endo***, **21*Z-exo***, **21*Z-endo*** = 3.3: 10: 1: 2) as a brown oil.

21*E-exo* and **21*E-endo*** (1 : 3): ^{1}H NMR (500 MHz, $CDCl_3$): δ 7.96 (d, *J* = 7.8 Hz, 0.75H), 7.71 (d, *J* = 8.1 Hz, 0.25H), 7.68-7.57 (m, 1.75H), 7.52 (d, *J* = 7.7 Hz, 0.75H), 7.46 (t, *J* = 7.8 Hz, 0.25H), 7.43 (d, *J* = 8.1 Hz, 0.25H), 7.36-7.20 (m, 5H), 6.25 (dd, *J* = 5.9, 1.5 Hz, 0.25H), 6.23 (dd, *J* = 6.0, 1.8 Hz, 0.75H), 5.93 (d, *J* = 5.9 Hz, 0.25H), 5.79 (d, *J* = 6.0 Hz, 0.75H), 4.94 (d, *J* = 1.5 Hz, 0.25H), 4.66 (d, *J* = 2.2 Hz, 0.75H), 4.54 (d, *J* = 11.6 Hz, 0.25H), 4.50 (d, *J* = 11.7 Hz, 0.25H), 4.32 (s, 1.5H), 3.71-3.59 (m, 0.50H), 3.56 (dt, *J* = 9.7, 4.8 Hz, 0.75H), 3.39 (td, *J* = 9.6, 4.3 Hz, 0.75H), 3.27 (dd, *J* = 11.2, 9.2 Hz, 0.75H), 2.76 (ddd, *J* = 13.9, 8.4, 2.3 Hz, 0.25H), 2.63 (dt, *J* =11.3, 10.8 Hz, 0.75H), 2.55 (ddd, *J* = 14.5, 9.1, 5.2 Hz, 0.75H), 2.41 (dd, *J* = 11.7, 7.9 Hz, 0.25H), 2.20-2.08 (m, 0.5H), 2.07-1.62 (m, 4.75H), 1.59 (ddd, *J* = 15.1, 9.4, 5.7 Hz, 0.25H), 1.48 (m, 0.75H), 1.34 (s, 0.75H), 1.05 (s, 2.25H); ^{13}C NMR (125 MHz, CD_3CD): δ 215.4, 210.8, 150.2, 150.0, 139.8, 139.6, 138.9, 137.1, 135.6, 135.6, 135.5, 134.7, 133.1, 133.0, 132.7, 132.5, 132.4, 131.7, 129.4, 129.3, 129.0, 128.9, 128.7, 128.6, 125.3, 125.1, 79.6, 78.3, 74.3, 74.0, 72.9, 71.6, 67.0, 63.0, 62.3, 55.3, 54.8, 53.5, 39.1, 34.9, 30.3, 30.2, 30.0,

24.0, 23.7, 21.7, 20.9, 18.8; IR (KBr): 2917, 2849, 1716, 1546, 1454, 1373, 1164, 1101, 743, 600 cm^{-1}; HRMS-ESI (*m/z*): $[M+Na]^+$ calcd for $C_{26}H_{28}N_2O_6SNa$, 519.1560; found, 519.1566; R_f = 0.6 (silica gel, AcOEt/hexane = 1/1).

21*Z-exo*: ^{1}H NMR (500 MHz, $CDCl_3$): δ 7.90 (dd, *J* = 8.0, 1.3 Hz, 1H), 7.67 (td, *J* = 7.7, 1.3 Hz, 1H), 7.60 (td, *J* = 7.8, 1.3 Hz, 1H), 7.52 (dd, *J* = 7.9, 1.3 Hz, 1H), 7.38-7.29 (m, 4H), 7.27 (m, 1H), 6.12 (dd, *J* = 6.0, 2.6 Hz, 1H), 5.97 (d, *J* = 5.9 Hz, 1H), 4.79 (d, *J* = 2.6 Hz, 1H), 4.47 (s, 2H), 3.64 (dt, *J* = 9.8, 6.6 Hz, 1H), 3.55 (dt, *J* = 9.8, 6.8 Hz, 1H), 2.82 (ddd, *J* = 14.6, 8.9, 2.8 Hz, 1H), 2.42 (dd, *J* = 11.6, 8.8 Hz, 1H), 2.24-2.12 (m, 2H), 2.12-1.98 (m, 3H), 1.92 (ddd, *J* = 14.5, 11.1, 7.5 Hz, 1H), 1.83 (m, 1H), 1.05 (s, 3H); ^{13}C NMR (125 MHz, $CDCl_3$): δ 212.6, 149.0, 138.6, 138.1, 133.9, 132.8, 132.7, 131.2, 131.0, 128.4, 127.9, 127.6, 124.2, 78.6, 73.0, 69.4, 66.1, 62.4, 53.9, 38.0, 29.7, 28.4, 22.8, 19.2; IR (KBr): 2917, 2849, 1700, 1545, 1453, 1373, 1161, 1100, 742, 621 cm^{-1}; HRMS-ESI (*m/z*): $[M+Na]^+$ calcd for $C_{26}H_{28}N_2O_6SNa$, 519.1560; found, 519.1566; R_f = 0.5 (silica gel, AcOEt/hexane = 1/1).

21*Z-endo*: ^{1}H NMR (500 MHz, $CDCl_3$): δ 7.87 (dd, *J* = 8.0, 1.2 Hz, 1H), 7.65 (td, *J* = 7.3, 1.4 Hz, 1H), 7.52 (d, *J* = 7.5 Hz, 1H), 7.51 (td, *J* = 7.6, 1.3 Hz, 1H), 7.40-7.32 (m, 4H), 7.30 (m, 1H), 6.37 (dd, *J* = 6.0, 2.3 Hz, 1H), 5.83 (d, *J* = 6.0 Hz, 1H), 5.03 (d, *J* = 2.4 Hz, 1H), 4.57 (d, *J* = 1.2 Hz, 2H), 3.72-3.63 (m, 2H), 3.25 (dd, *J* = 11.4, 8.5 Hz, 1H), 2.62 (dt, *J* = 12.2, 10.8 Hz, 1H), 2.09-1.96 (m, 3H), 1.92-1.72 (m, 2H), 1.57 (ddd, *J* = 15.0, 8.4, 6.4 Hz, 1H), 1.47 (dtd, *J* = 13.7, 11.5, 6.6 Hz, 1H), 1.31 (s, 3H); ^{13}C NMR (125 MHz, $CDCl_3$): δ 210.5, 148.6, 138.5, 136.6, 135.1, 133.8, 133.0, 131.4, 131.3, 128.6, 127.9, 127.7, 124.2, 76.4, 73.4, 70.9, 66.3, 59.8, 52.8, 33.2, 29.2, 21.3, 20.9, 20.2; IR (KBr): 2958, 2878, 1714, 1544, 1454, 1373, 1164, 1100, 741, 599 cm^{-1}; HRMS-ESI (*m/z*) $[M+Na]^+$ calcd for $C_{26}H_{28}N_2O_6SNa$, 519.1560; found, 519.1566; R_f = 0.63 (silica gel, AcOEt/hexane = 1/1).

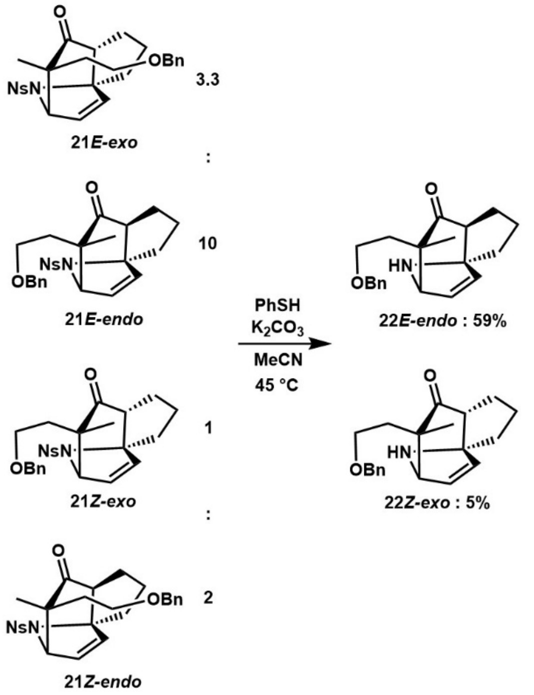

Compound **22*E-endo***, **22*Z-exo***:

To a solution of an inseparable mixture of **21*E-exo***, **21*E-endo***, **21*Z-exo***, **21*Z-endo*** (560 mg, 1.13 mmol) in MeCN (16.5 mL) were added K_2CO_3 (913 mg, 6.61 mmol) and PhSH (0.336 mL, 3.30 mmol) at room temperature, and the mixture was warmed up to 45 °C. After stirring at 45 °C for 12 h, the reaction was quenched with saturated aqueous NH_4Cl at 0 °C and the mixture was extracted with AcOEt (x3). The combined organic layers were dried over $MgSO_4$, filtered, and concentrated under reduced pressure. The residue was purified by flash silica gel column chromatography (AcOEt/hexane = 1/8 to AcOEt/MeOH = 3/1) to give **22*E-endo*** (207 mg, 59%) and **22*Z-exo*** (17 mg, 5%) as a yellow oil.

22*E-endo*: ^{1}H NMR (500 MHz, CD_3OD): δ 7.36-7.23 (m, 5H), 6.33 (dd, *J* = 5.8, 2.4 Hz, 1H), 6.07 (d, *J* = 5.9 Hz, 1H), 4.40 (d, *J* = 11.5 Hz, 1H), 4.36 (d, *J* = 11.5 Hz, 1H), 3.78 (d, *J* = 2.4 Hz, 1H), 3.60 (dt, *J* = 9.8, 5.3 Hz, 1H), 3.46 (ddd, *J* = 9.7, 8.4, 4.7 Hz, 1H), 3.01 (dd, *J* = 11.6, 7.7 Hz, 1H), 2.37 (ddd, *J* = 14.1, 8.4, 5.2 Hz, 1H), 1.96-1.74 (m, 5H), 1.65 (m, 1H), 1.48 (m, 1H), 0.99 (s, 3H); ^{13}C NMR (125 MHz, CD_3OD): δ 213.2, 139.7, 138.1, 137.0, 129.3, 128.9, 128.6, 76.3, 74.1, 70.4, 67.4, 59.7, 52.6, 38.8, 32.0, 22.5, 21.3, 19.2; IR (KBr): 2965, 2871, 1707, 1453, 1374, 1102, 911, 744, 697 cm^{-1}; HRMS-ESI (*m/z*): $[M+Na]^+$ calcd for $C_{20}H_{25}NO_2Na$, 334.1777; found, 334.1790; R_f = 0.2 (silica gel, AcOEt/hexane = 1/1).

22*Z-exo*: ^{1}H NMR (500 MHz, CD_3OD): δ 7.37-7.31 (m, 4H), 7.28 (m, 1H), 6.30 (dd, *J* = 5.6, 2.1 Hz, 1H), 6.27 (d, *J* = 5.6 Hz, 1H), 4.46 (s, 2H), 3.84 (d, *J* = 2.1 Hz, 1H), 3.66-3.54 (m, 2H), 2.32 (dd, *J* = 10.2, 8.9 Hz, 1H), 2.12 (dt, *J* = 14.0, 7.2 Hz, 1H), 2.05 (m, 1H), 2.01-1.90 (m, 2H), 1.90-1.65 (m, 4H), 0.95 (s, 3H); ^{13}C NMR (125 MHz, CD_3OD): δ 219.1, 142.0, 139.6, 136.4, 129.4, 129.0, 128.7, 74.8,

74.0, 68.1, 67.4, 59.4, 52.8, 40.3, 34.1, 29.9, 24.6, 21.6; IR (KBr): 2959, 2869, 1696, 1453, 1364, 1099, 835, 733, 697 cm^{-1}; HRMS-ESI (*m/z*): $[M+Na]^+$ calcd for $C_{20}H_{25}NO_2Na$, 334.1777; found, 334.1790; R_f = 0.5 (silica gel, AcOEt/hexane = 1/1).

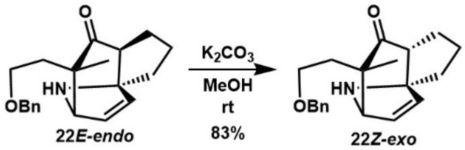

Compound **22*Z-exo***:

A solution of **22*E-endo*** (207 mg, 0.667 mmol) in MeOH (3.3 mL) was degassed by freeze-thaw procedure (x3), and then to the solution was added K_2CO_3 (118.3 mg, 0.856 mmol) at room temperature. After stirring at room temperature for 2 h, the reaction was quenched with saturated aqueous NH_4Cl at 0 °C and the mixture was extracted with AcOEt (x3). The combined organic layers were dried over $MgSO_4$, filtered, and concentrated under reduced pressure. The residue was purified by flash silica gel column chromatography (AcOEt/hexane = 1/8 to 1/2) to give **22*Z-exo*** (172 mg, 83%) as a yellow oil.

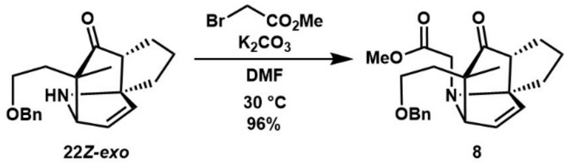

Compound **8**:

To a solution of **22*Z-exo*** (189 mg, 0.608 mmol) in DMF (3.0 mL) were added K_2CO_3 (166 mg, 1.20 mmol) and methyl bromoacetate (68.4 µL, 0.72 mmol) at room temperature. After stirring at 30 °C for 15 h, the reaction was quenched with saturated aqueous NH_4Cl at 0 °C and the mixture was extracted with Et_2O (x3). The combined organic layers were washed with brine, dried over $MgSO_4$, filtered, and concentrated under reduced pressure. The residue was purified by flash silica gel column chromatography (AcOEt/hexane = 1/10 to 1/6) to give **8** (224 mg, 96%) as a yellow oil. ^1H NMR (500 MHz, $CDCl_3$): δ 7.38-7.28 (m, 4H), 7.26 (m, 1H), 6.07 (dd, *J* = 5.5, 2.3 Hz, 1H), 5.99 (d, *J* =5.5 Hz, 1H), 4.48 (s, 2H), 3.72-3.64 (m, 1H), 3.67 (s, 3H), 3.61 (ddd, *J* = 10.1, 8.4, 6.9 Hz, 1H), 3.54 (ddd, *J* = 9.7, 7.8, 6.2 Hz, 1H), 3.33 (d, *J* = 15.7 Hz, 1H), 3.03 (d, *J* = 15.7 Hz, 1H), 2.35 (dd, *J* = 10.3, 9.4 Hz, 1H), 2.26 (ddd, *J* = 13.9, 7.9, 6.2 Hz, 1H), 2.17 (ddd, *J* = 14.0, 7.7, 6.6 Hz, 1H), 2.04-1.87 (m, 3H), 1.86-1.68 (m, 3H), 0.95 (s, 3H); ^{13}C NMR (125 MHz, $CDCl_3$); δ 216.1, 172.1, 138.7, 137.0,

131.9, 128.2, 127.6, 127.4, 77.6, 73.8, 72.8, 67.1, 59.8, 53.3, 51.6, 51.0, 38.5, 29.5, 27.8, 23.0, 19.8; IR (KBr): 2950, 2871, 1453, 1372, 1279, 1200, 1101, 735, 698 cm^{-1}; HRMS-ESI (*m/z*): $[M+Na]^+$ calcd for $C_{23}H_{29}NO_4Na$, 406.1989; found, 406.1977; R_f = 0.9 (silica gel, AcOEt/hexane = 1/1).

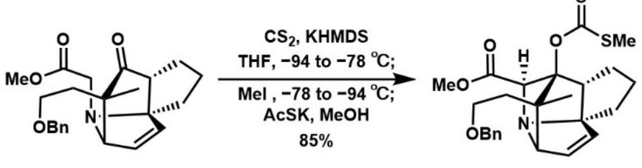

8 7

Compound **7**:

To a solution of **8** (50.0 mg, 0.130 mmol) in THF (1.30 mL) were added CS_2 (9.42 μL, 0.156 mmol) at room temperature and cooled to –94 °C. After stirring at –94 °C for 2 h, to the mixture was added KHMDS (1.0 M in THF solution, 196 μL, 0.196 mmol) at the same temperature, and the mixture was warmed up to –78 °C. The reaction mixture was stirred at –78 °C for 1 h and cooled to –94 °C. After stirring at –94 °C for 1 h, to the mixture was added MeI (2.5 M in THF solution, 60 μL, 0.13 mmol) in three portions at the same temperature. The reaction mixture was stirring at –94 °C for 2 h, to the mixture was added 1. 5 M solution of AcSK in methanol (0.4 mL, 0.560 mmol) at the same temperature for quenching excess MeI, and the mixture was warmed up to –78 °C. After stirring at –78 °C for 30 min, the reaction was quenched with saturated aqueous NH_4Cl at –78 °C and the mixture was extracted with AcOEt (x3). The combined organic layers were dried over $MgSO_4$, filtered, and concentrated under reduced pressure. The residue was purified by flash silica gel column chromatography (AcOEt/hexane = 1/4 to 1/0) to give **7** (52.8 mg, 85%) as a yellow oil. ^1H NMR (500 MHz, $CDCl_3$): δ 7.38-7.29 (m, 4H), 7.27 (m, 1H), 6.01 (d, *J* = 5.9 Hz, 1H), 5.88 (dd, *J* =5.9, 2.9 Hz, 1H), 4.46 (s, 2H), 4.30 (brd, *J* = 2.7 Hz, 1H), 3.79 (s, 3H), 3.78 (s, 1H), 3.54 (dd, *J* = 7.8, 5.9 Hz, 1H), 3.53 (t, *J* = 7.0 Hz, 1H), 3.50 (t, *J* = 8.6 Hz, 1H), 2.53 (s, 3H), 2.12 (dd, *J* = 14.1, 6.9 Hz, 1H), 1.98 (dt, *J* = 12.9, 6.7 Hz, 1H), 1.84 (dt, *J* = 11.3, 5.2 Hz, 1H), 1.80-1.64 (m, 3H), 1.62-1.42 (m, 2H), 1.20 (s, 3H); ^{13}C NMR (125 MHz, CD_3OD): δ 214.5, 171.9, 139.7, 139.6, 133.2, 129.4, 128.9, 128.7, 102.8, 82.8, 75.4, 74.0, 67.8, 66.2, 52.7, 51.8, 47.0, 37.6, 35.9, 29.0, 27.0, 22.7, 19.9; IR (KBr): 2949, 2867, 1739, 1436, 1223, 1098, 1053, 911, 732 cm^{-1}; HRMS-ESI (*m/z*): $[M+H]^+$ calcd for $C_{25}H_{32}NO_4S_2$, 474.1767; found, 474.1771; R_f = 0.7 (silica gel, MeOH/AcOEt = 1/10).

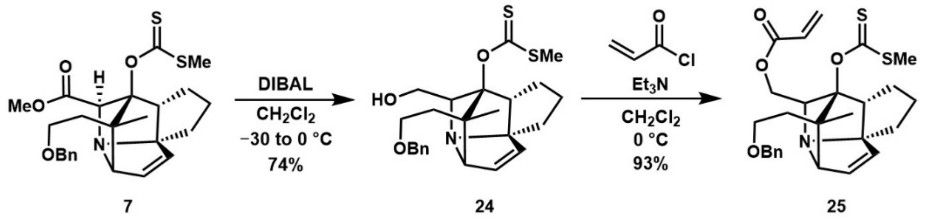

Compound **25**:

To a solution of **7** (52.8 mg, 0.111 mmol) in CH_2Cl_2 (1.11 mL) was added DIBAL (1.01 M solution in *n*-hexane, 254 μL, 0.256 mmol) at –30 °C, and the mixture was warmed up to 0 °C. After stirring at 0 °C for 30 min, the reaction was quenched with NH_4Cl and to the mixture was added saturated aqueous solution of potassium tartrate at 0 °C, and the mixture was warmed up to room temperature. After stirring at room temperature for 45 min, the mixture was extracted with AcOEt (x3). The combined organic layers were washed with brine, dried over $MgSO_4$, filtered, and concentrated under reduced pressure. The residue was purified by flash silica gel column chromatography (hexane/EtOAc = 1/4 to AcOEt/MeOH =9/1) to give **24** (37.3 mg, 74%) as a yellow oil.

To a solution of **24** (30.0 mg, 0.0673 mmol) in CH_2Cl_2 (0.673 mL) were added Et_3N (47.0 μL, 0.337 mmol) and acryloyl chloride (11.1 μL, 0.0234 mmol) at 0 °C. After stirring at 0 °C for 10 min, the reaction mixture was concentrated under reduced pressure. The residue was purified by flash silica gel column chromatography (AcOEt/hexane = 1/4 to AcOEt/MeOH =9/1) to give **25** (31.4 mg, 93%) as a colorless oil. ^{1}H NMR (500 MHz, $CDCl_3$): δ 7.34-7.29 (m, 4H), 7.26 (m, 1H), 6.44 (dd, *J* = 17.3, 1.4 Hz, 1H), 6.17 (dd, *J* = 17.3, 10.4 Hz, 1H), 5.99 (d, *J* = 5.8 Hz, 1H), 5.85 (dd, *J* = 5.9, 2.9 Hz, 1H), 5.83 (dd, *J* = 10.5, 1.4 Hz, 1H), 4.67 (d, *J* = 10.9 Hz, 1H), 4.52 (dd, *J* = 10.9, 9.1 Hz, 1H), 4.50 (d, *J* = 11.9 Hz, 1H), 4.44 (d, *J* = 11.9 Hz, 1H), 4.19 (d, *J* = 2.9 Hz, 1H), 3.63-3.52 (m, 2H), 3.42 (d, *J* = 8.9 Hz, 1H), 3.33 (t, *J* = 8.6 Hz, 1H), 2.49 (s, 3H), 2.11 (dd, *J* = 13.5, 6.8 Hz, 1H), 1.93-1.80 (m, 3H), 1.78-1.38 (m, 4H), 1.17 (s, 3H); ^{13}C NMR (125 MHz, CD_3OD): δ 215.2, 167.5, 139.8, 139.7, 133.4, 132.1, 129.4, 129.3, 128.9, 128.7, 101.7, 83.6, 74.2, 74.1, 68.1, 66.1, 63.4, 51.9, 46.8, 37.4, 36.2, 28.9, 27.2, 23.0, 20.2; IR (KBr): 2953, 2865, 1724, 1453, 1402, 1226, 1190, 1095, 1053, 750, 698 cm^{-1}; HRMS-ESI (*m/z*): [M+Na]$^+$ calcd for $C_{27}H_{33}NO_4S_2Na$, 522.1743; found, 522.1745; R$_f$ = 0.8 (silica gel, MeOH/AcOEt = 1/10).

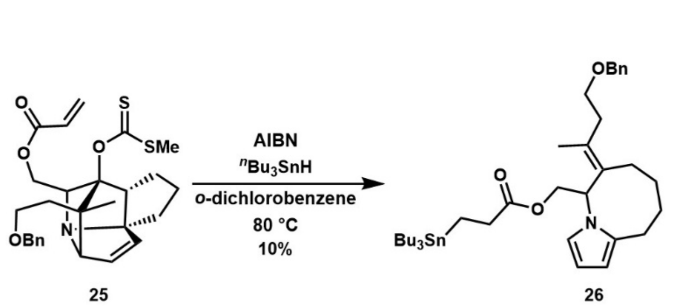

Compound **26**:

To a solution of **25** (14 mg, 0.028 mmol) in *o*-dichlorobenzene (1.40 mL) was degassed by freeze-thaw procedure (x3) and the mixture was warmed up to 80 °C. After stirring at 80 °C for 10 min, to the mixture was added a mixture of ^{n}Bu$_3$SnH (14.7 μL, 0.056 mmol) and AIBN (1.84 mg, 0.0112 mmol) in *o*-dichlorobenzene (200 μL) at 80 °C. After stirring at 80 °C for 2 h, the mixture was cooled to room temperature. The residue was purified by preparative TLC (AcOEt/hexane = 1/10) to afford **26** (2.0 mg, 10%) as a yellow oil. ^{1}H NMR (500 MHz, CDCl$_3$): δ 7.35-7.22 (m, 5H), 6.69 (t, *J* = 2.1 Hz, 1H), 6.05 (t, *J* = 3.0 Hz, 1H), 5.83 (brs, 1H), 5.32 (dd, *J* = 7.8, 4.6 Hz, 1H), 4.55-4.42 (m, 2H), 4.45 (s, 2H), 3.50 (t, *J* = 7.5 Hz, 2H), 2.72 (m, 1H), 2.65-2.56 (m, 2H), 2.41 (dd, *J* = 8.6, 8.5 Hz, 2H), 2.40-2.29 (m, 2H), 1.89 (m, 1H), 1.77 (s, 3H), 1.72 (m, 1H), 1.48 (m, 6H), 1.34 (m, 10H), 0.87 (t, *J* = 7.4 Hz, 11H), 0.81 (t, *J* = 8.3 Hz, 6H); ^{13}C NMR (125 MHz, CDCl$_3$): δ 175.5, 138.5, 135.3, 133.3, 131.7, 128.5, 127.7, 127.7, 120.5, 107.8, 106.9, 73.1, 68.4, 66.3, 58.6, 34.9, 31.6, 29.9, 29.3, 27.5, 26.1, 25.0, 20.0, 13.9, 9.0, 3.2, 1.2; IR (KBr): 2923, 2853, 1734, 1457, 1272, 1184, 1115, 1017, 738, 697 cm^{-1}; HRMS-ESI (*m/z*): [M+Na]$^+$ calcd for $C_{37}H_{59}NO_3SnNa$, 708.3409; found, 708.3406; R$_f$ = 0.8 (silica gel, AcOEt/hexane = 1/4).

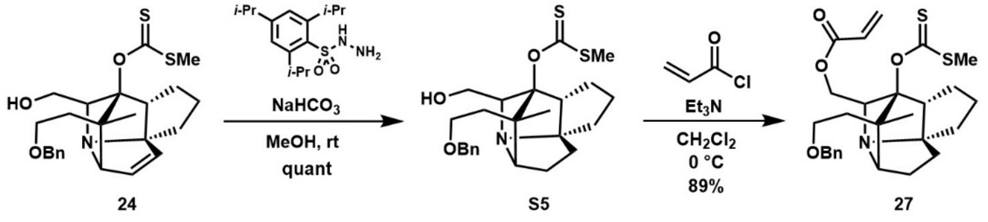

Compound **27**:

To a solution of **24** (37.3 mg, 0.0826 mmol) in anhydrous MeOH (8.26 mL) under an argon atmosphere were added 2,4,6-triisopropylbenzenesulfonyl hydrazide (123 mg, 0.413 mmol) and NaHCO$_3$ (34.7 mg, 0.413 mmol). After stirring for 2 hours at room temperature, the reaction was quenched by saturated aqueous NaHCO$_3$, and the mixture was extracted with Et$_2$O (x3). The combined organic layers were washed with brine, dried over MgSO$_4$, filtered, and concentrated under reduced pressure. The residue was purified by flash silica gel column chromatography (AcOEt/hexane = 1/1 to MeOH/AcOEt = 1/9) to give **S5** (50.4 mg, quant) as a colorless oil.

To a solution of **S5** (50.4 mg) in CH_2Cl_2 (0.826 mL) were added Et$_3$N (174 μL, 1.24 mmol) and acryloyl chloride (34.0 μL, 0.413 mmol) at 0 °C. After stirring at 0 °C for 10 min, the reaction mixture was concentrated under reduced pressure. The residue was purified by flash silica gel column chromatography (AcOEt/hexane = 1/4 to MeOH/AcOEt = 1/9) to give **27** (37.2 mg, 89%) as a colorless oil. ^{1}H NMR (500 MHz, CDCl$_3$): δ 7.36-7.29 (m, 4H), 7.26 (m, 1H), 6.46 (dd, *J* = 17.4, 1.4 Hz, 1H), 6.19 (dd, *J* = 17.4, 10.4 Hz, 1H), 5.84 (dd, *J* = 10.5, 1.5 Hz, 1H), 4.70 (dd, *J* = 11.2, 1.6 Hz,

1H), 4.51 (dd, J = 11.2, 9.3 Hz, 1H), 4.49 (d, J = 12.0 Hz, 1H), 4.44 (d, J = 11.9 Hz, 1H), 3.82 (brd, J = 5.7 Hz, 1H), 3.59-3.47 (m, 2H), 3.40 (d, J = 8.8 Hz, 1H), 3.14 (t, J = 8.4 Hz, 1H), 2.51 (s, 3H), 2.09-1.89 (m, 5H), 1.83-1.69 (m, 4H), 1.65 (dt, J = 14.7, 6.5 Hz, 1H), 1.56 (m, 1H), 1.45 (m, 1H), 1.31 (s, 3H); ^{13}C NMR (125 MHz, $CDCl_3$): δ 213.7, 166.3, 138.3, 131.3, 128.5, 128.3, 127.8, 127.7, 101.3, 79.7, 73.3, 70.5, 67.1, 65.9, 63.1, 63.1, 52.3, 44.8, 39.8, 37.7, 37.2, 29.8, 25.9, 21.1, 20.2; IR (KBr): 2955, 2870, 1724, 1454, 1402, 1226, 1197, 1094, 1059, 734, 698 cm^{-1}; HRMS-ESI (*m/z*): $[M+Na]^+$ calcd for $C_{27}H_{35}NO_4S_2Na$, 524.1900; found, 524.1905; R_f = 0.8 (silica gel, MeOH/AcOEt = 1/10).

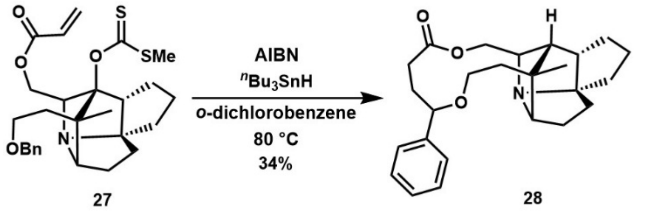

Compound **28**:

To a solution of **27** (17.0 mg, 0.0339 mmol) in *o*-dichlorobenzene (1.7 mL) was degassed by freeze-thaw procedure (x3) and the mixture was warmed up to 80 °C. After stirring at 80 °C for 10 min, to the mixture was added a mixture of ^{n}Bu$_3$SnH (17.8 μL, 0.0678 mmol) and AIBN (2.23 mg, 0.0136 mmol) in *o*-dichlorobenzene (200 μL) at 80 °C. After stirring at 80 °C for 2 h, the mixture was cooled to room temperature. The residue was purified by preparative TLC (MeOH/AcOEt = 1/9) to afford **28** (5.0 mg, 34%) as a yellow oil. ^{1}H NMR (500 MHz, $CDCl_3$): δ 7.37-7.24 (m, 5H), 4.73 (t, J = 11.8 Hz, 1H), 4.30 (dd, J = 8.5, 3.0 Hz, 1H), 4.08 (dd, J = 11.6, 7.4 Hz, 1H), 3.61 (td, J = 8.7, 5.1 Hz, 1H), 3.30-3.22 (m, 2H), 3.18 (d, J = 6.2 Hz, 1H), 2.46-2.33 (m, 2H), 2.35 (s, 1H), 2.15-1.98 (m, 2H), 2.00-1.88 (m, 3H), 1.88-1.72 (m, 3H), 1.69-1.55 (m, 3H), 1.45-1.21 (m, 3H), 0.91 (m, 1H), 0.82 (s, 3H); ^{13}C NMR (125 MHz, $CDCl_3$): δ 173.8, 141.9, 128.5, 127.6, 126.3, 83.4, 79.7, 71.8, 67.1, 64.3, 61.9, 51.2, 48.6, 44.1, 43.2, 39.2, 36.9, 34.7, 32.9, 31.9, 26.0, 24.7, 22.4; IR (KBr): 2916, 2848, 1733, 1262, 1150, 1092, 1025, 814, 733 cm^{-1}; HRMS-ESI (*m/z*): $[M+Na]^+$ calcd for $C_{25}H_{33}NO_3Na$, 418.2353; found, 418.2343; R_f = 0.2 (silica gel, MeOH/AcOEt = 1/10).

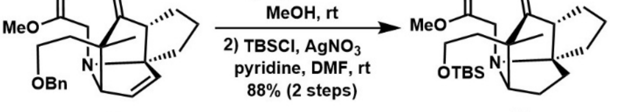

Compound **30**:

To a solution of **8** (49 mg, 0.128 mmol) in MeOH (2.55 mL) was added Pd/C (24 mg, 50 wt%) at room

temperature. The reaction mixture was stirred under hydrogen atmosphere at room temperature for 12 h, then flushed with argon, filtered, and concentrated under reduced pressure. The residue was used for next reaction without further purification.
To a solution of the residue in DMF (0.64 mL) were added $AgNO_3$ (109 mg, 0.64 mmol), pyridine (103 μL, 1.28 mmol) and TBSCl (38.6 mg, 0.256 mmol) at room temperature. After stirring at room temperature for 30 min, the reaction was quenched with saturated aqueous NH_4Cl at 0 °C and the mixture was extracted with Et_2O (x3). The combined organic layers were washed with brine, dried over $MgSO_4$, filtered, and concentrated under reduced pressure. The residue was purified by flash silica gel column chromatography (AcOEt/hexane = 1/40 to 1/10) to give **30** (46 mg, 88% for 2 steps) as a colorless oil. ^{1}H NMR (500 MHz, $CDCl_3$): δ 3.73 (s, 3H), 3.68 (ddd, J = 10.2, 9.0, 5.9 Hz, 1H), 3.51 (ddd, J = 10.3, 8.9, 5.9 Hz, 1H), 3.46 (d, J = 15.8 Hz, 1H), 3.14 (dd, J = 8.0, 1.6 Hz, 1H), 3.00 (d, J = 15.8 Hz, 1H), 2.37 (ddd, J = 14.9, 9.1, 6.0 Hz, 1H), 2.20 (t, J = 9.6 Hz, 1H), 2.05-1.52 (m, 11H), 1.01 (s, 3H), 0.87 (s, 9H), 0.032 (s, 3H), 0.026 (s, 3H); ^{13}C NMR (125 MHz, $CDCl_3$): δ 217.0, 172.4, 74.9, 70.8, 61.9, 60.0, 53.7, 51.9, 51.7, 41.3, 32.0, 32.0, 29.8, 26.1, 23.0, 23.0, 19.5, 18.4, -5.2; IR (KBr): 2954, 2881, 1758, 1700, 1471, 1255, 1196, 1091, 836, 775 cm^{-1}; HRMS-ESI (*m/z*): $[M+Na]^+$ calcd for $C_{22}H_{39}NO_4Na$, 432.2541; found, 432.2540; R_f = 0.5 (silica gel, AcOEt/hexane = 1/4).

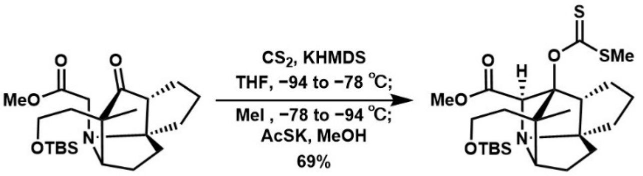

30 31

Compound **31**:
To a solution of **30** (46 mg, 0.112 mmol) in THF (1.12 mL) was added CS_2 (9.9 μL, 0.157 mmol) at room temperature, and the mixture was cooled to –94 °C. After stirring at –94 °C for 2 h, to the mixture was added KHMDS (1.0 M in THF solution, 134 μL, 0.134 mmol) at the same temperature and the mixture was warmed up to –78 °C. The reaction mixture was stirring at –78 °C for 1 h and cooled to –94 °C. After stirring at –94 °C for 1 h, to the mixture was added MeI (2 M in THF solution, 56 μL, 0.112 mmol) in three portions at the same temperature. The reaction mixture was stirring at –94 °C for 2 h and to the mixture was added AcSK (1.5 M in MeOH solution, 0.4 mL, 0.560 mmol) at the same temperature for quenching excess MeI and the mixture was warmed up to –78 °C. After stirring at –78 °C for 30 min, the reaction was quenched with saturated aqueous NH_4Cl at –78 °C and the mixture was extracted with AcOEt (x3). The combined organic layers were dried over $MgSO_4$, filtered, and concentrated under reduced pressure. The residue was purified by flash silica gel column chromatography (AcOEt/hexane = 1/8 to 2/1) to give **31** (38.5 mg, 69%) as a yellow oil. ^{1}H NMR

(500 MHz, $CDCl_3$): δ 4.03 (brd, *J* = 5.9 Hz, 1H), 3.79 (s, 3H), 3.73 (s, 1H), 3.66 (ddd, *J* = 10.4, 7.8, 5.5 Hz, 1H), 3.62 (ddd, *J* = 14.2, 10.3, 7.2 Hz, 1H), 3.31 (t, *J* = 8.2 Hz, 1H), 2.55 (s, 3H), 2.09-1.94 (m, 5H), 1.84-1.65 (m, 3H), 1.59 (ddd, *J* = 14.0, 7.3, 5.5 Hz, 1H), 1.56-1.39 (m, 3H), 1.33 (s, 3H), 0.86 (s, 9H), 0.017 (s, 3H), 0.014 (s, 3H); ^{13}C NMR (125 MHz, $CDCl_3$): δ 212.9, 171.0, 102.9, 79.1, 72.0, 66.5, 59.8, 52.2, 45.1, 40.2, 40.0, 37.2, 30.1, 26.0, 25.8, 25.7, 20.7, 19.7, 19.7, 18.3, -5.3; IR (KBr): 2952, 2856, 1744, 1471, 1225, 1100, 1023, 836, 776cm^{-1}; HRMS-ESI (*m/z*): $[M+H]^+$ calcd for $C_{24}H_{42}NO_4SiS_2$, 500.2319 found, 500.2321; R_f = 0.8 (silica gel, MeOH/AcOEt = 1/10).

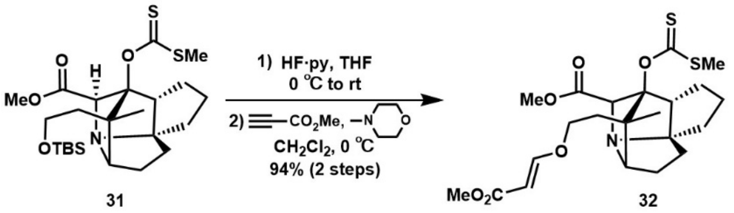

Compound **32**:

To a solution of **31** (54 mg, 0.108 mmol) in THF (1.08 mL) was added dropwise HF·py (0.216 mL) at 0 °C, and the mixture was stirred at the same temperature for 5 min. The mixture was further stirred at room temperature for 2 h. The reaction was quenched with TMSOMe (3.0 mL) at 0 °C, and the mixture was stirred at 0 °C for 5 min followed by room temperature for 30 min and concentrated under reduced pressure. The residue was used for the next reaction without further purification.

To a solution of the residue in CH_2Cl_2 (1.08 mL) were added methyl propiolate (35.6 μL, 0.432 mmol) and *N*-methyl morpholine (47.5 μL, 0.432 mmol) at 0 °C. After stirring at 0 °C for 4 h, the reaction was quenched with H_2O and the mixture was extracted with AcOEt (x3). The combined organic layers were dried over $MgSO_4$, filtered, and concentrated under reduced pressure. The residue was purified by flash silica gel column chromatography (AcOEt/hexane = 1/2 to AcOEt/MeOH = 9/1) to give **32** (47.7 mg, 94% for 2 steps) as a yellow oil. ^{1}H NMR (500 MHz, $CDCl_3$): δ 7.51 (d, *J* = 12.7 Hz, 1H), 5.17 (d, *J* = 12.7 Hz, 1H), 3.92-3.77 (m, 3H), 3.80 (s, 3H), 3.75 (s, 1H), 3.68 (s, 3H), 3.28 (t, *J* = 8.1 Hz, 1H), 2.55 (s, 3H), 2.12-1.92 (m, 5H), 1.84-1.62 (m, 5H), 1.60-1.38 (m, 2H) 1.33 (s, 3H); ^{13}C NMR (125 MHz, $CDCl_3$): δ 213.1, 171.1, 168.3, 162.3, 102.6, 96.5, 79.2, 72.3, 67.8, 66.6, 52.4, 52.3, 51.2, 44.9, 39.9, 37.2, 37.1, 30.0, 25.7, 25.6, 21.0, 19.8; IR (KBr): 2951, 1738, 1712, 1625, 1455, 1207, 1137, 1023, 754 cm^{-1}; HRMS-ESI (*m/z*): $[M+Na]^+$ calcd for $C_{22}H_{31}O_6S_2Na$, 492.1485; found, 492.1487; R_f = 0.6 (silica gel, MeOH/AcOEt = 1/10).

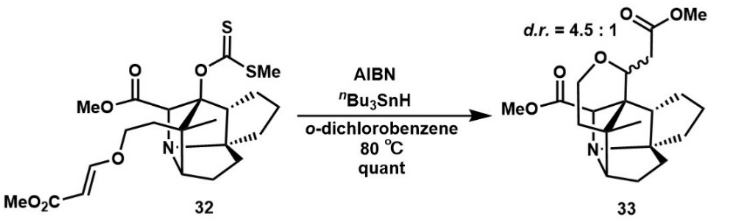

Compound **33**:

To a solution of **32** (47.7 mg, 0.108 mmol) in *o*-dichlorobenzene (4.40 mL) was degassed by freeze-thaw procedure (x3) and the mixture was warmed up to 80 °C. After stirring at 80 °C for 10 min, the mixture was added a mixture of $^{n}Bu_3SnH$ (56.6 µL, 0.216 mmol) and AIBN (7.1 mg, 4.32 µmol) in *o*-dichlorobenzene (200 µL) at 80 °C. After stirring at 80 °C for 2 h, the mixture was cooled to room temperature. The residue was purified by flash silica gel column chromatography (AcOEt/hexane = 1/2 to AcOEt/MeOH = 3/1) to give **33** (39.2 mg, quant) as a yellow oil. ^{1}H NMR (500 MHz, $CDCl_3$): δ 4.71 (dd, *J* = 11.8, 2.6 Hz, 0.22H), 4.23 (dd, *J* = 10.7, 3.0 Hz, 1H), 3.80-3.67 (m, 2.44H), 3.77 (s, 3H), 3.75 (s, 0.66H), 3.71 (s, 0.66H), 3.70 (s, 3H), 3.55 (s, 1H), 3.56-3.46 (m, 1.44H), 3.06 (m, 0.22H), 3.04 (dd, *J* = 14.6, 10.7 Hz, 1H), 2.69 (dd, *J* = 14.7, 3.0 Hz, 1H), 2.35 (dd, *J* = 14.7, 2.9 Hz, 0.22H), 2.17-1.53 (m, 12.2H), 1.30-1.20 (m, 3.66H), 1.11 (s, 0.66H), 1.08 (s, 3H); ^{13}C NMR (125 MHz, $CDCl_3$): δ 172.5, 171.9, 77.8, 73.1, 71.9, 64.0, 63.8, 59.6, 54.1, 52.1, 51.7, 41.4, 39.2, 38.6, 38.4, 37.1, 32.5, 25.8, 24.6, 18.2; IR (KBr): 2952, 2871, 1740, 1454, 1208, 1072, 727 cm^{-1}; HRMS-ESI (*m/z*): $[M+Na]^+$ calcd for $C_{20}H_{29}NO_5Na$,386.1938; found, 386.1953; R_f = 0.4 (silica gel, MeOH/AcOEt = 1/10).

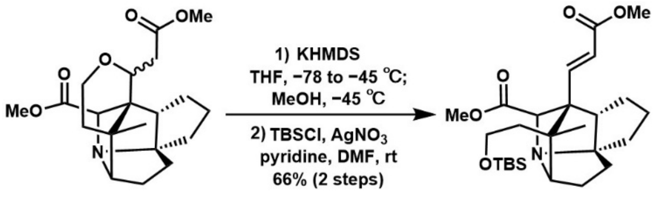

Compound **34**:

To a solution of **33** (39.2 mg, 0.108 mmol) in THF (1.08 mL) was added KHMDS (1.0 M in THF solution, 324 µL, 0.324 mmol) at -78 °C. The mixture was stirred at -78 °C for 30 min and warmed up to -45 °C. After stirring at -45 °C for 2 h, to the mixture was added MeOH and the mixture was stirred at -45 °C for 15 min. Then, the reaction was quenched with saturated aqueous NH_4Cl solution and the mixture was extracted with AcOEt (x3). The combined organic layers were dried over $MgSO_4$, filtered, and concentrated under reduced pressure. The residue was used for the next reaction without further purification.

To a solution of the residue in DMF (0.54 mL) were added $AgNO_3$ (91.7 mg, 0.54 mmol), pyridine

(87.2 μL, 1.08 mmol) and TBSCl (48.8 mg, 0.324 mmol) at room temperature. After stirring at room temperature for 30 min, the reaction was quenched with H_2O at 0 °C and the mixture was extracted with Et_2O (x3). The combined organic layers were washed with brine, dried over $MgSO_4$, filtered, and concentrated under reduced pressure. The residue was purified by flash silica gel column chromatography (hexane/EtOAc = 1/4 to AcOEt/MeOH = 9/1) to give **34** (34 mg, 66% for 2 steps) as a yellow oil. ^{1}H NMR (500 MHz, $CDCl_3$): δ 7.31 (d, J = 16.4 Hz, 1H), 5.94 (d, J = 16.4 Hz, 1H), 3.85 (brd, J = 6.5 Hz, 1H), 3.76 (s, 3H), 3.74 (s, 3H), 3.62-3.51 (m, 2H), 3.52 (s, 1H), 2.14-1.80 (m, 6H), 1.72-1.52 (m, 3H), 1.40-1.20 (m, 3H), 1.11 (m, 1H), 0.88 (s, 3H), 0.85 (s, 9H), -0.0038 (s, 6H); ^{13}C NMR (125 MHz, $CDCl_3$): δ 172.2, 166.8, 145.7, 123.6, 77.1, 68.9, 66.6, 64.9, 59.9, 54.0, 52.2, 51.7, 47.8, 41.2, 39.4, 37.1, 31.5, 26.0, 25.5, 25.2, 18.3, 16.7, -5.4; IR (KBr): 2935, 2875, 1744, 1645, 1435, 1258, 1198, 1172, 1092, 835, 776 cm^{-1}; HRMS-ESI (*m/z*): $[M+H]^+$ calcd for $C_{26}H_{44}NO_5Si$, 478.2983; found, 478.2996; R_f = 0.7 (silica gel, MeOH/AcOEt = 1/9).

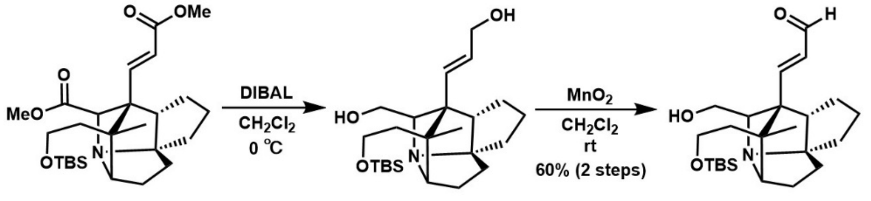

34 S6 35

Compound **35**:

To a solution of **34** (45.0 mg, 94.3 μmol) in CH_2Cl_2 (943 μL) was added DIBAL (1.0 M solution in *n*-hexane, 56.7 mL, 0.567 mmol) at 0 °C. After stirring at 0 °C for 30 min, the reaction was quenched with saturated aqueous NH_4Cl solution and to the mixture was added saturated aqueous solution of potassium tartrate at 0 °C, and the mixture was warmed up to room temperature. After stirring at room temperature for 45 min, the mixture was extracted with AcOEt (x3). The combined organic layers were washed with brine, dried over $MgSO_4$, filtered, and concentrated under reduced pressure to give crude **S6** as a colorless oil. The crude **S6** was used for next reaction without further purification.

To a solution of crude **S6** in CH_2Cl_2 (0.943 mL) was added MnO_2 (154 mg, 1.89 mmol) at room temperature. After stirring at room temperature for 4 h, the reaction mixture was filtered and concentrated under reduced pressure. The residue was purified by flash silica gel column chromatography (hexane/EtOAc = 1/2 to AcOEt/MeOH = 4/1) to give **35** (24 mg, 60% for 2 steps) as a yellow oil. ^{1}H NMR (500 MHz, $CDCl_3$): δ 9.51 (d, J = 7.6 Hz, 1H), 6.80 (d, J = 16.2 Hz, 1H), 6.16 (dd, J = 16.2, 7.6 Hz, 1H), 4.01 (dd, J = 11.6, 8.9 Hz, 1H), 3.91 (brd, J = 5.8 Hz, 1H), 3.81 (dd, J = 11.6, 1.6 Hz, 1H), 3.73-3.62 (m, 2H), 3.19 (d, J = 8.0 Hz, 1H), 2.18-1.91 (m, 5H), 1.80-1.65 (m, 4H), 1.48 (dt, J = 14.2, 6.6 Hz, 1H), 1.36 (m, 1H), 1.28 (dt, J = 14.3, 5.9 Hz, 1H), 1.14 (m, 1H), 0.97 (s, 3H), 0.88 (s, 9H), 0.036 (s, 6H); ^{13}C NMR (125 MHz, $CDCl_3$): δ 193.4, 154.7, 134.4, 77.5, 70.3, 67.6,

62.5, 60.6, 59.8, 55.2, 46.2, 41.6, 39.0, 37.5, 30.8, 26.0, 25.6, 25.1, 18.3, 18.1, -5.3; IR (KBr): 2952, 2857, 1689, 1471, 1255, 1091, 835, 777 cm^{-1}; HRMS-ESI (*m/z*): $[M+H]^+$ calcd for $C_{24}H_{42}NO_3Si$, 420.2928; found, 420.2927; R_f = 0.7 (silica gel, MeOH/AcOEt = 1/2).

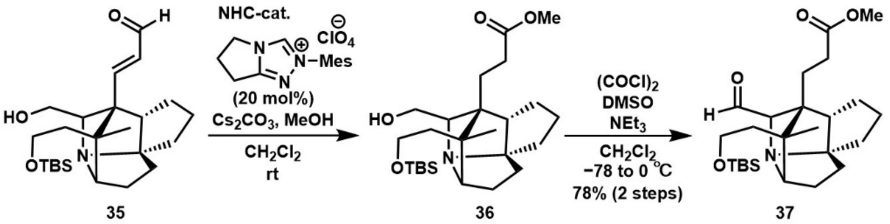

35 36 37

Compound **37**:

To a solution of **35** (29.0 mg, 69.1 μmol) in CH_2Cl_2 (1.38 mL) were added NHC-cat. (4.53 mg, 13.8 μmol), Cs_2CO_3 (13.5 mg, 41.4 μmol) and MeOH (28.7 μL, 0.691 mmol) at room temperature. After stirring at room temperature for 1 h, the reaction was quenched with saturated aqueous NH_4Cl and the mixture was extracted with AcOEt (x3). The combined organic layers were dried over $MgSO_4$, filtered, and concentrated under reduced pressure. The residue was used for the next reaction without further purification.

To a solution of DMSO (37.2 μL, 0.518 mmol) in CH_2Cl_2 (800 μL) was added oxalyl chloride (30.0 μL, 0.346 mmol) at –78 °C. After stirring at –78 °C for 30 min, to the mixture was added a solution of **36** in CH_2Cl_2 (538 μL) at –78 °C. After the mixture was stirred at –78 °C for 30 min, to the mixture was added NEt_3 (145 μL, 1.04 mmol) and the reaction mixture was warmed up to 0 °C. After stirring at 0 °C for 1 h, the reaction was quenched with saturated aqueous $NaHCO_3$ at 0 °C and the mixture was extracted with EtOAc (x6). The combined organic layers were washed with brine, dried over $MgSO_4$, filtered, and concentrated under reduced pressure. The residue was purified by flash silica gel column chromatography (hexane/EtOAc = 1/2 to AcOEt/MeOH = 4/1) to give **37** (24.3mg, 78% for 2 steps) as a colorless oil. ^{1}H NMR (500 MHz, $CDCl_3$): δ 10.0 (d, *J* = 1.0 Hz, 1H), 3.80 (d, *J* = 6.1 Hz, 1H), 3.73-3.61 (m, 2H), 3.67 (s, 3H), 3.06 (s, 1H), 2.47 (ddd, *J* = 15.2, 13.0, 5.6 Hz, 1H), 2.23 (ddd, *J* = 15.3, 12.1, 3.9 Hz, 1H), 2.16-1.80 (m, 9H), 1.80-1.65 (m, 2H), 1.58 (dt, *J* =13.9, 6.2 Hz, 1H), 1.54 (m, 1H) 1.42-1.25 (m, 2H), 0.97 (s, 3H), 0.87 (s, 9H), 0.029 (s, 6H); ^{13}C NMR (125 MHz, $CDCl_3$): δ 201.8, 173.6, 78.5, 75.2, 70.1, 61.9, 59.9, 54.1, 51.9, 43.9, 41.8, 39.3, 37.0, 30.9, 29.4, 26.0, 25.6, 25.6, 21.1, 18.8, 18.3, -5.4; IR (KBr): 2952, 2857, 1738, 1471, 1256, 1090, 835, 776 cm^{-1}; HRMS-ESI (*m/z*): $[M+Na]^+$ calcd for $C_{25}H_{43}NO_4SiNa$, 472.2854; found, 472.2857; R_f = 0.5 (silica gel, MeOH/AcOEt = 1/9).

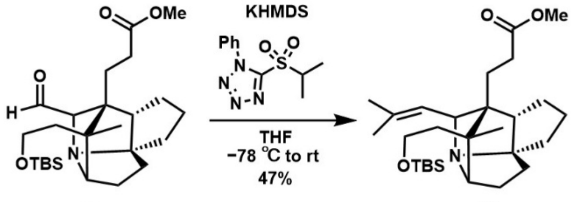
37 38

Compound **38**:

To a solution of PT-sulfone (23.6 mg, 93.4 μmol) in THF (400 μL) was added KHMDS (1.0 M solution in THF, 93.4 μL, 93.4 μmol) at −78 °C. After stirring at −78 °C for 30 min, to the mixture was added a solution of **37** (14 mg, 31 μmol) in THF (220 μL) at −78 °C. After the mixture was stirred at −78 °C for 1 h, the reaction mixture was slowly warmed up to room temperature. After stirring at room temperature for 12 h, the reaction was quenched with saturated aqueous NH_4Cl at 0 °C and the mixture was extracted with EtOAc (x3). The combined organic layers were dried over $MgSO_4$, filtered, and concentrated under reduced pressure. The residue was purified by flash silica gel column chromatography (hexane/EtOAc = 1/2 to AcOEt/MeOH = 4/1) to give **38** (7.0 mg, 47%) as a colorless oil. ^{1}H NMR (500 MHz, C_6D_6): δ 5.64 (d, J = 7.9 Hz, 1H), 4.05 (brs, 1H), 3.68 (td, J = 6.6, 2.9 Hz, 2H), 3.58 (d, J = 7.6 Hz, 1H), 3.37 (s, 3H), 2.36 (td, J = 10.1, 2.3 Hz, 1H), 2.29 (dt, J = 13.9, 6.9 Hz, 1H), 2.18-1.74 (m, 6H), 1.65-1.42 (m, 5H), 1.67 (s, 3H), 1.59 (s, 3H), 1.40-1.19 (m, 3H), 1.13 (m, 1H), 1.01 (s, 9H), 0.67 (s, 3H), 0.11 (s, 6H); ^{13}C NMR (125 MHz, CD_3OD); δ 175.3, 139.0, 120.8, 80.4, 70.1, 66.2, 61.4, 59.8, 54.5, 52.3, 45.3, 41.3, 39.8, 37.7, 31.2, 30.8, 30.3, 26.6, 26.4, 26.4, 25.9, 22.6, 20.2, 19.1, -5.3; IR (KBr): 2953, 2856, 1738, 1470, 1257, 1090, 835 cm^{-1}; HRMS-ESI (*m/z*): [M+H]$^+$ calcd for $C_{28}H_{50}NO_3Si$, 476.3554; found, 476.3552; R$_f$ = 0.2 (silica gel, MeOH/AcOEt = 1/9).

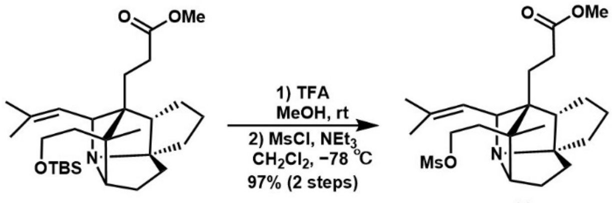
38 39

Compound **39**:

To a solution of **38** (6.0 mg, 12.6 μmol) in MeOH (252 μL) was added TFA (9.65 μL, 0.126 mmol) at 0 °C. Then the reaction mixture was warmed up to room temperature. After stirring at room temperature for 12 h, the mixture was concentrated under reduced pressure. The residue was used for the next reaction without further purification.

To a solution of the residue in CH_2Cl_2 (252 μL) were added NEt_3 (10.5 μL, 75.6 μmol) and MsCl (2.91 μL, 37.8 μmol) at −78 °C. After stirring at −78 °C for 40 min, the reaction was quenched with saturated aqueous $NaHCO_3$ at −78 °C and the mixture was extracted with EtOAc (x6). The combined organic

layers were dried over $MgSO_4$, filtered, and concentrated under reduced pressure. The residue was purified by short pass flash silica gel column chromatography (EtOAc/MeOH = 10/1 to 4/1) to give **39** (5.4 mg, 97% for 2 steps) as a colorless oil. **39** was needed to immediately use for the next step due to its instability. ^{1}H NMR (500 MHz, $CDCl_3$): δ 5.42 (d, *J* = 8.2 Hz, 1H), 4.49 (d, *J* = 6.4 Hz, 1H), 4,41-4.26 (m, 2H), 4.08 (d, *J* = 8.3 Hz, 1H), 3.69 (s, 3H), 3.11 (s, 3H), 2.60-1.47 (m, 17H), 1.82 (s, 3H), 1.71 (s, 3H), 1.10 (s, 3H); R_f = 0.3 (silica gel, MeOH/AcOEt = 1/2).

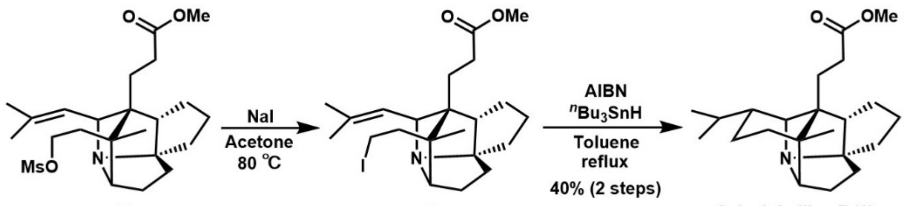

Calyciphylline F (**1**):

To a solution of **39** (5.4 mg, 12.3 μmol) in acetone (246 μL) was added NaI (2.74 mg, 18.5 μmol) at room temperature. Then the reaction mixture was heated to 80 °C. After stirring at 80 °C for 1 h, the reaction mixture was cooled to room temperature and the reaction was quenched with saturated aqueous $NaHCO_3$, and the mixture was extracted with EtOAc (x6). The combined organic layers were dried over $MgSO_4$, filtered, and concentrated under reduced pressure. The residue was used for the next reaction as soon as possible without further purification. The crude **6**: ^{1}H NMR (500 MHz, CD_3OD): δ 5.38 (d, *J* = 8.6 Hz, 1H), 3.94 (brd, *J* = 5.3 Hz, 1H), 3.82 (d, *J* = 8.4 Hz, 1H), 3.68 (s, 3H), 3.35-3.21 (m, 2H), 2.62-2.48 (m, 2H), 2.36-2.24 (m, 2H), 2.23-1.46 (m, 13H), 1.85 (s, 3H), 1.75 (s, 3H), 1.03 (s, 3H); R_f = 0.5 (silica gel, MeOH/AcOEt = 1/2).

To a solution of **6** in toluene (615 μL) were added ^{n}Bu$_3$SnH (6.45 μL, 24.6 μmol) and AIBN (0.403 mg, 24.6 μmol) at room temperature. The reaction mixture was degassed by freeze-thaw procedure (x3), heated to 110 °C. After stirring at 110 °C for 1 h, the reaction mixture was cooled to room temperature and concentrated under reduced pressure. The residue was purified by preparative TLC (amine-coated silica gel 60 F_{254} Plate-*Wako*) (hexane/EtOAc = 1/2) and azeotropic treatment with 0.1% TFA aq. provided pure Calyciphylline F (**1**) (1.7 mg, 40% for 2 steps) as a white solid. ^{1}H NMR (500 MHz, CD_3OD): δ 4.18 (t, *J* = 5.3 Hz, 1H), 3.72 (s, 3H), 3.66 (d, *J* = 2.2 Hz, 1H), 2.57 (ddd, *J* = 16.5, 12.0, 5.9 Hz, 1H), 2.44 (dd, *J* = 8.6, 8.4 Hz, 1H), 2.38 (ddd, *J* = 15.9, 11.4, 4.4 Hz, 1H), 2.33 (m, 1H), 2.23-2.14 (m, 3H), 2.15-1.78 (m, 7H), 1.83 (ddd, *J* = 16.1, 12.1, 4.4 Hz, 1H), 1.74-1.60 (m, 2H), 1.60-1.39 (m, 4H), 1.11 (s, 3H), 1.07 (d, *J* = 6.7 Hz, 3H), 1.05 (d, *J* = 6.5 Hz, 3H); ^{13}C NMR (125 MHz, CD_3OD): δ 175.9, 85.3, 70.6, 69.7, 53.3, 53.1, 52.4, 44.7, 39.8, 37.6, 35.1, 35.0, 34.1, 31.4, 30.2, 27.5, 25.0, 23.9, 22.0, 21.8, 21.6, 19.6; IR (KBr): 2956, 2873, 1741, 1175 cm^{-1}; HRMS-ESI (*m/z*): $[M+H]^+$ calcd for $C_{22}H_{36}NO_2$, 346.2741; found, 346.2746; R_f = 0.2 (silica gel, MeOH/AcOEt = 1/1).

^{1}H-NMR Comparison of Synthetic and Natural Calyciphylline F

Natural

Position		δH
1	3.65	(1H, d, 1.8)
2	1.53	(1H, m)
3a	1.99	(1H, m)
3b	1.5	(1H, m)
4a	1.97	(1H, m)
4b	1.58	(1H, m)
5	-	
6	4.18	(1H, t, 5.2)
7	-	
8	-	
9	2.42	(1H, m)
10	-	
11a	2.31	(1H, m)
11b	2.08	(1H, m)
12	2.18	(2H, m)
13a	2.12	(1H, m)
13b	1.82	(1H, ddd, 16.4, 11.8, 4.4)
14a	2.56	(1H, ddd, 16.4, 11.8, 5.8)
14b	2.38	(1H, m)
15a	2.05	(1H, m)
15b	1.66	(1H, m)
16a	2.02	(1H, m)
16b	1.66	(1H, m)
17a	2.17	(1H, m)
17b	1.89	(1H, m)
18	1.52	(1H, m)
19	1.06	(3H, d, 6.0)
20	1.04	(3H, d, 6.0)
21	1.1	(3H, s)
22	-	
23	3.72	(3H, s)

Synthetic

Position		δH
1	3.66	(1H, d, 2.2)
2	1.53	(1H, m)
3a	1.99	(1H, m)
3b	1.49	(1H, m)
4a	2.03-1.93	(1H, m)
4b	1.79-1.60	(1H, m)
5	-	
6	4.18	(1H, t, 5.2)
7	-	
8	-	
9	2.44	(1H, m)
10	-	
11a	2.31	(1H, m)
11b	2.08	(1H, m)
12	2.19	(2H, m)
13a	2.12	(1H, m)
13b	1.83	(1H, ddd, 16.2, 12.0, 4.4)
14a	2.57	(1H, ddd, 16.5, 12.0, 5.9)
14b	2.39	(1H, m)
15a	2.08	(1H, m)
15b	1.68	(1H, m)
16a	2.02	(1H, m)
16b	1.79-1.60	(1H, m)
17a	2.18	(1H, m)
17b	1.91	(1H, m)
18	1.53	(1H, m)
19	1.07	(3H, d, 6.7)
20	1.05	(3H, d, 6.5)
21	1.11	(3H, s)
22	-	
23	3.72	(3H, s)

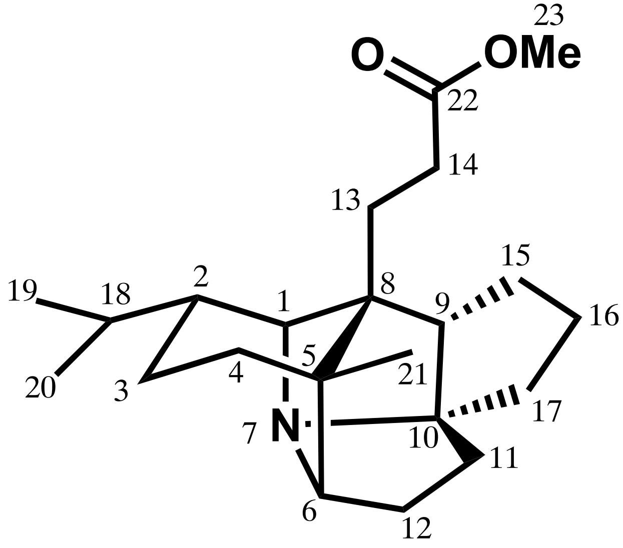

Calyciphylline F

1

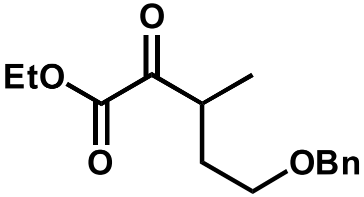

12

^{1}H, 500 MHz, $CDCl_3$

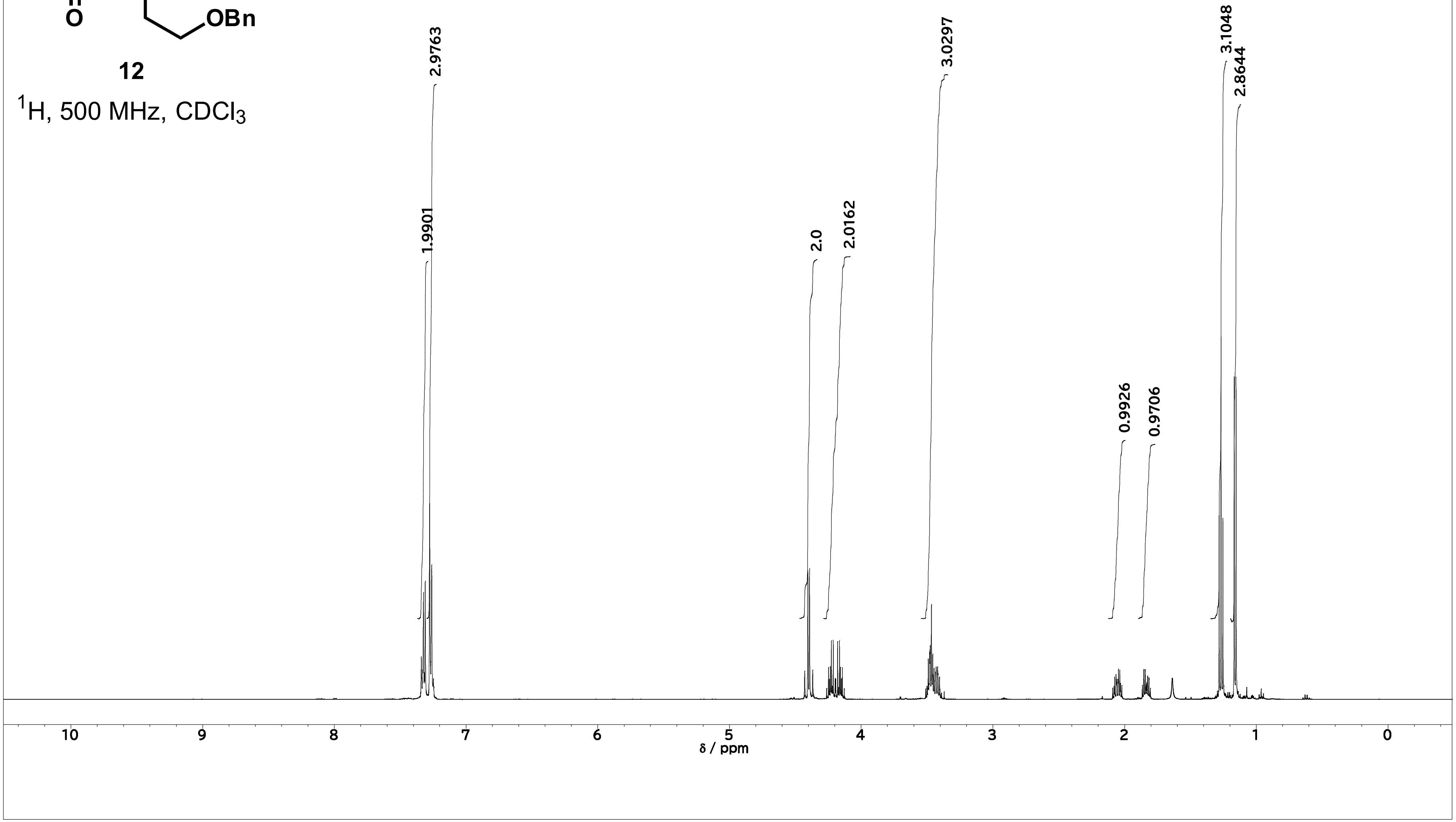

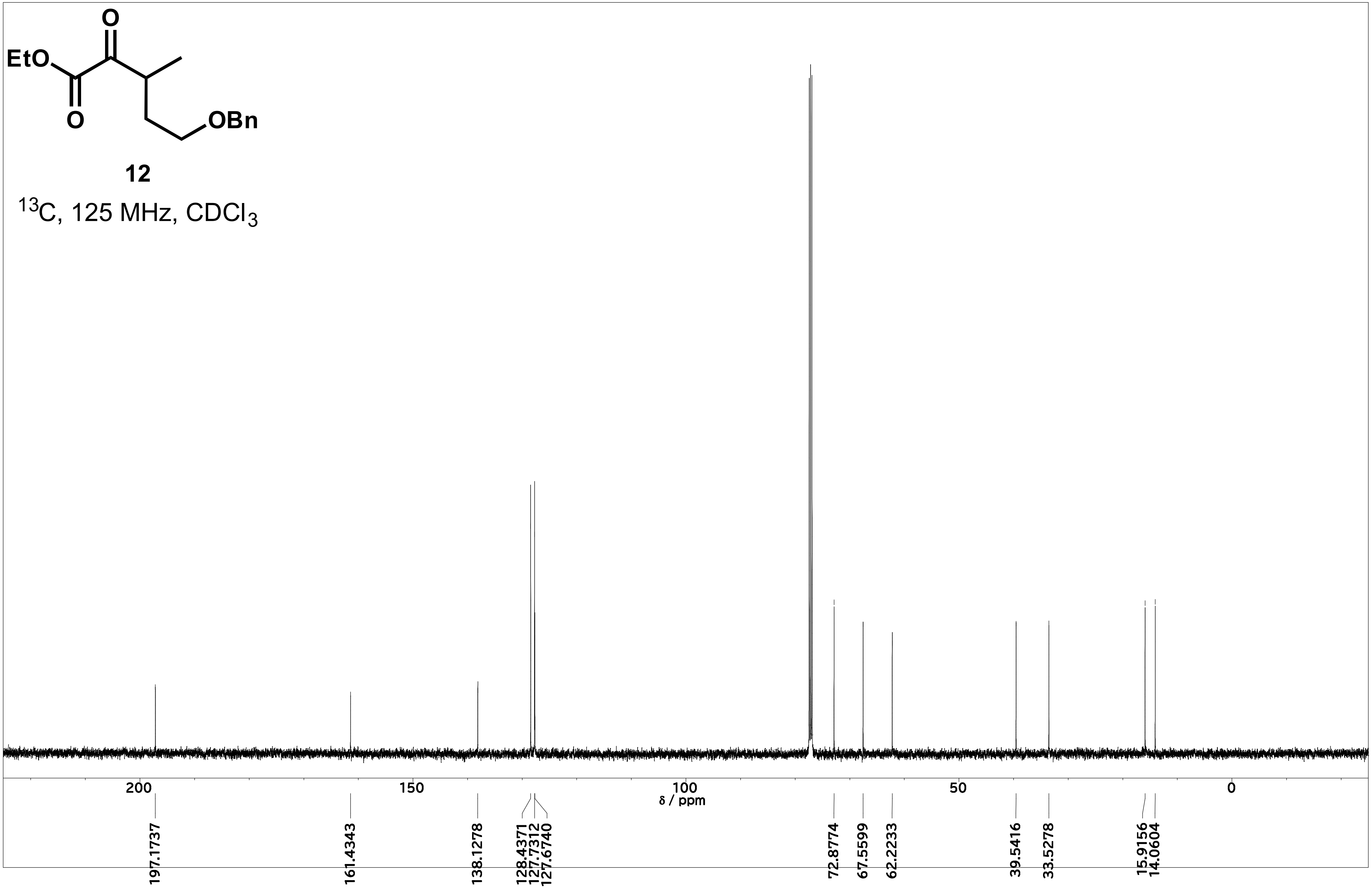
EtO
O
O
OBn
12
^{13}C, 125 MHz, $CDCl_3$
200
150
100
50
0
δ / ppm
197.1737
161.4343
138.1278
128.4371
127.7312
127.6740
72.8774
67.5599
62.2233
39.5416
33.5278
15.9156
14.0604

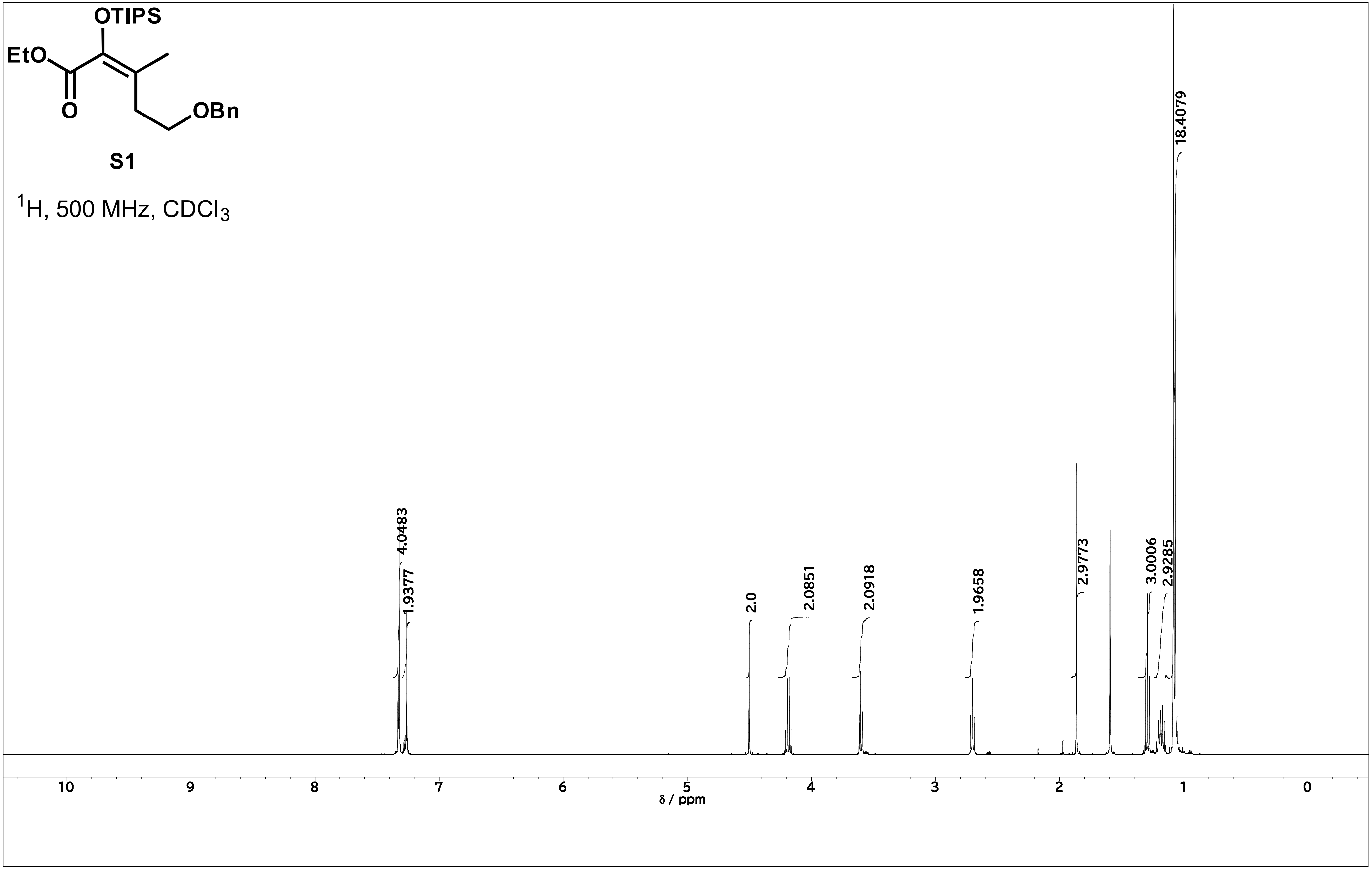
OTIPS
EtO
O
OBn
S1
^{1}H, 500 MHz, CDCl$_3$
4.0483
1.9377
2.0
2.0851
2.0918
1.9658
2.9773
3.0006
2.9285
18.4079
10
9
8
7
6
5
4
3
2
1
0
δ / ppm

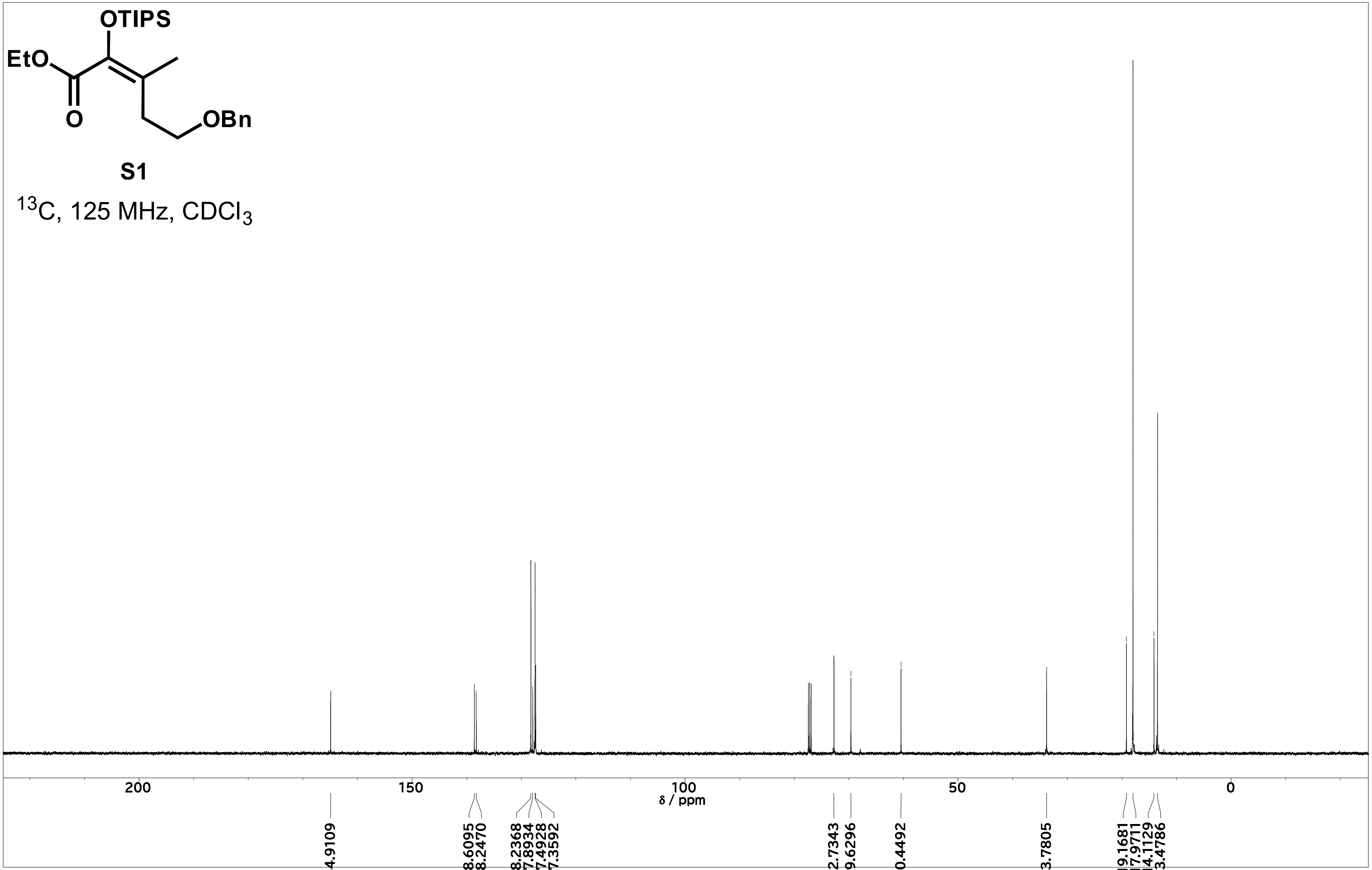
OTIPS
EtO
O
OBn
S1
^{13}C, 125 MHz, CDCl$_3$
200
150
100
50
0
δ / ppm
164.9109
138.6095
138.2470
128.2368
127.8934
127.4928
127.3592
72.7343
69.6296
60.4492
33.7805
19.1681
17.9711
14.1129
13.4786

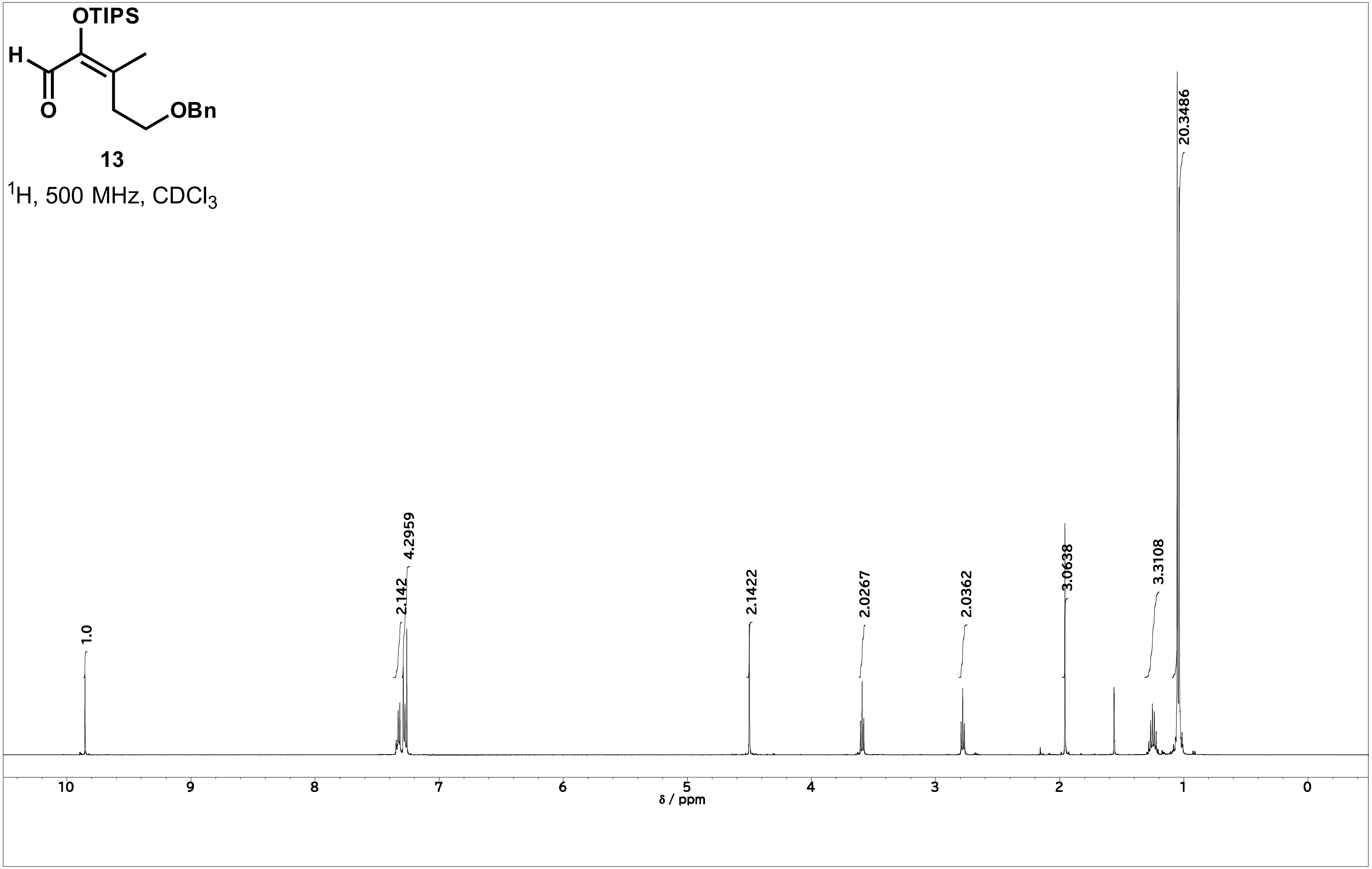
OTIPS
H
O
OBn
13
^{1}H, 500 MHz, $CDCl_3$
1.0
2.142
4.2959
2.1422
2.0267
2.0362
3.0638
3.3108
20.3486
10
9
8
7
6
5
4
3
2
1
0
δ / ppm

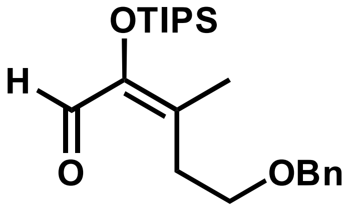

13

^{13}C, 125 MHz, $CDCl_3$

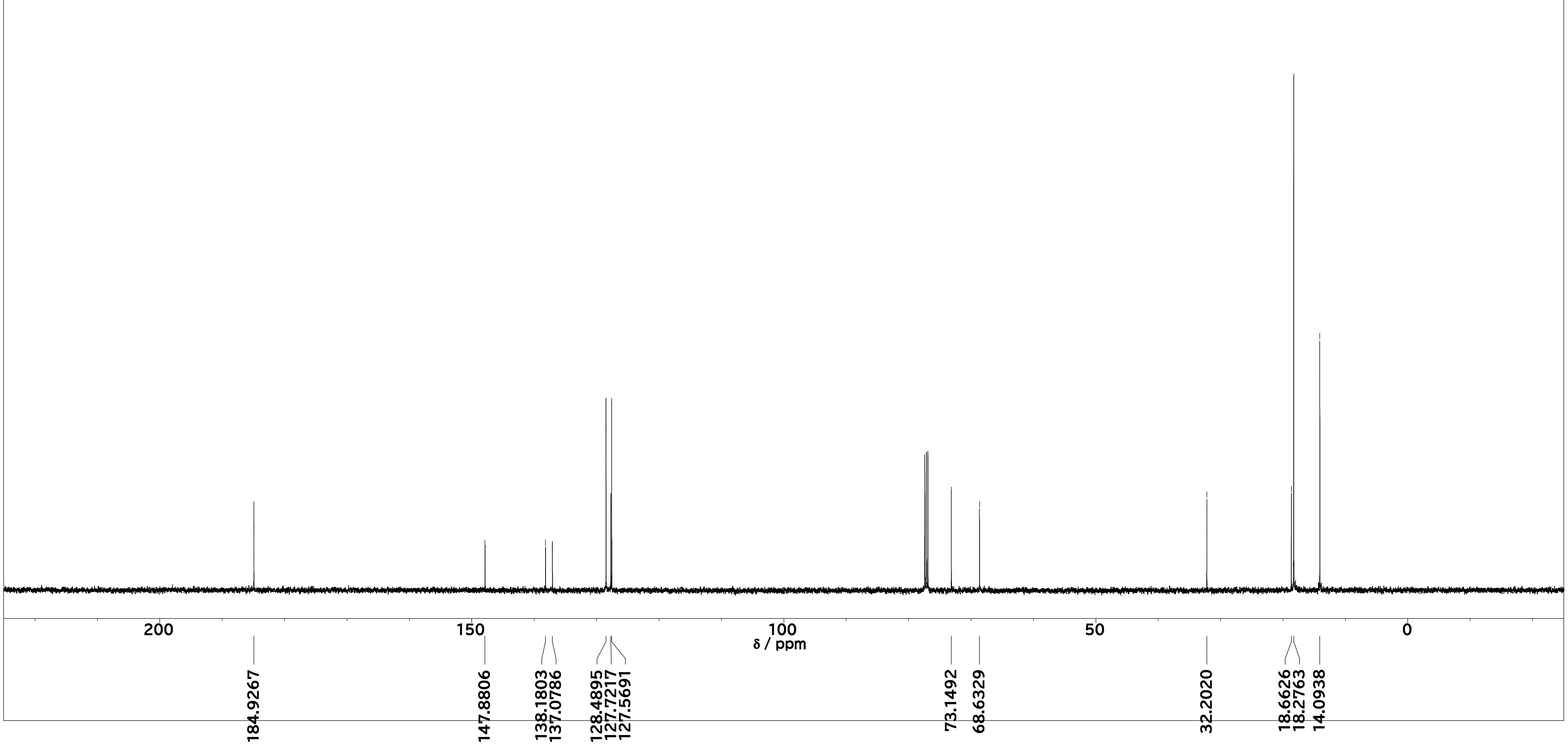

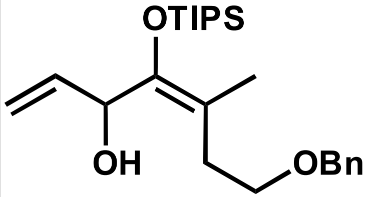

14

^{1}H, 500 MHz, $CDCl_3$

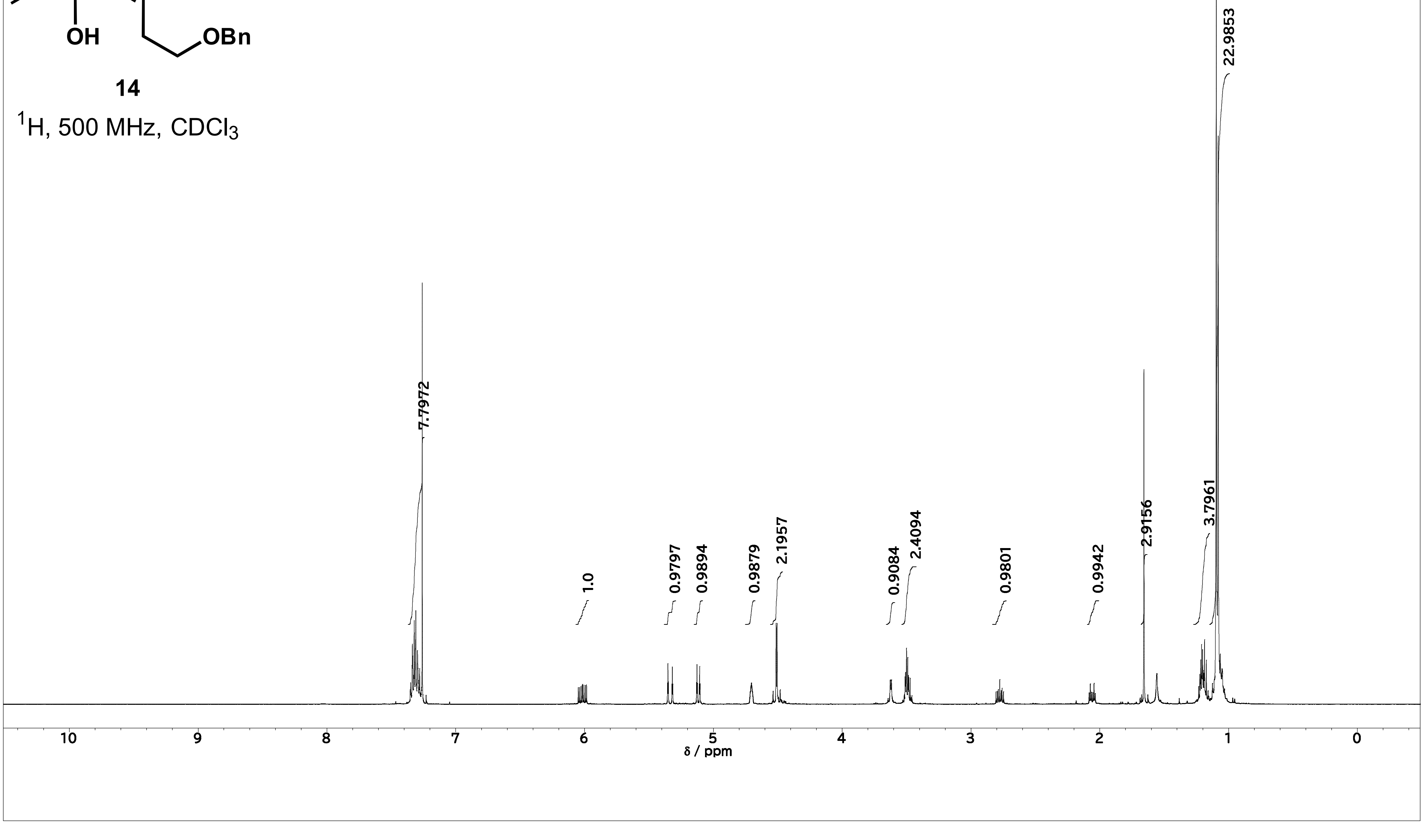

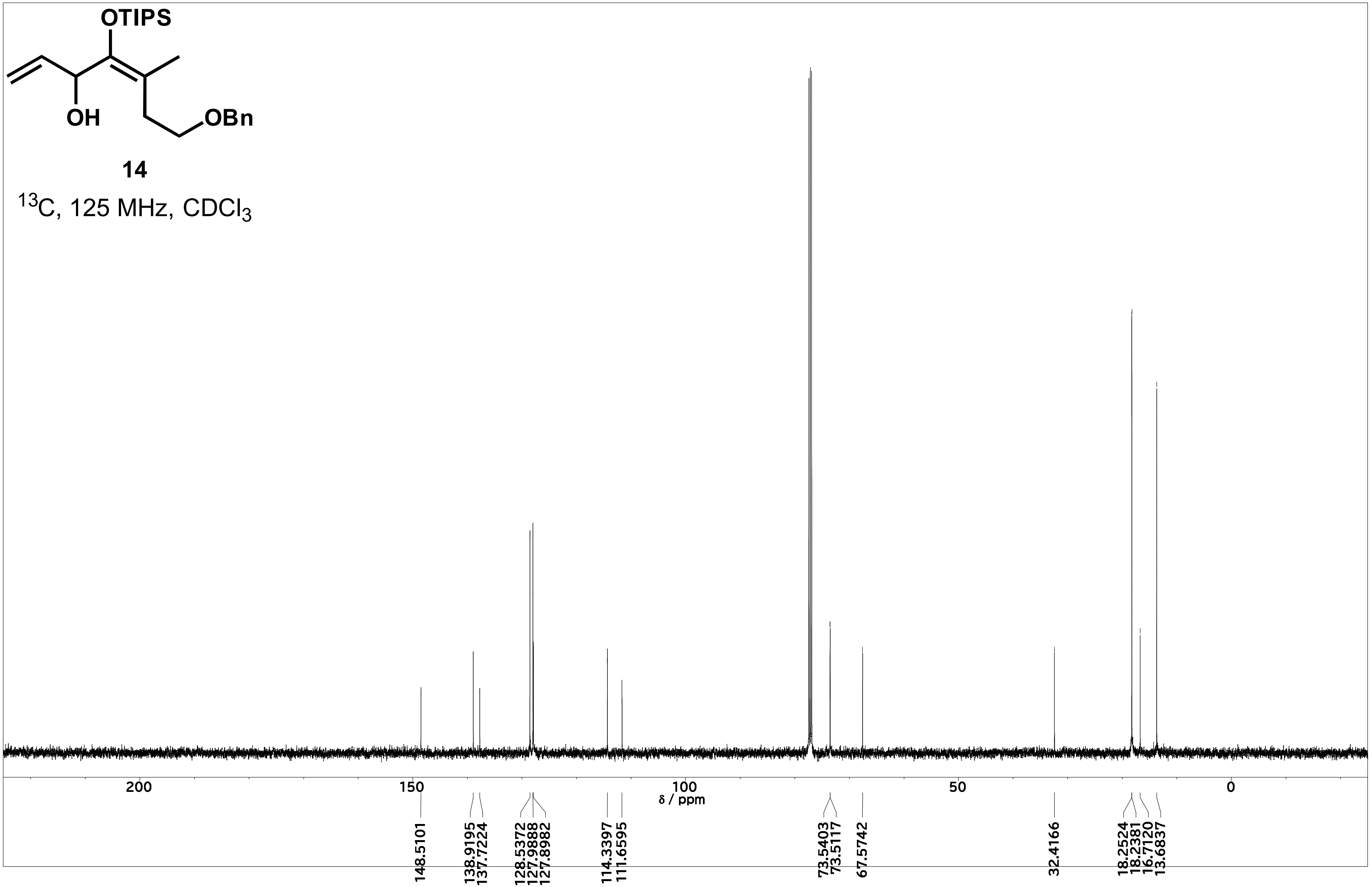
OTIPS
OH
OBn
14
13C, 125 MHz, CDCl3
200
150
100
δ / ppm
50
0
148.5101
138.9195
137.7224
128.5372
127.9888
127.8982
114.3397
111.6595
73.5403
73.5117
67.5742
32.4166
18.2524
18.2381
16.7120
13.6837

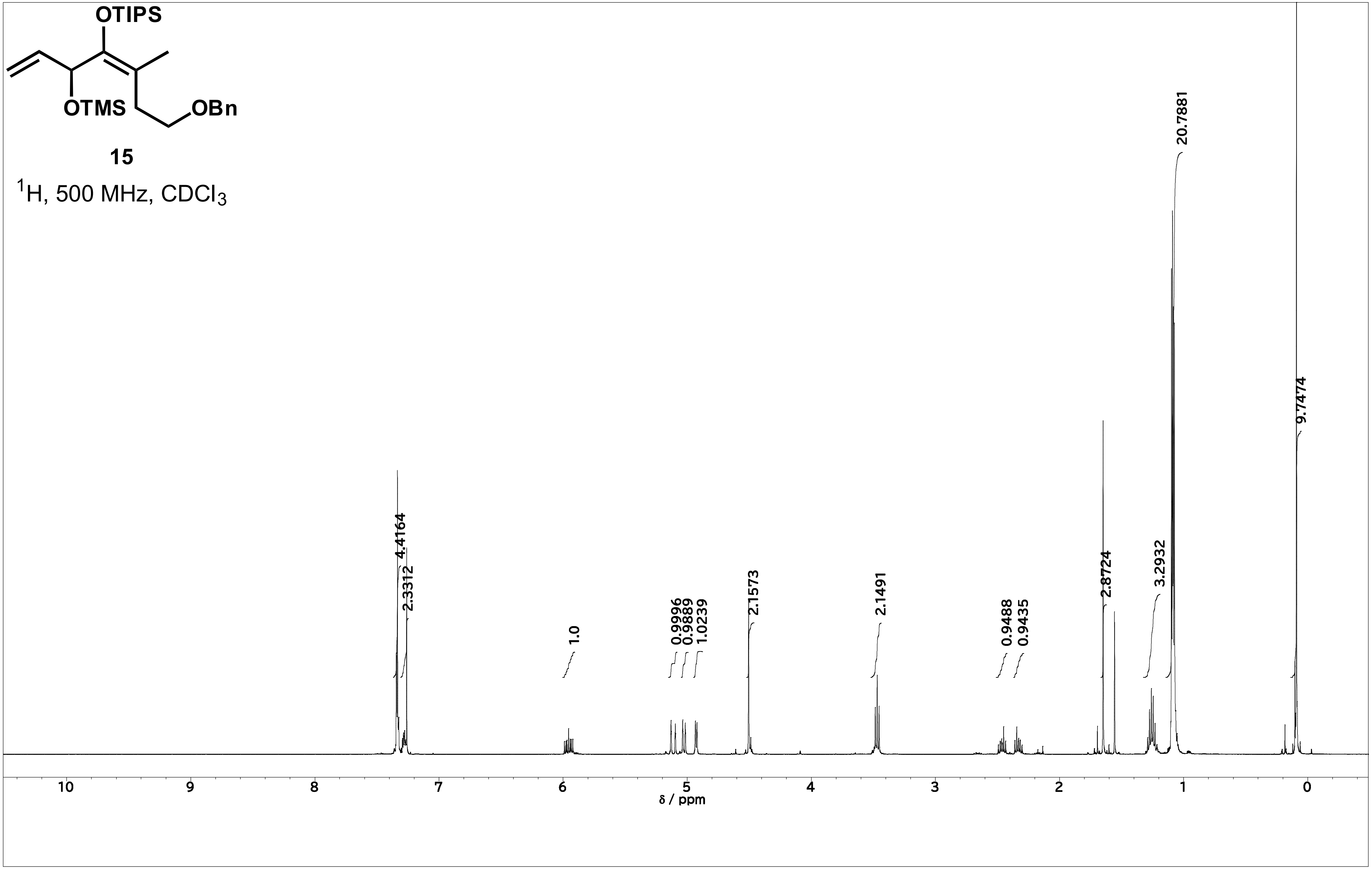
OTIPS
OTMS
OBn
15
1H, 500 MHz, CDCl3
4.4164
2.3312
1.0
0.9996
0.9889
1.0239
2.1573
2.1491
0.9488
0.9435
2.8724
3.2932
20.7881
9.7474
10
9
8
7
6
5
4
3
2
1
0
δ / ppm

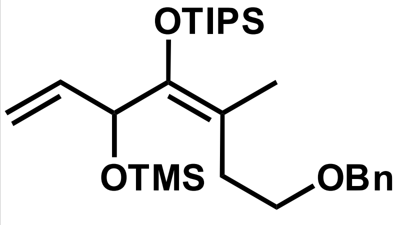

15

^{13}C, 125 MHz, $CDCl_3$

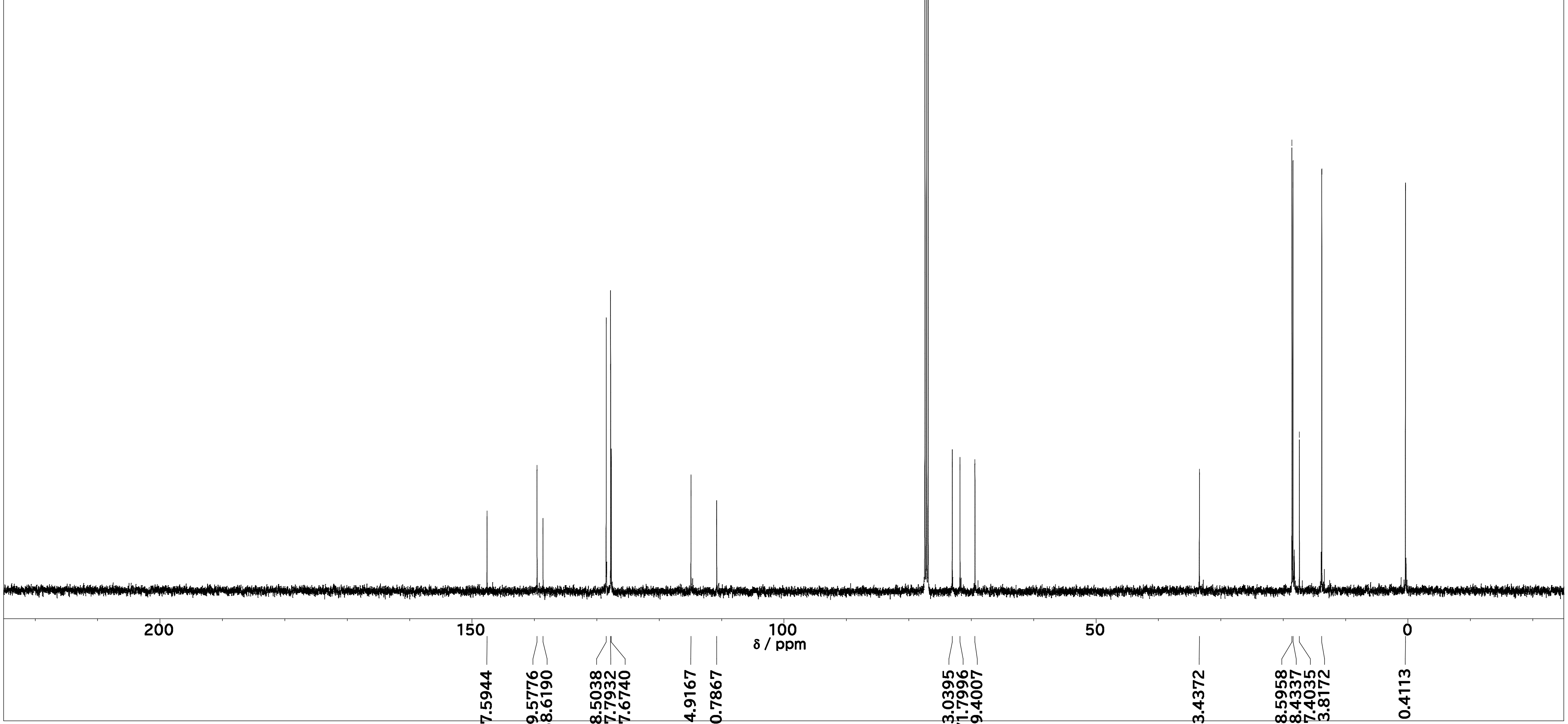

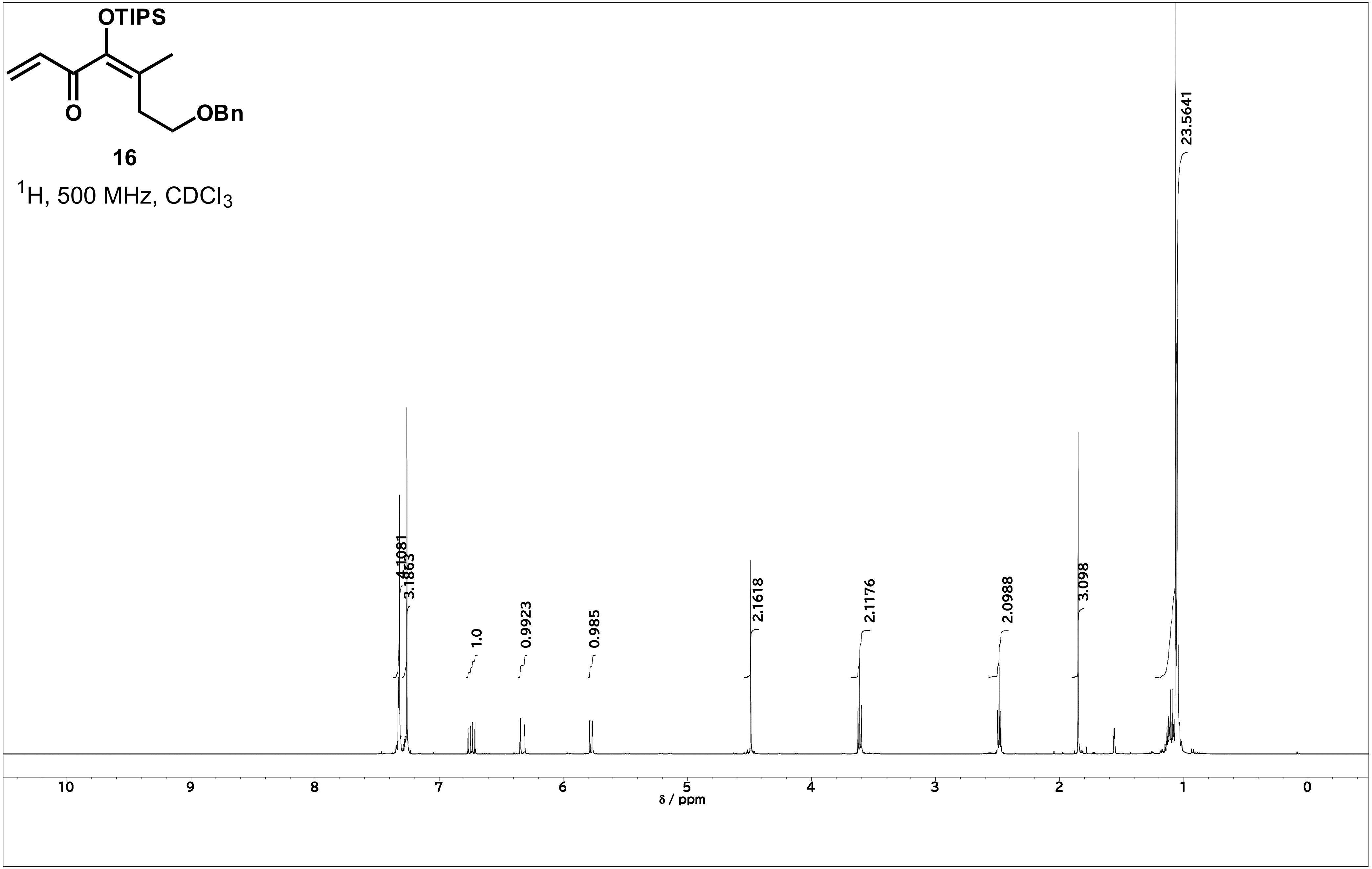
OTIPS
O
OBn
16
1H, 500 MHz, CDCl3
4.1081
3.1863
1.0
0.9923
0.985
2.1618
2.1176
2.0988
3.098
23.5641
10
9
8
7
6
5
4
3
2
1
0
δ / ppm

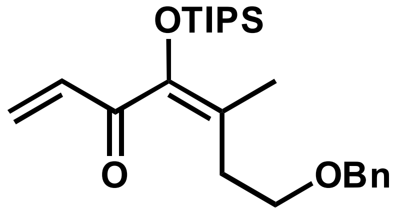

16

^{13}C, 125 MHz, $(CD_3)_2CO$

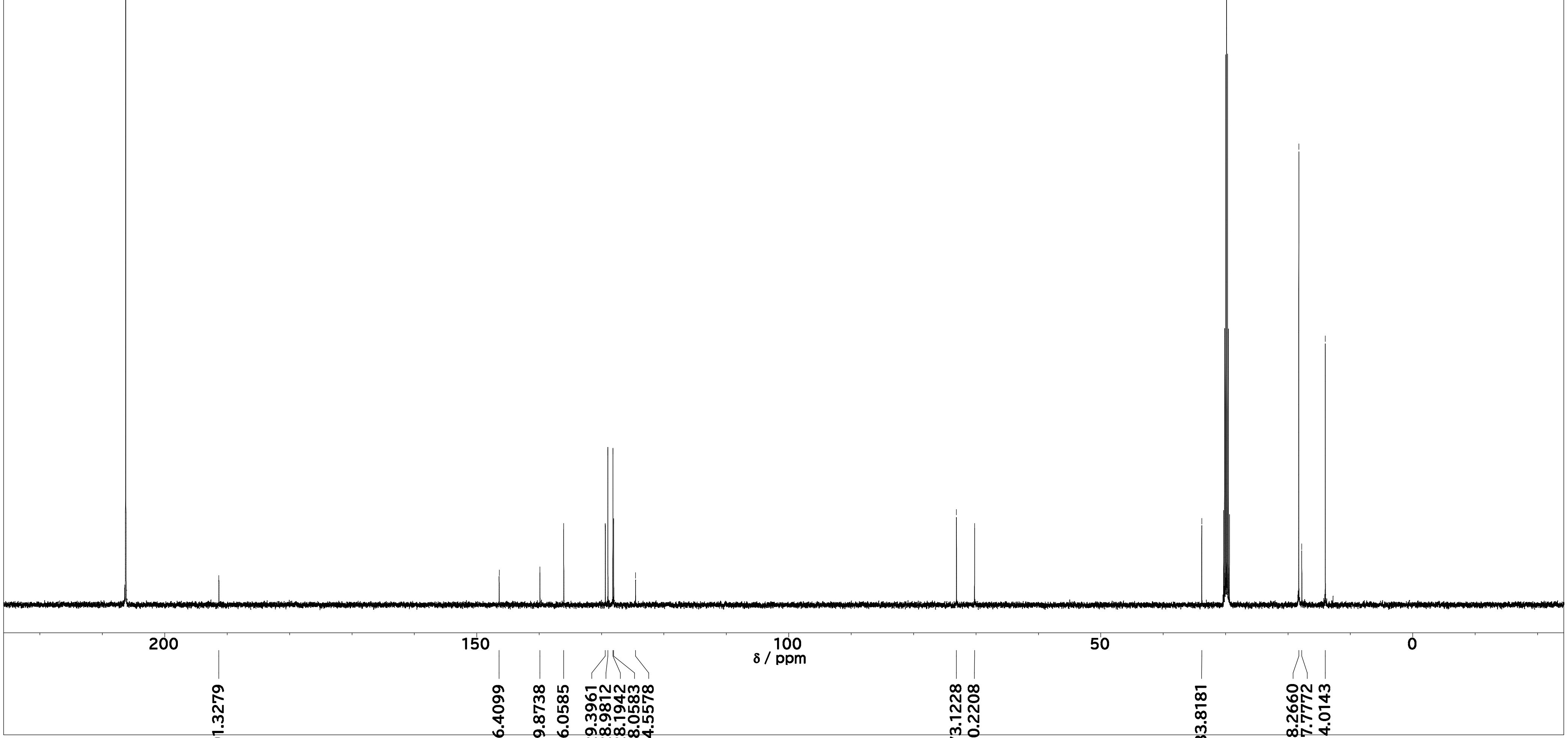

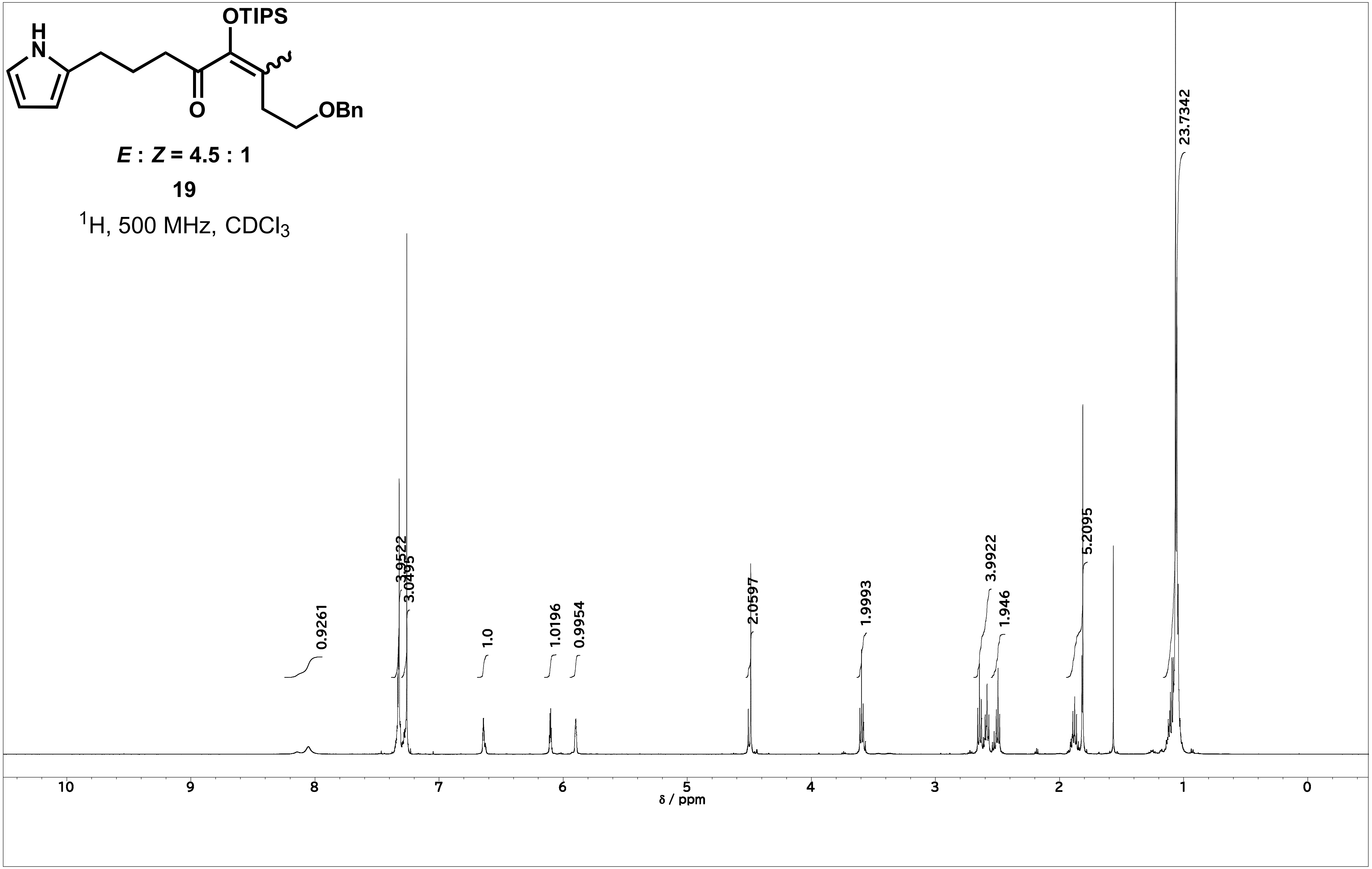
OTIPS
H
N
O
OBn
E : *Z* = 4.5 : 1
19
^{1}H, 500 MHz, $CDCl_3$
0.9261
3.9522
3.0495
1.0
1.0196
0.9954
2.0597
1.9993
3.9922
1.946
5.2095
23.7342
10
9
8
7
6
5
4
3
2
1
0
δ / ppm

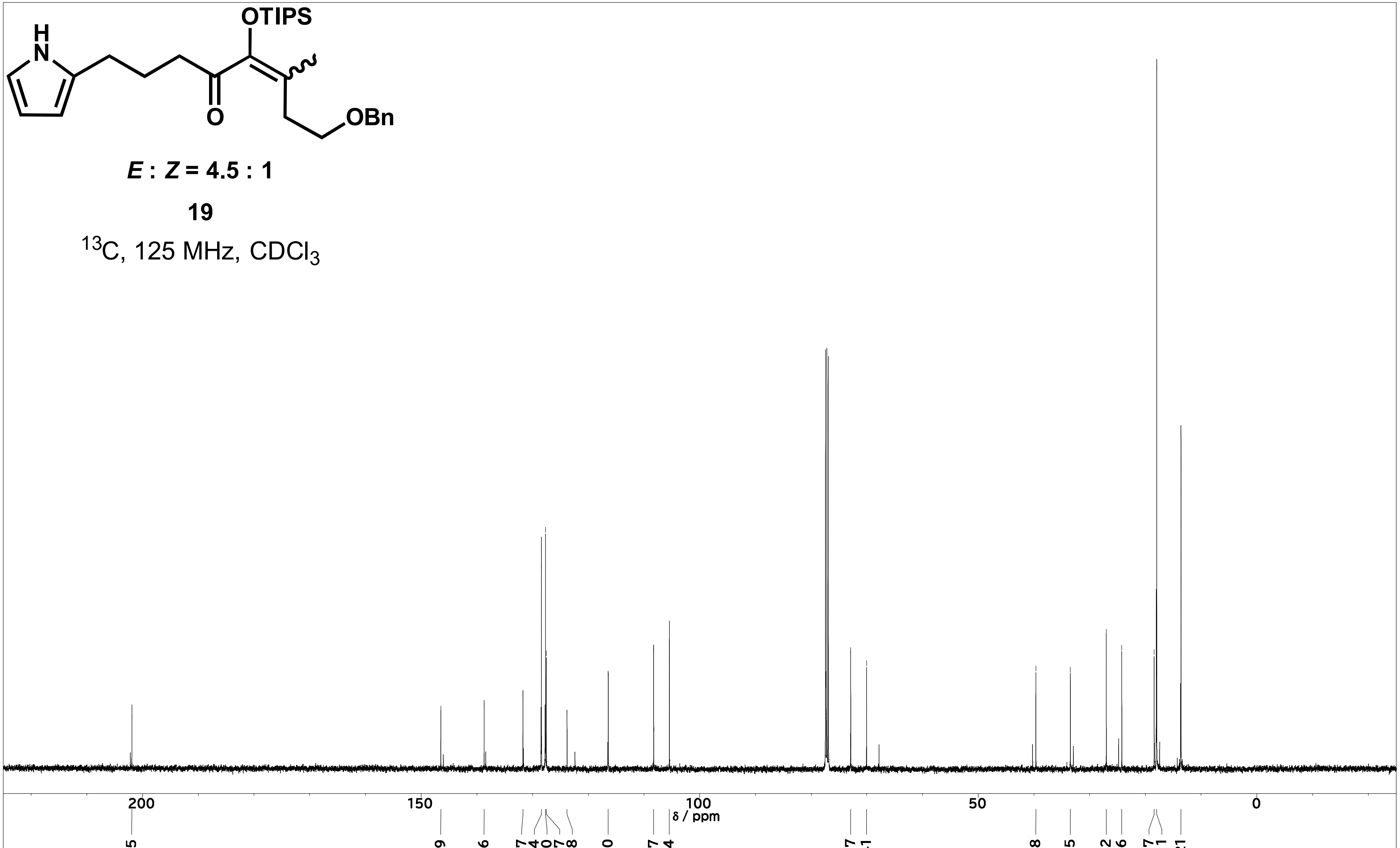
OTIPS
H
N
O
OBn
E : Z = 4.5 : 1
19
13C, 125 MHz, CDCl3
200
150
100
δ / ppm
50
0
201.9285
146.4689
138.6906
131.7277
128.4084
127.6740
127.5357
123.8158
116.4190
108.2687
105.4454
72.8917
70.0541
39.6608
33.4705
27.0132
24.2376
18.4337
17.9711
13.6121

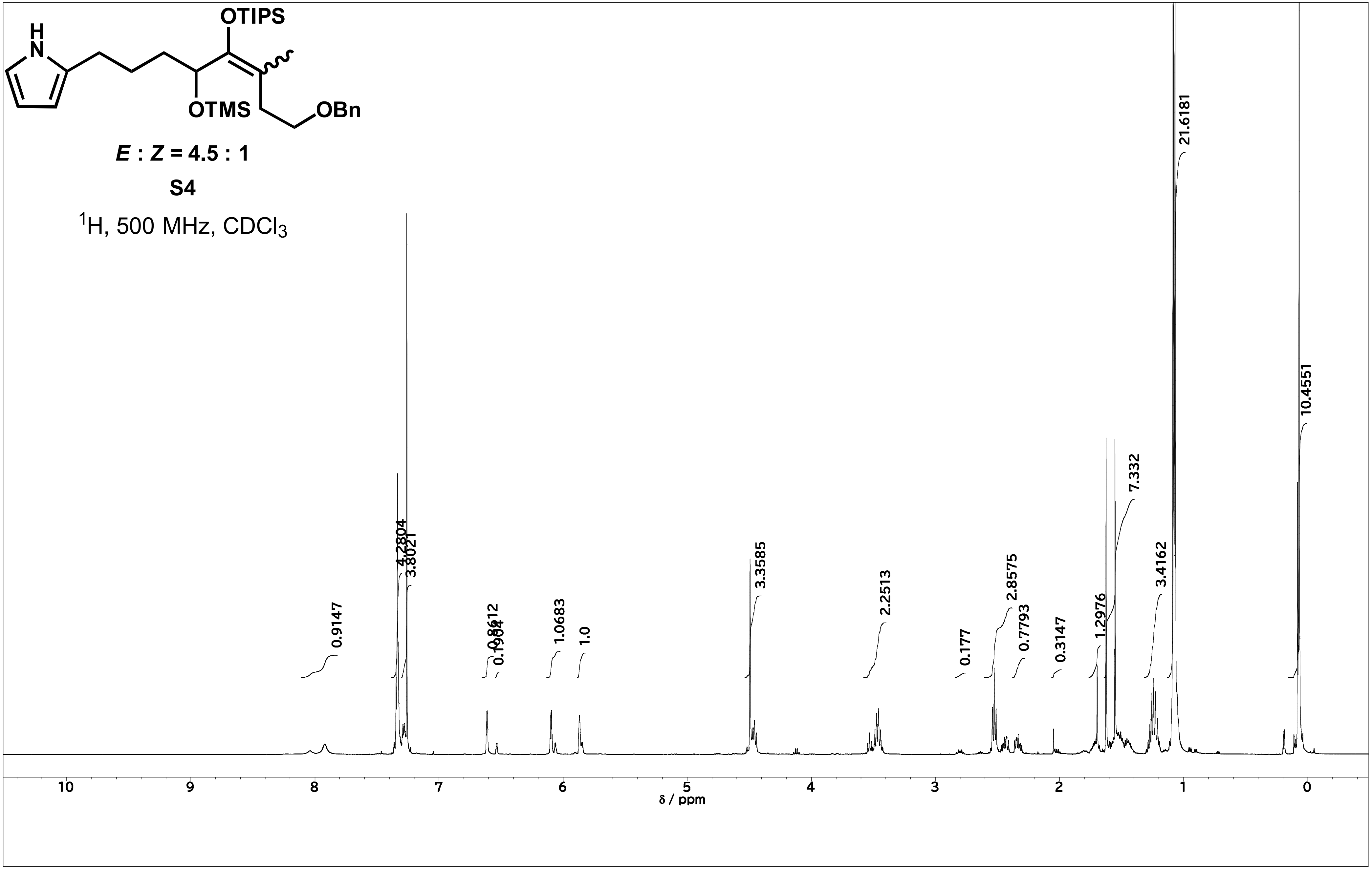

OTIPS
H
N
OTMS
OBn
E : Z = 4.5 : 1
S4
1H, 500 MHz, CDCl3
0.9147
4.2804
3.8021
0.8612
0.1904
1.0683
1.0
3.3585
2.2513
0.177
2.8575
0.7793
0.3147
1.2976
7.332
3.4162
21.6181
10.4551
10
9
8
7
6
5
4
3
2
1
0
δ / ppm

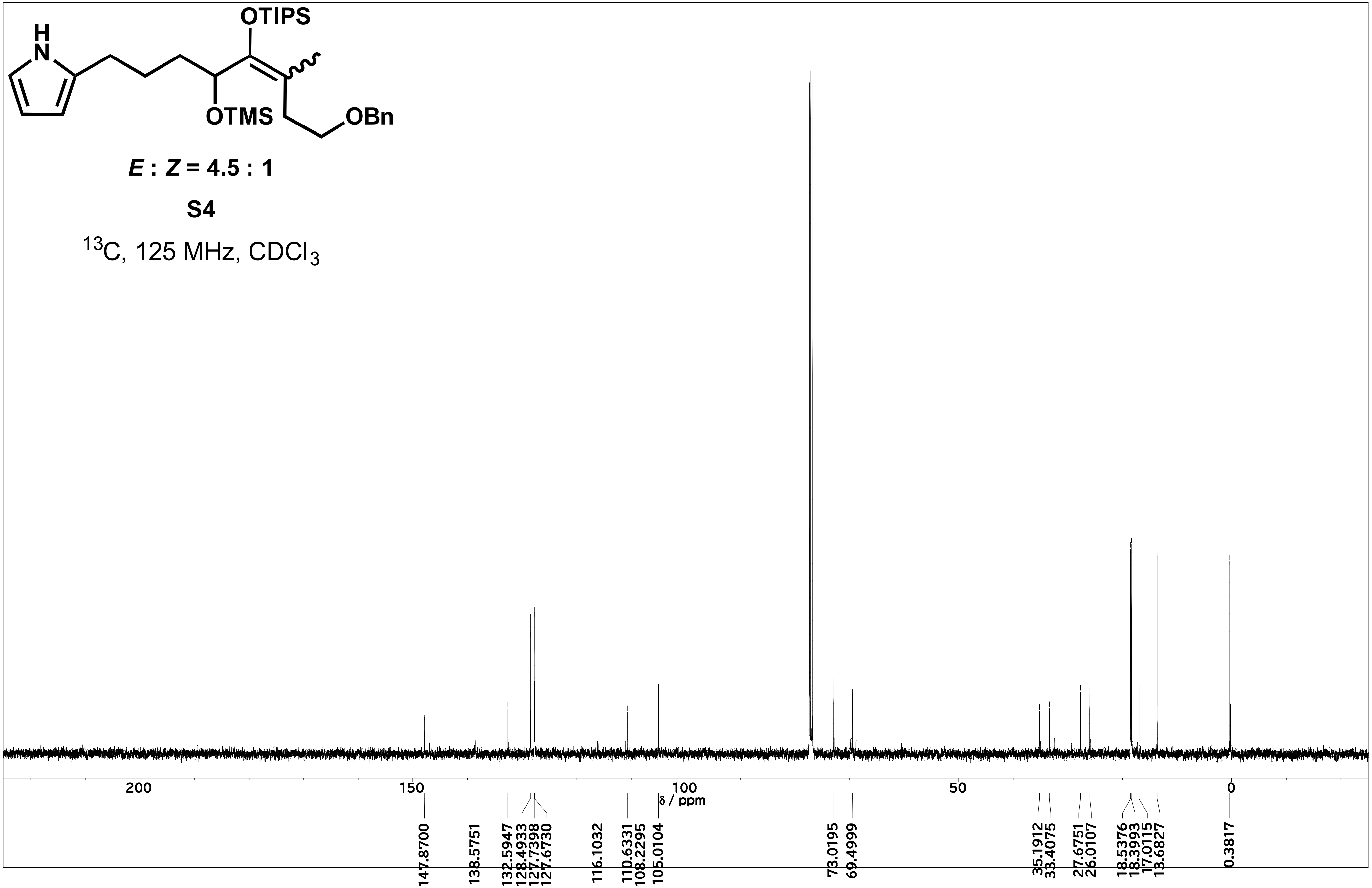

OTIPS
H
N
OTMS
OBn
E : *Z* = 4.5 : 1
S4
^{13}C, 125 MHz, CDCl$_3$
200
150
100
δ / ppm
50
0
147.8700
138.5751
132.5947
128.4933
127.7398
127.6730
116.1032
110.6331
108.2295
105.0104
73.0195
69.4999
35.1912
33.4075
27.6751
26.0107
18.5376
18.3993
17.0115
13.6827
0.3817

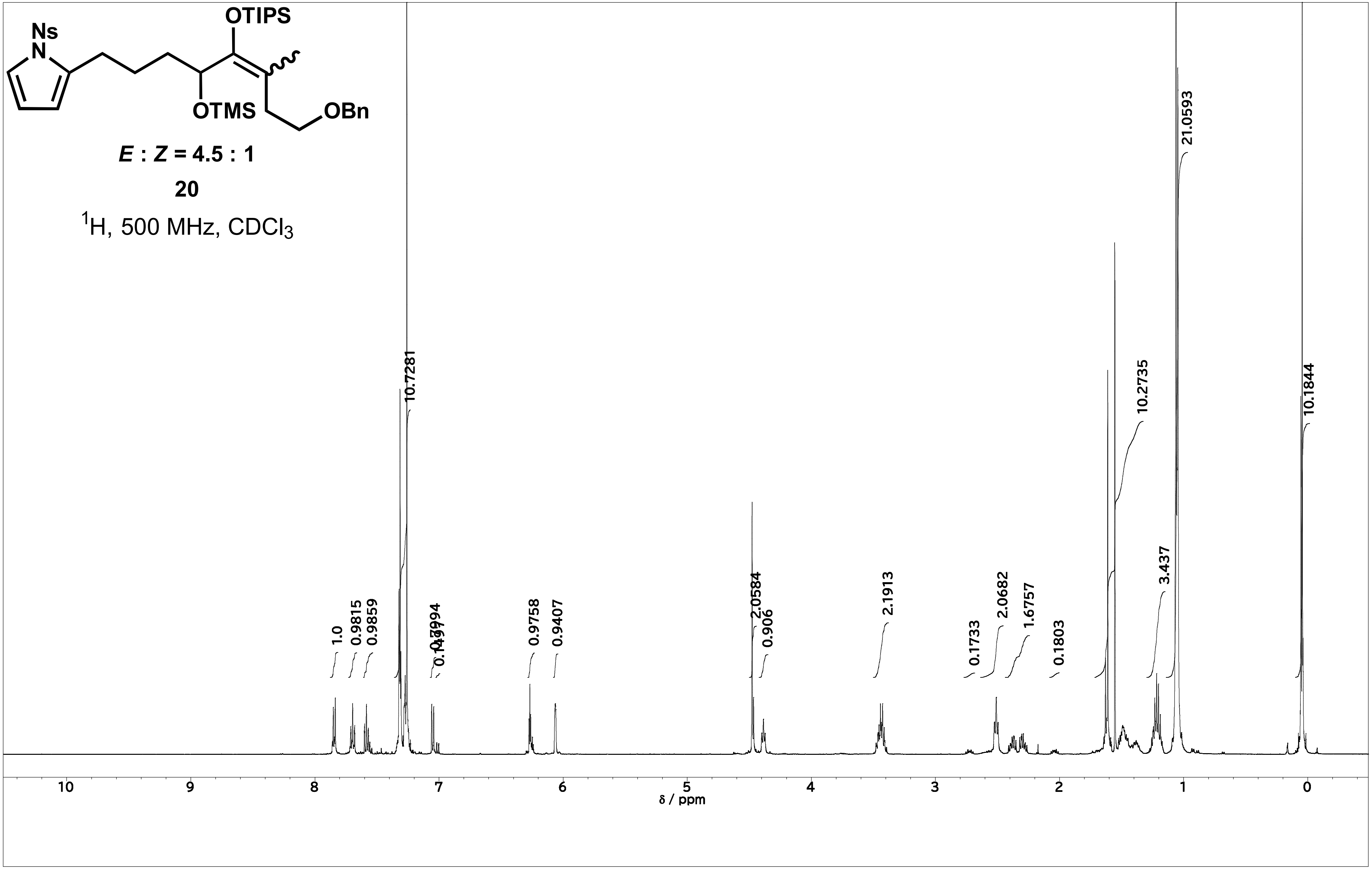
Ns
N
OTIPS
OTMS
OBn
E : Z = 4.5 : 1
20
1H, 500 MHz, CDCl3
10.7281
10.2735
21.0593
10.1844
1.0
0.9815
0.9859
0.7994
0.1497
0.9758
0.9407
2.0584
0.906
2.1913
0.1733
2.0682
1.6757
0.1803
3.437
10
9
8
7
6
5
4
3
2
1
0
δ / ppm

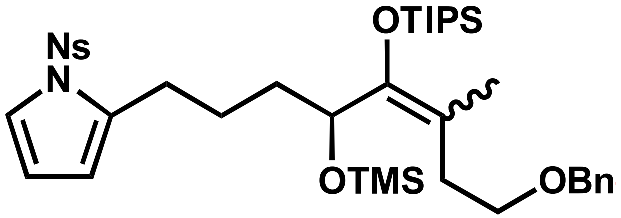

***E* : *Z* = 4.5 : 1**

20

^{13}C, 125 MHz, $CDCl_3$

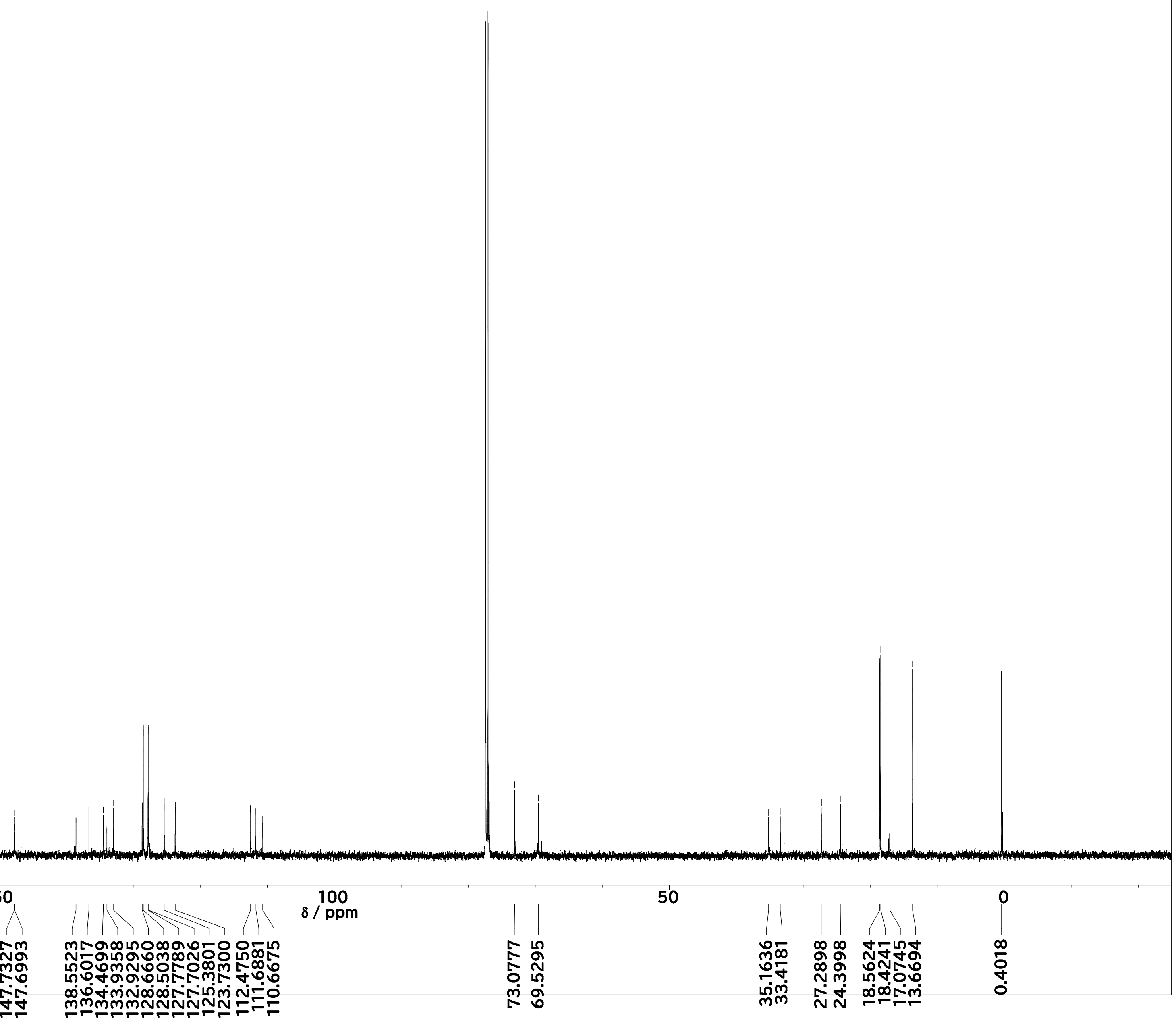

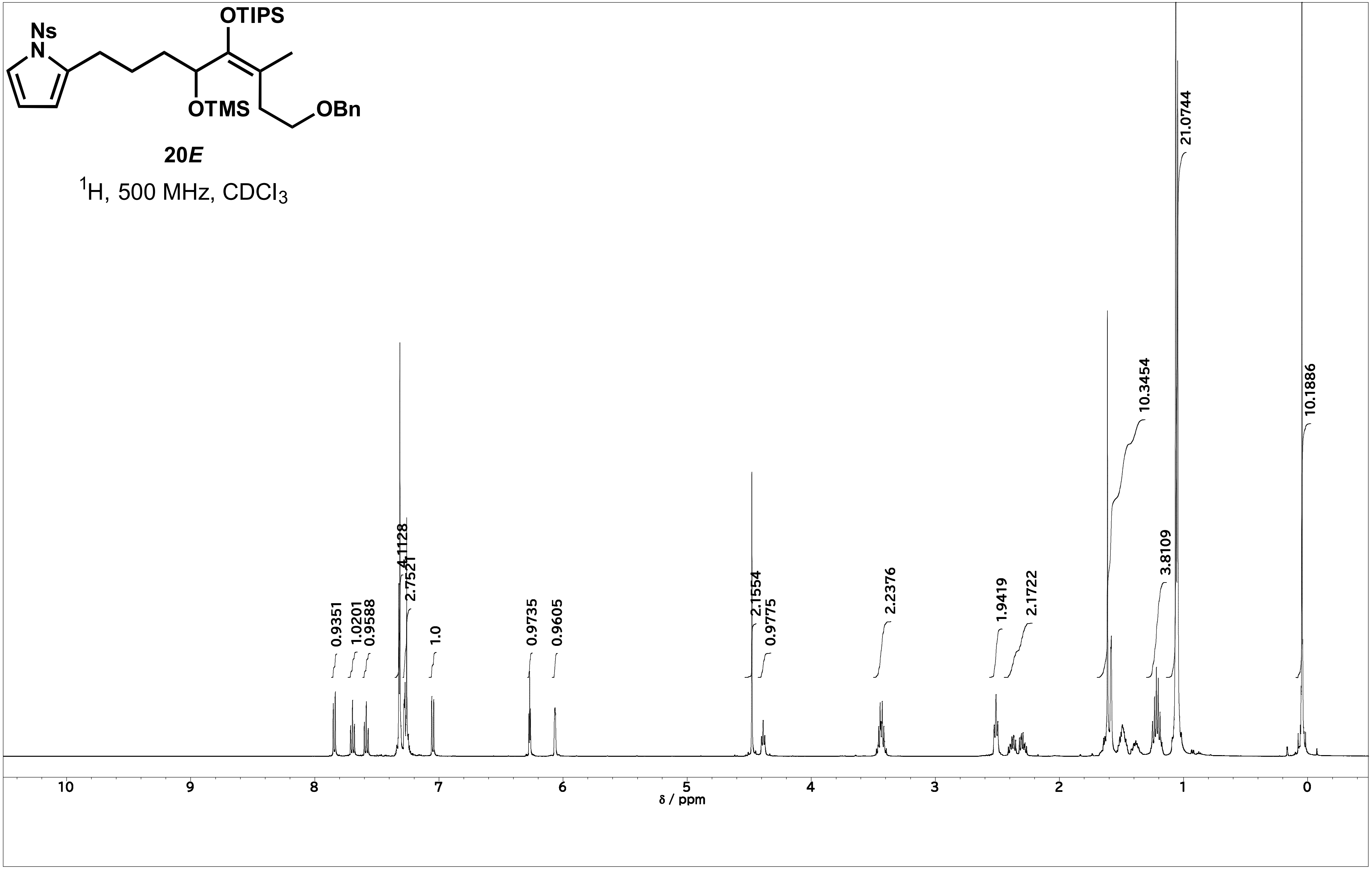
Ns
N
OTIPS
OTMS
OBn
20*E*
^{1}H, 500 MHz, $CDCl_3$
0.9351
1.0201
0.9588
4.1128
2.7521
1.0
0.9735
0.9605
2.1554
0.9775
2.2376
1.9419
2.1722
10.3454
3.8109
21.0744
10.1886
10
9
8
7
6
5
4
3
2
1
0
δ / ppm

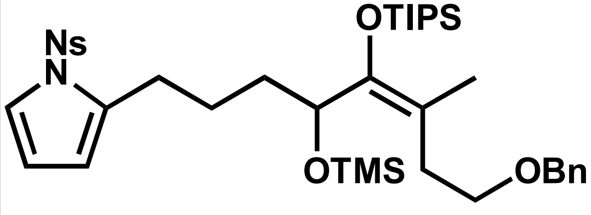

20*E*

^{13}C, 125 MHz, $CDCl_3$

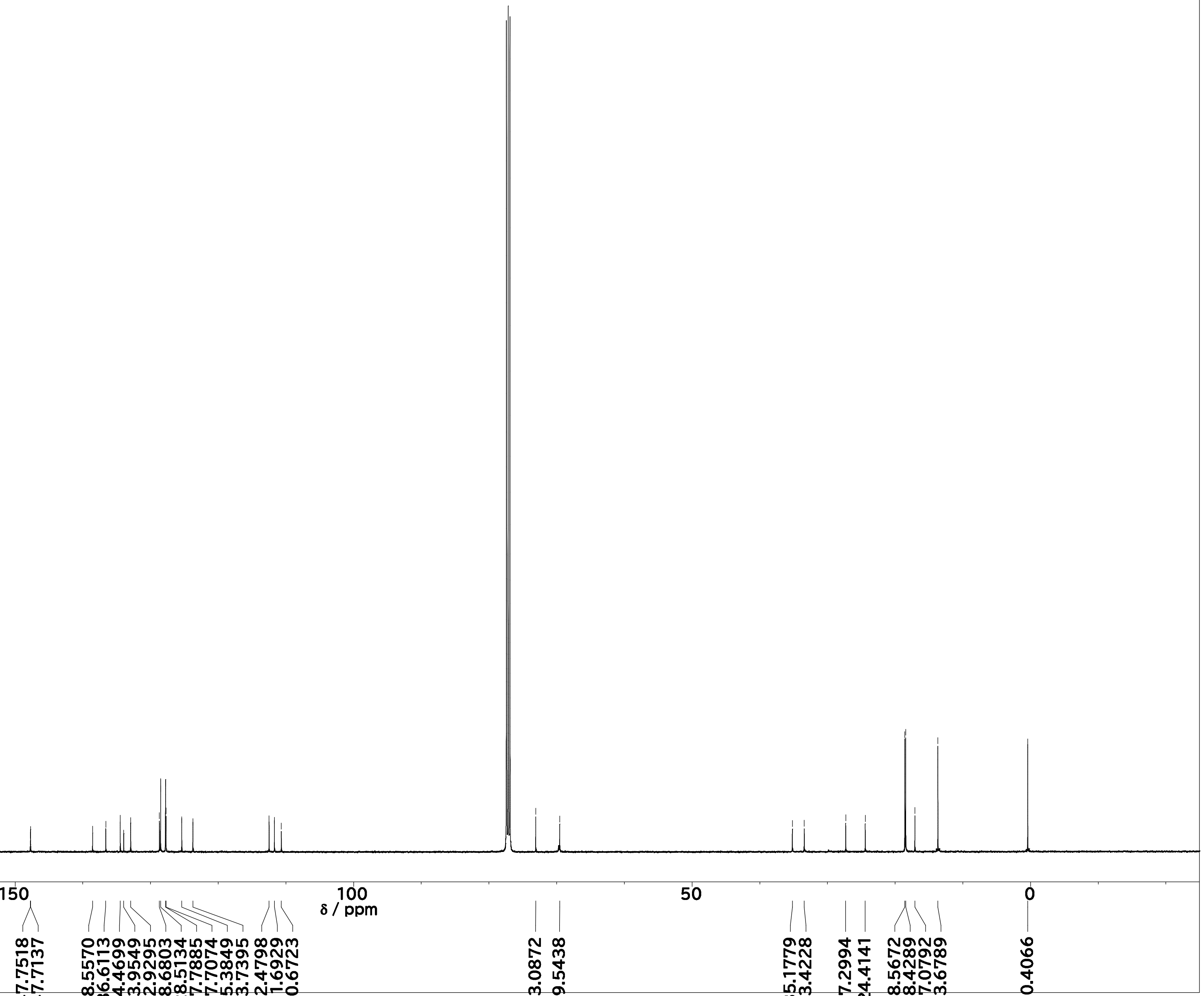

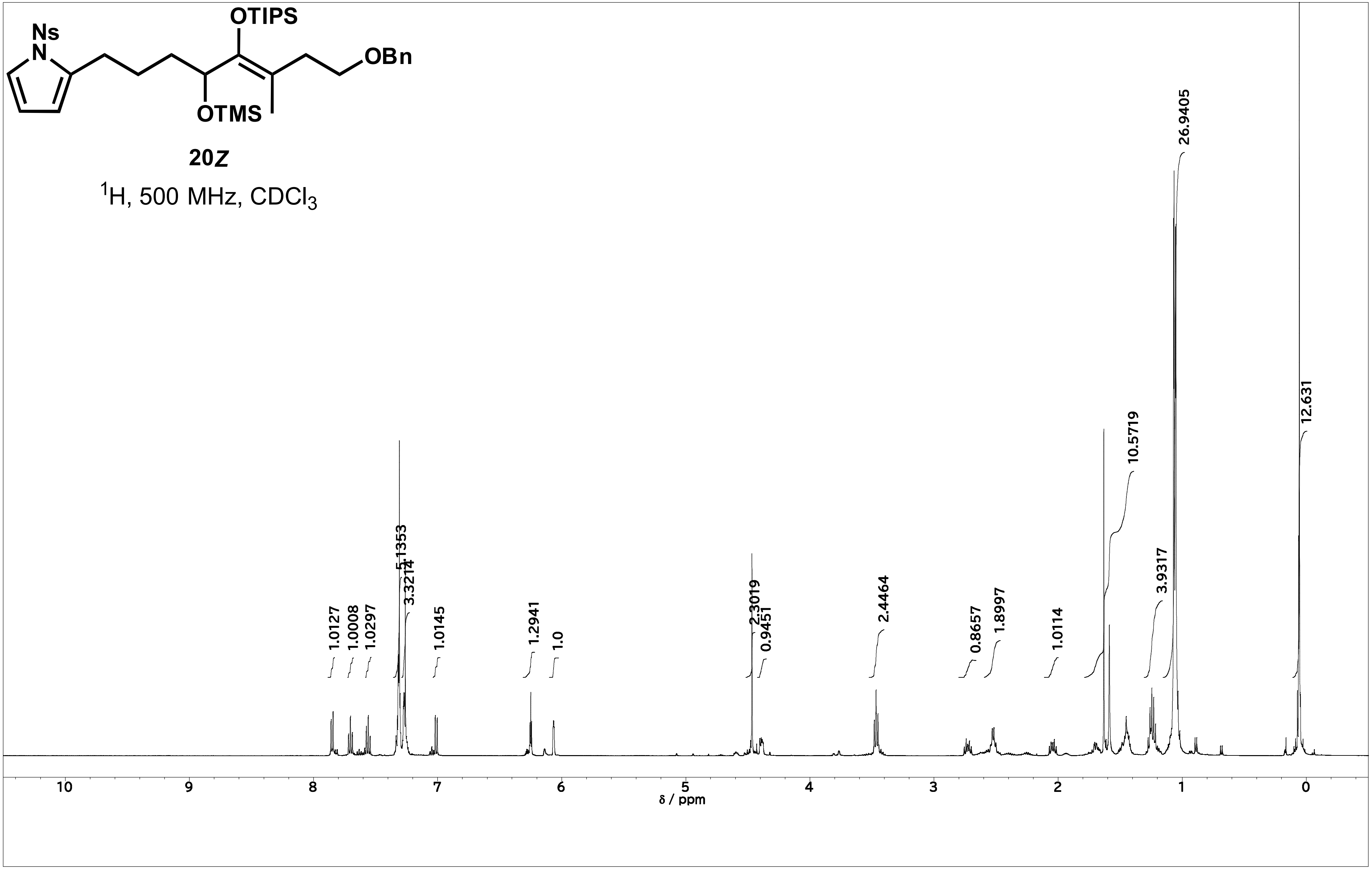
Ns
N
OTIPS
OBn
OTMS
20Z
^{1}H, 500 MHz, $CDCl_3$
1.0127
1.0008
1.0297
5.1353
3.3214
1.0145
1.2941
1.0
2.3019
0.9451
2.4464
0.8657
1.8997
1.0114
10.5719
3.9317
26.9405
12.631
10
9
8
7
6
5
4
3
2
1
0
δ / ppm

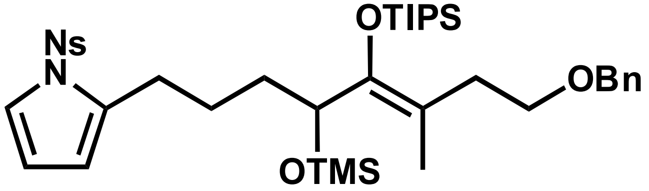

20*Z*

^{13}C, 125 MHz, $CDCl_3$

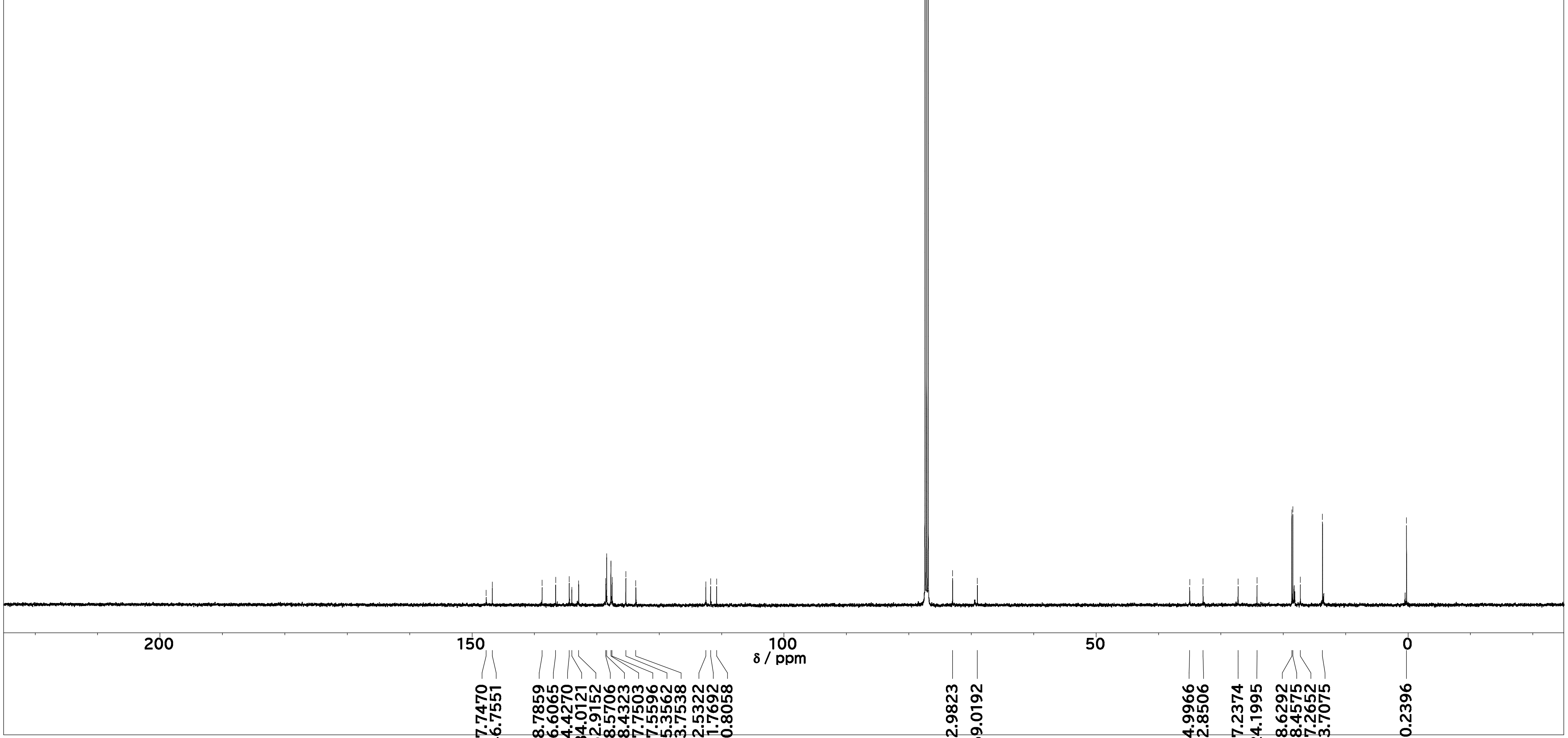

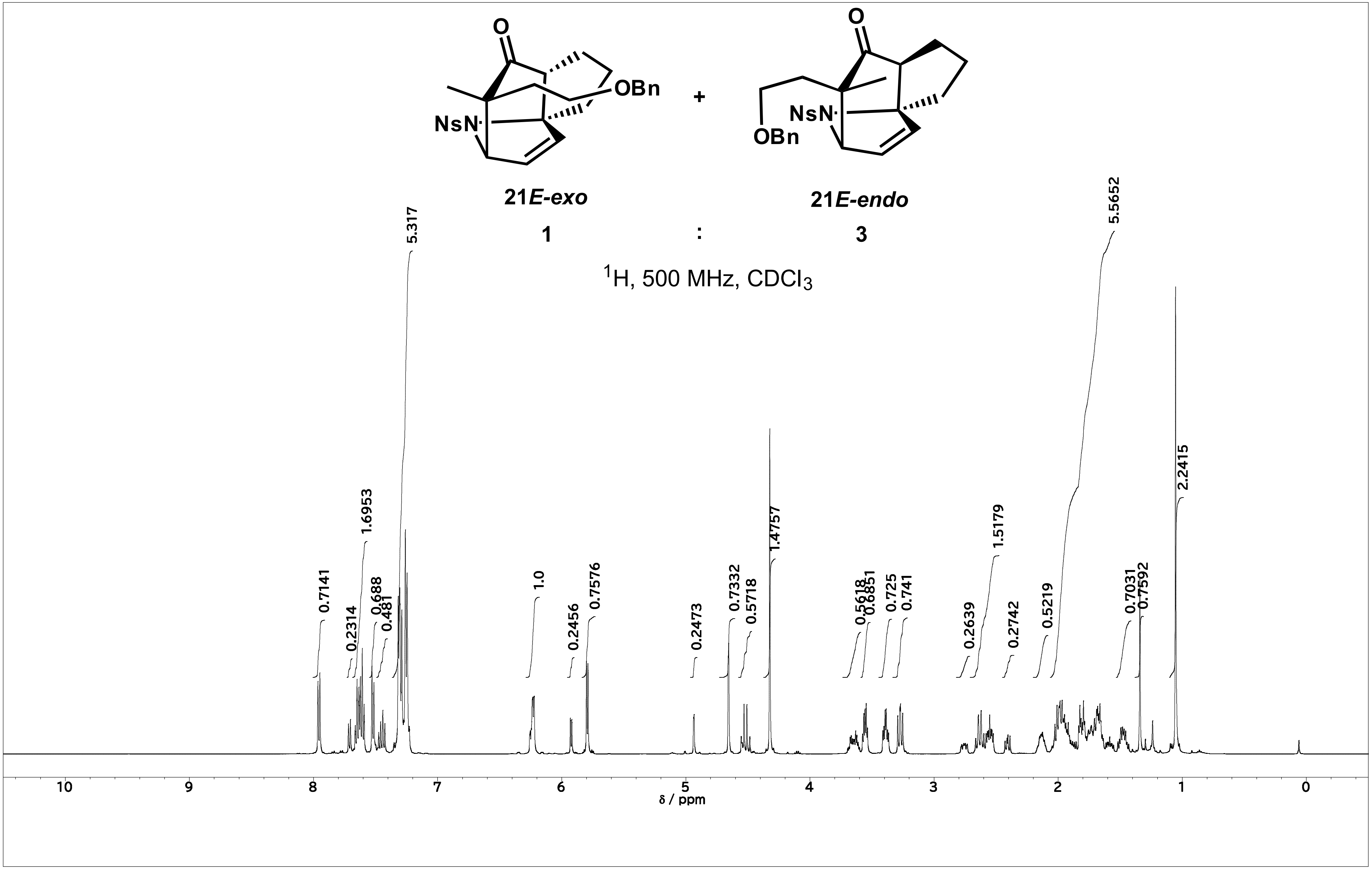

NsN
OBn
NsN
OBn
21E-exo
21E-endo
1
:
3
1H, 500 MHz, CDCl3
δ / ppm

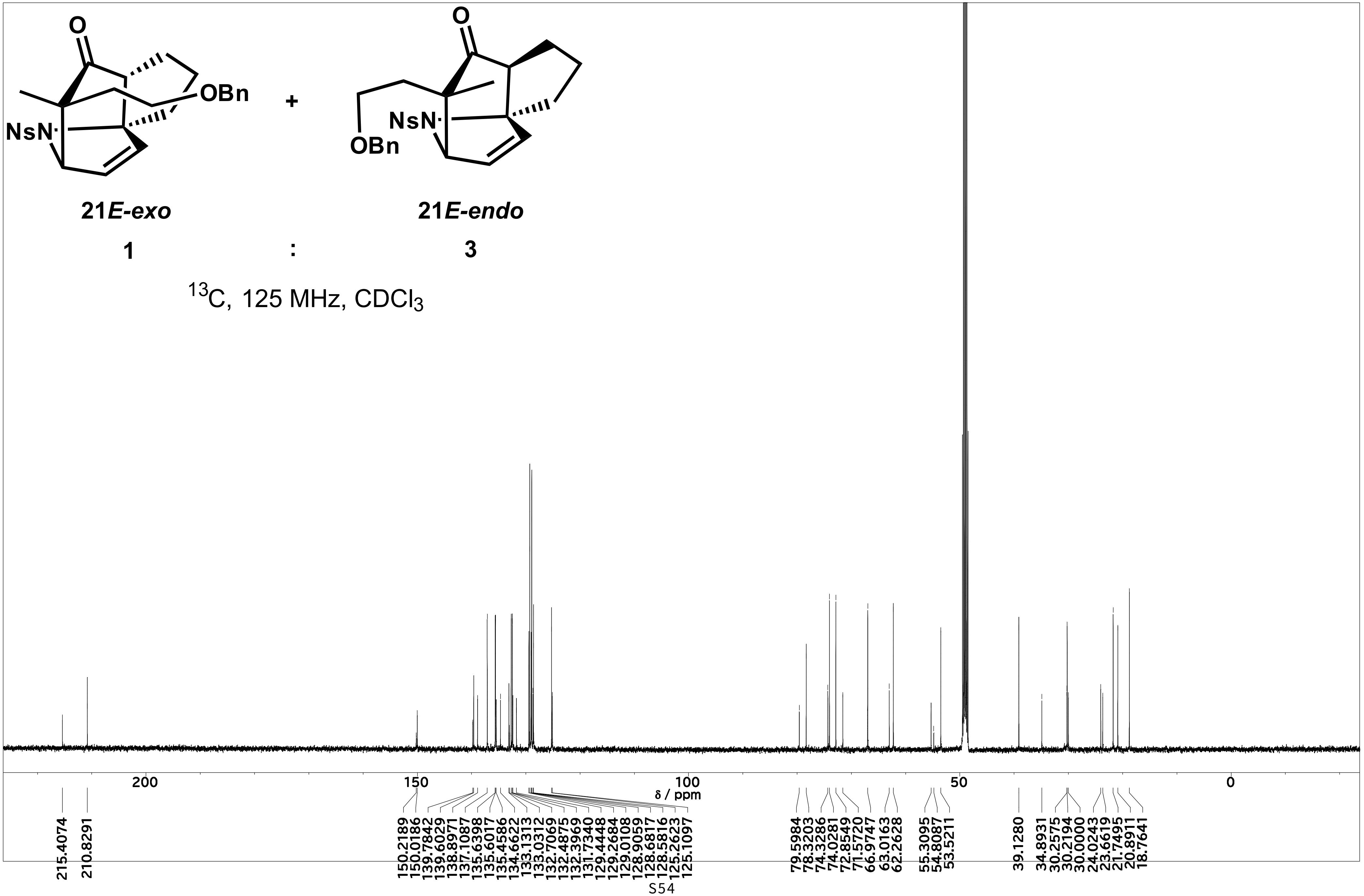

NsN
OBn
O
21E-exo
1
:
21E-endo
3
13C, 125 MHz, CDCl3
δ / ppm
200
150
100
50
0
215.4074
210.8291
150.2189
150.0186
139.7842
139.6029
138.8971
137.1087
135.6398
135.6017
135.4586
134.6622
133.1313
133.0312
132.7069
132.4875
132.3969
131.7340
129.4448
129.2684
129.0108
128.9059
128.6817
128.5816
125.2623
125.1097
79.5984
78.3203
74.3286
74.0281
72.8549
71.5720
66.9747
63.0163
62.2628
55.3095
54.8087
53.5211
39.1280
34.8931
30.2575
30.2194
30.0000
24.0243
23.6619
21.7495
20.8911
18.7641

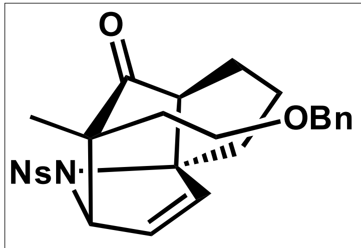

21*Z-endo*

^{1}H, 500 MHz, $CDCl_3$

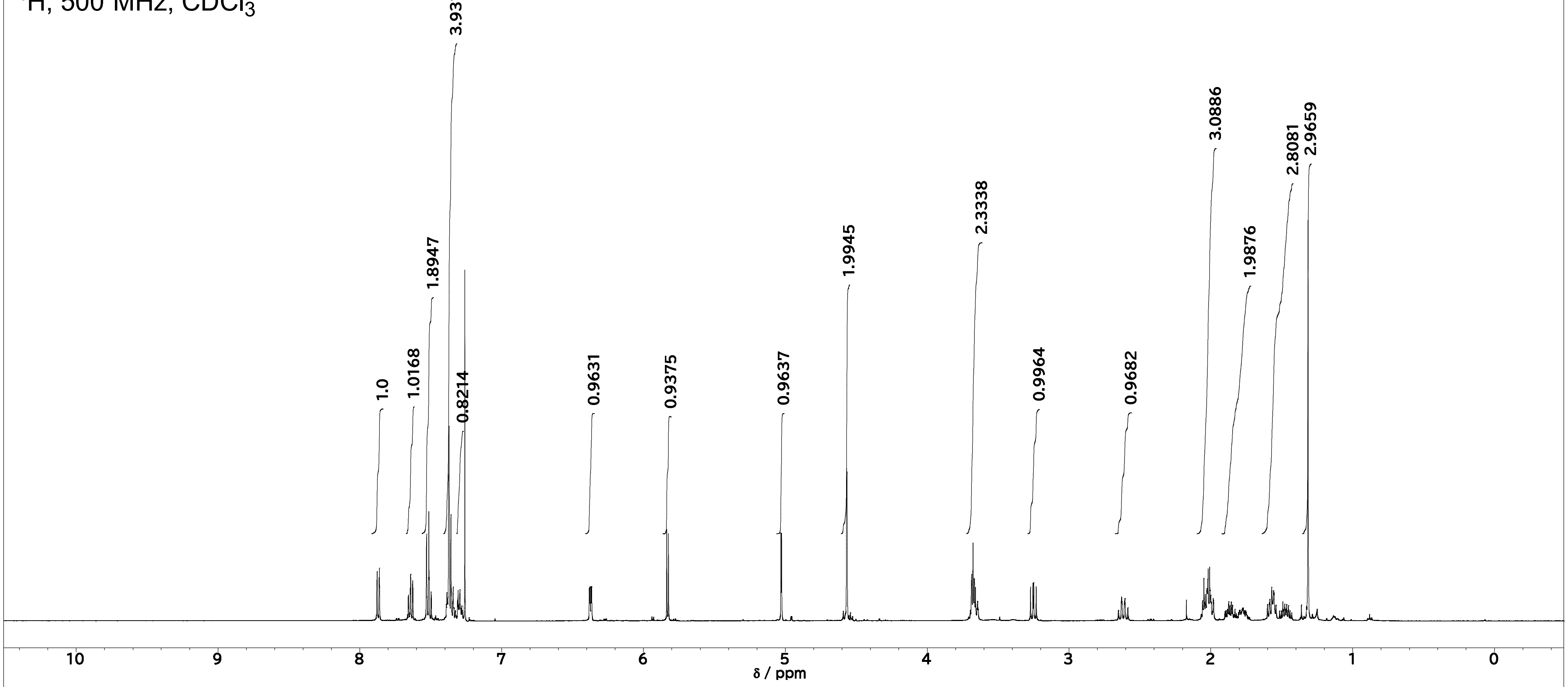

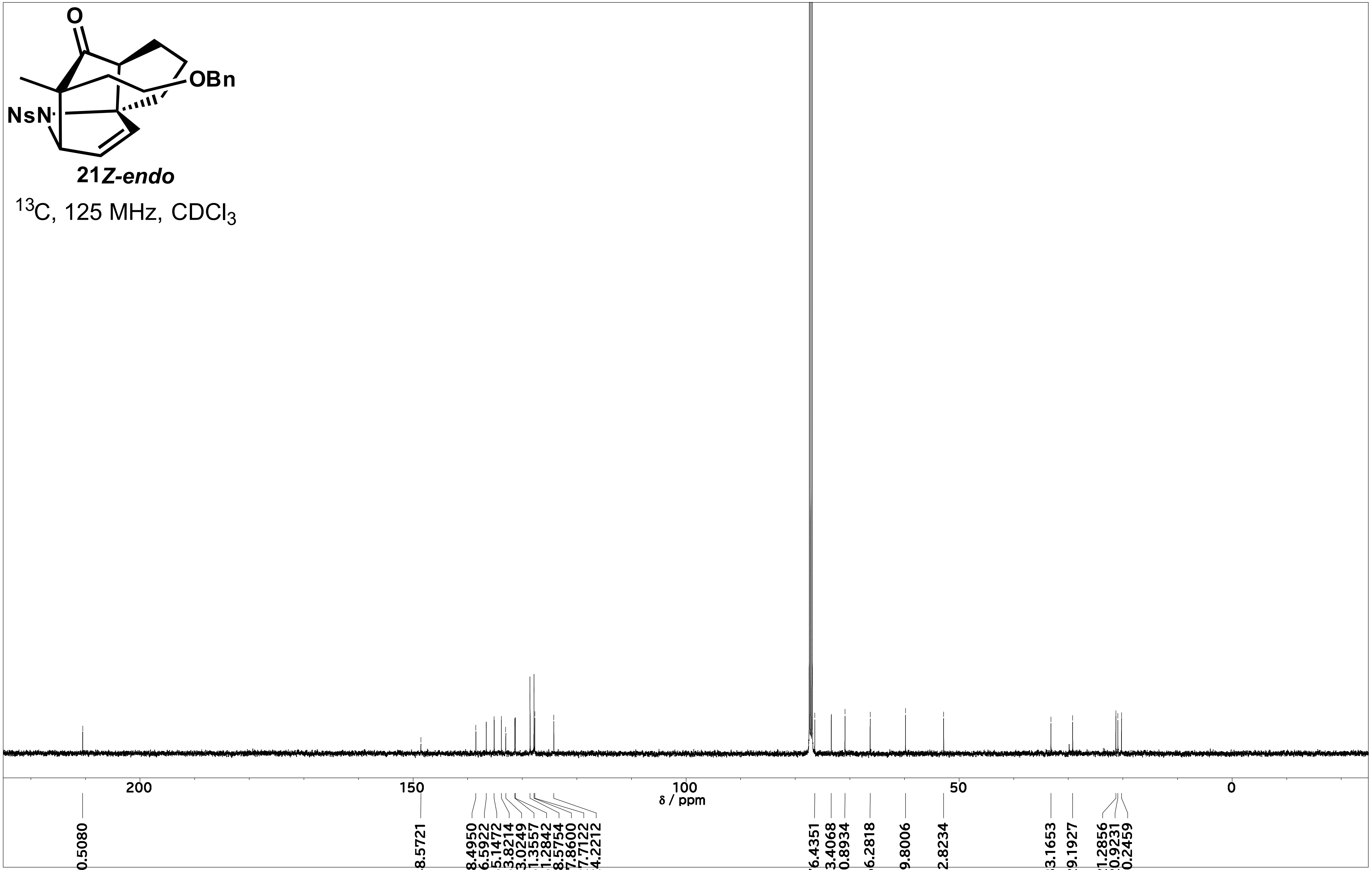
O
OBn
NsN
21Z-endo
13C, 125 MHz, CDCl3
200
150
100
50
0
δ / ppm
210.5080
148.5721
138.4950
136.5922
135.1472
133.8214
133.0249
131.3557
131.2842
128.5754
127.8600
127.7122
124.2212
76.4351
73.4068
70.8934
66.2818
59.8006
52.8234
33.1653
29.1927
21.2856
20.9231
20.2459

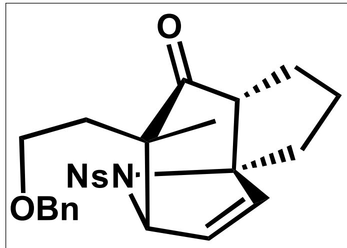

21*Z-exo*

^{1}H, 500 MHz, $CDCl_3$

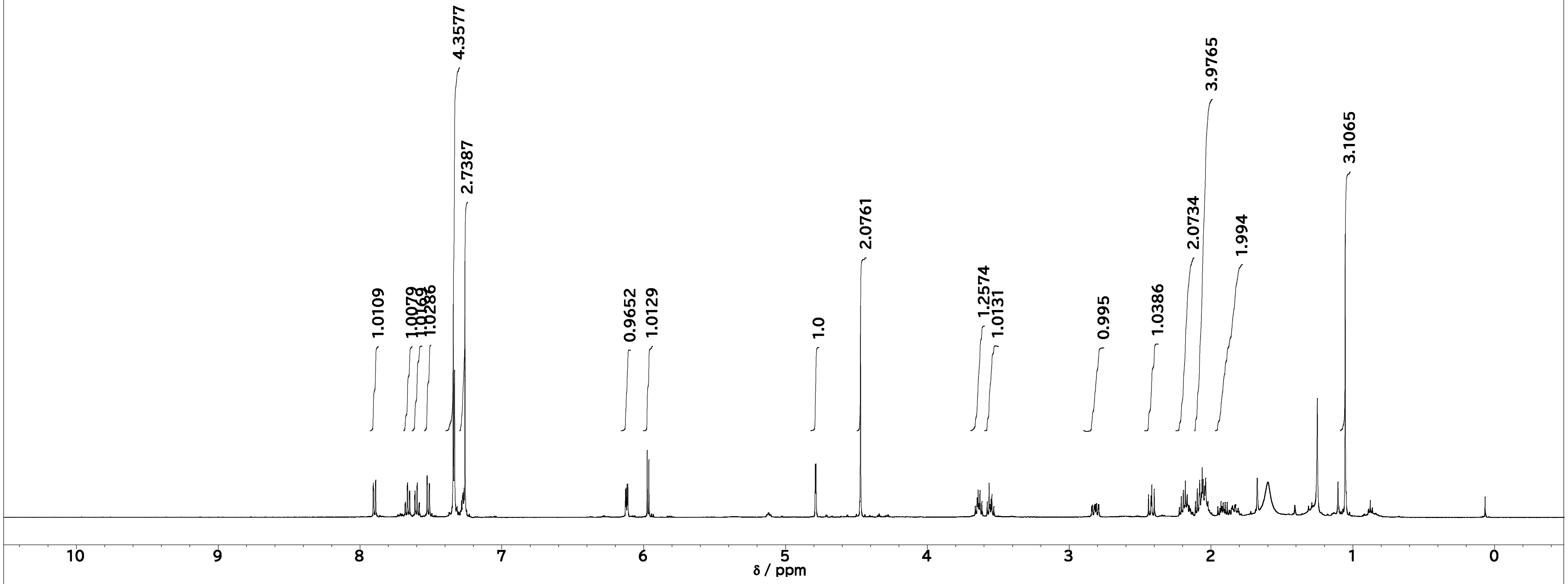

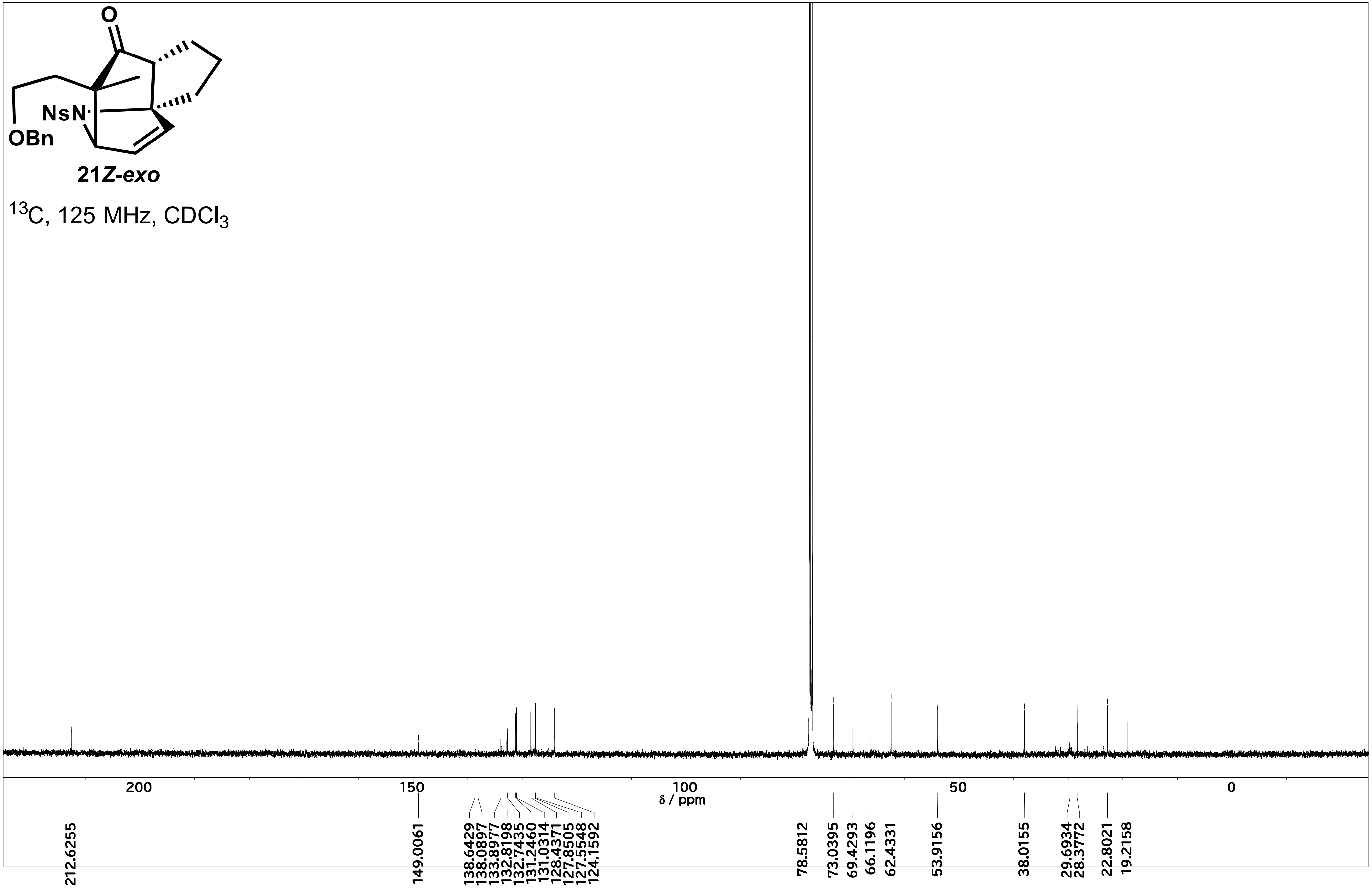
O
NsN
OBn
21Z-exo
13C, 125 MHz, CDCl3
200
150
100
50
0
δ / ppm
212.6255
149.0061
138.6429
138.0897
133.8977
132.8198
132.7435
131.2460
131.0314
128.4371
127.8505
127.5548
124.1592
78.5812
73.0395
69.4293
66.1196
62.4331
53.9156
38.0155
29.6934
28.3772
22.8021
19.2158

22*E*-*endo*

^{1}H, 500 MHz, CD_3OD

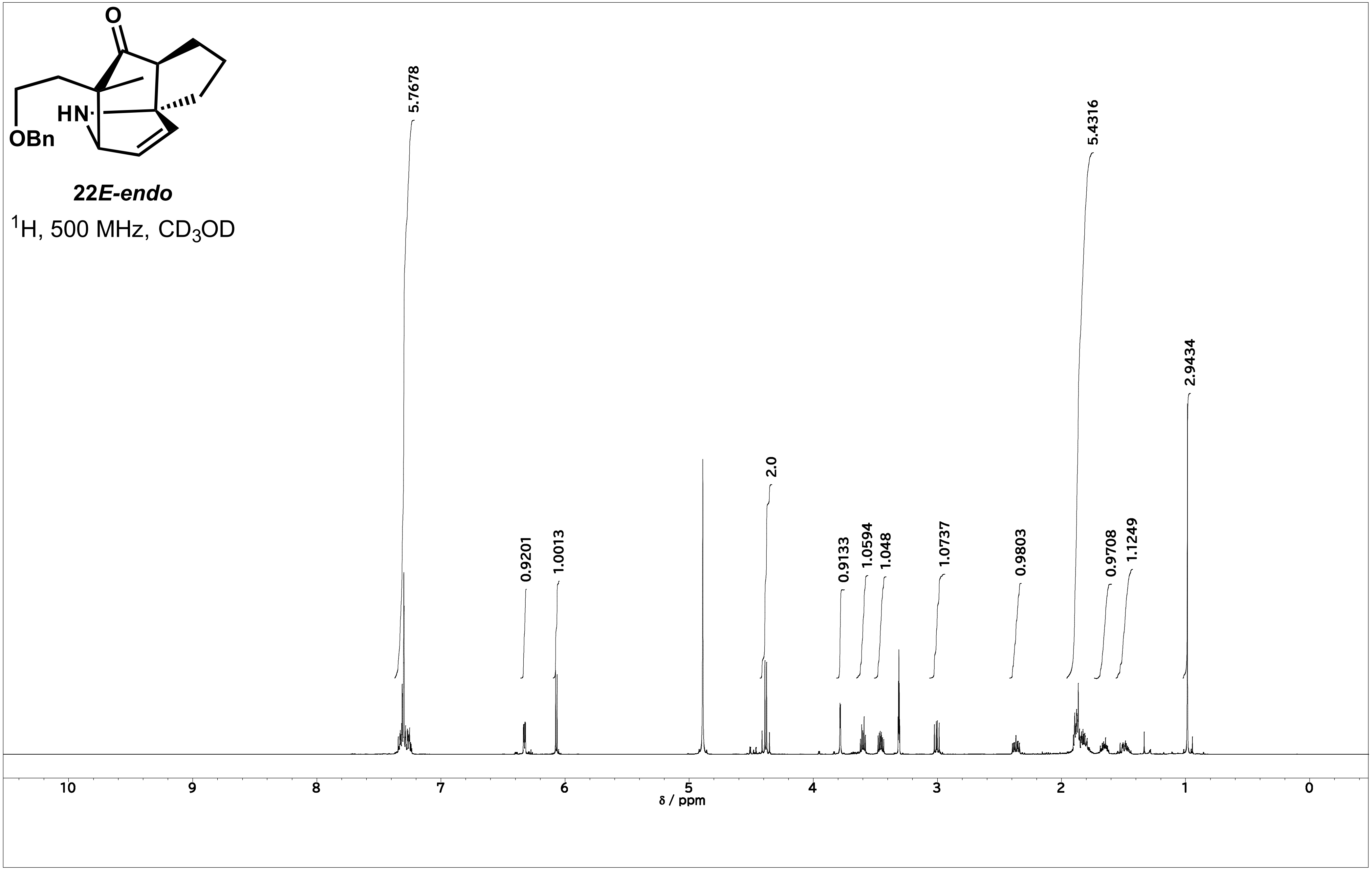

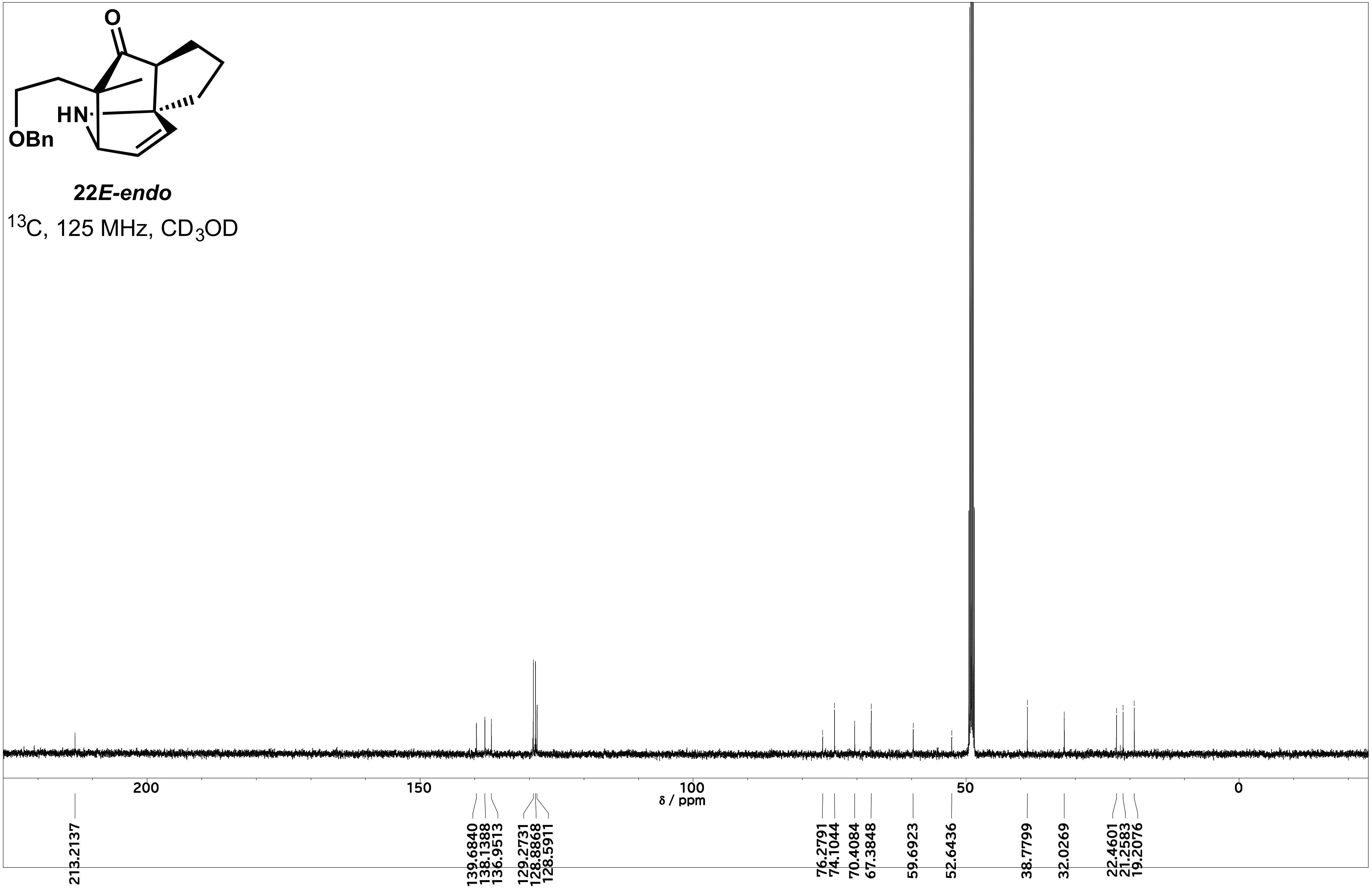
O
HN
OBn
22E-endo
13C, 125 MHz, CD3OD
200
150
100
δ / ppm
50
0
213.2137
139.6840
138.1388
136.9513
129.2731
128.8868
128.5911
76.2791
74.1044
70.4084
67.3848
59.6923
52.6436
38.7799
32.0269
22.4601
21.2583
19.2076

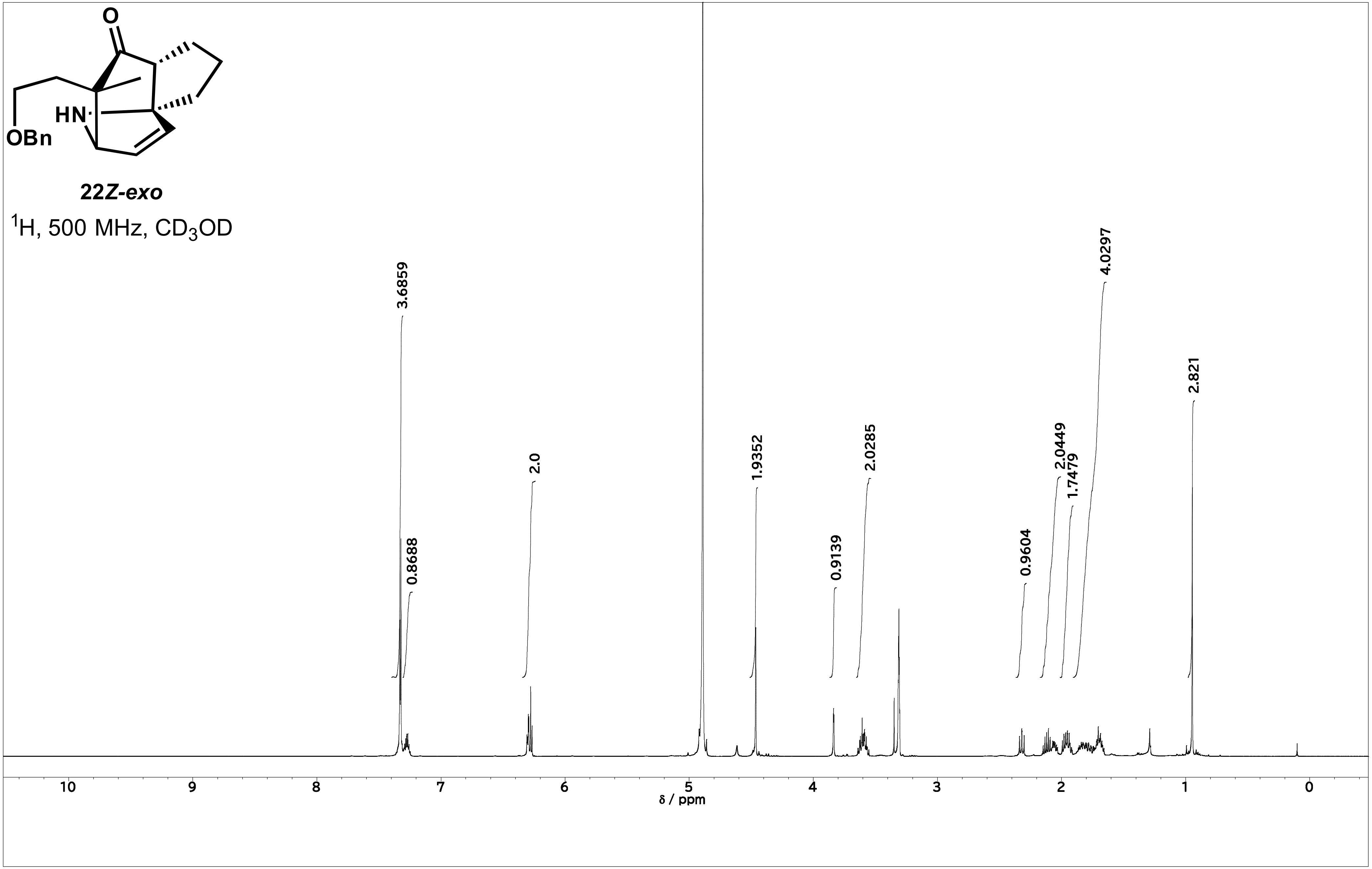
O
HN
OBn
22Z-exo
^{1}H, 500 MHz, CD_3OD
3.6859
0.8688
2.0
1.9352
0.9139
2.0285
0.9604
2.0449
1.7479
4.0297
2.821
10
9
8
7
6
5
4
3
2
1
0
δ / ppm

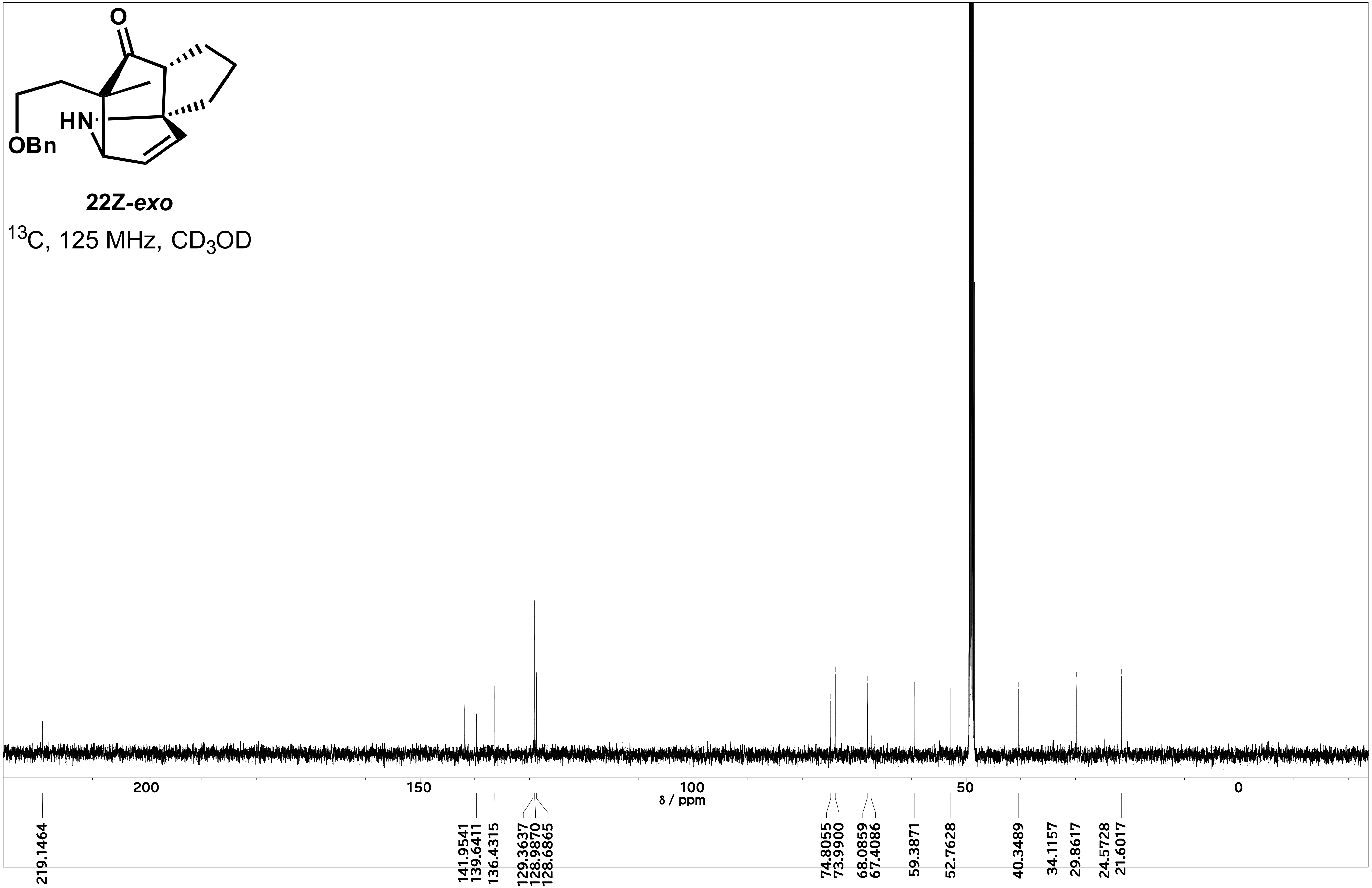
O
HN
OBn
22Z-exo
13C, 125 MHz, CD3OD
200
150
100
50
0
δ / ppm
219.1464
141.9541
139.6411
136.4315
129.3637
128.9870
128.6865
74.8055
73.9900
68.0859
67.4086
59.3871
52.7628
40.3489
34.1157
29.8617
24.5728
21.6017

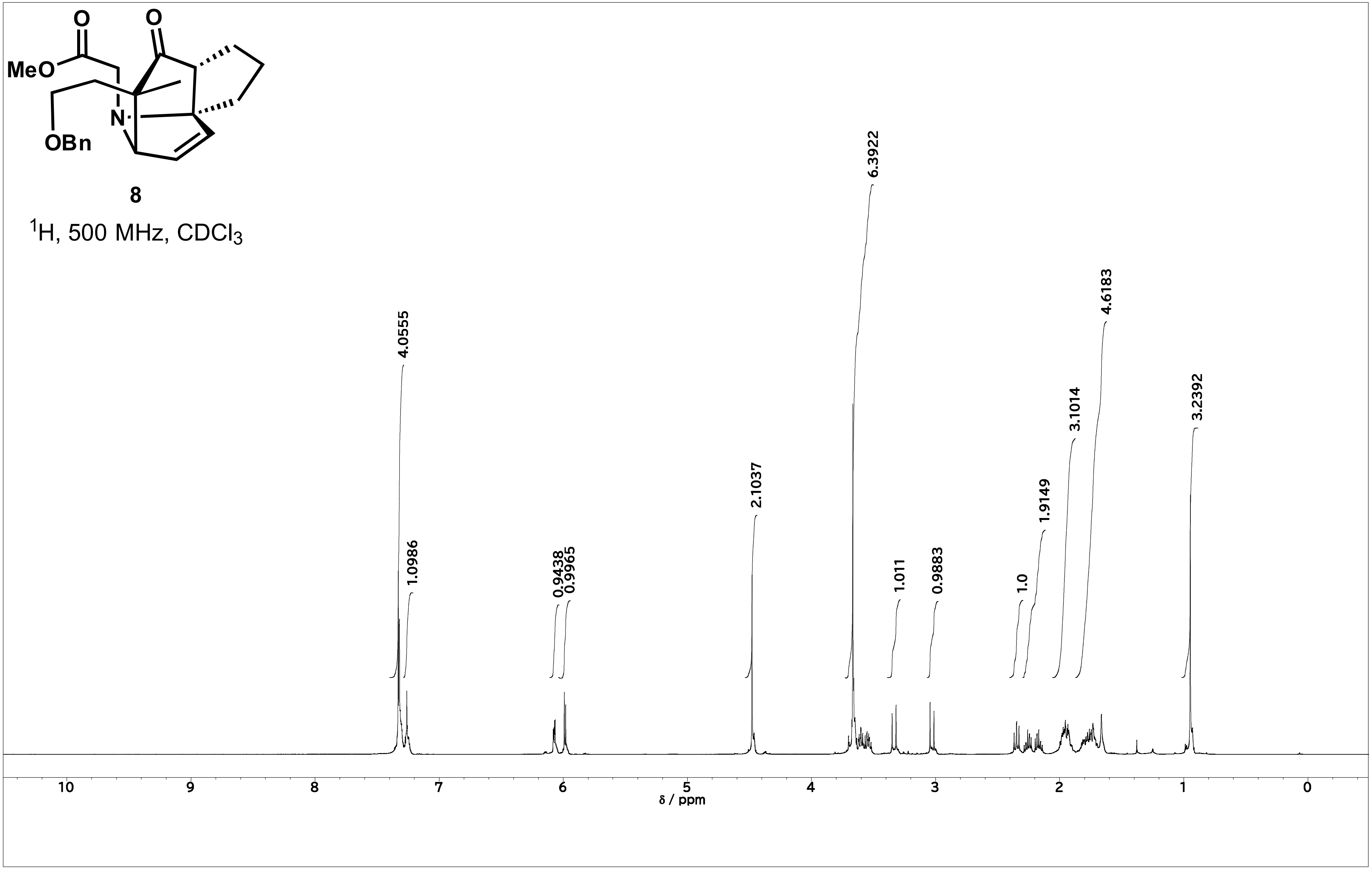

MeO
O
O
N
OBn
8
^{1}H, 500 MHz, CDCl3
4.0555
1.0986
0.9438
0.9965
2.1037
6.3922
1.011
0.9883
1.0
1.9149
3.1014
4.6183
3.2392
10
9
8
7
6
5
4
3
2
1
0
δ / ppm

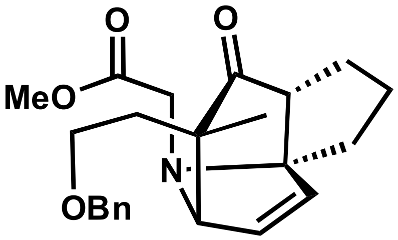

8

^{13}C, 125 MHz, $CDCl_3$

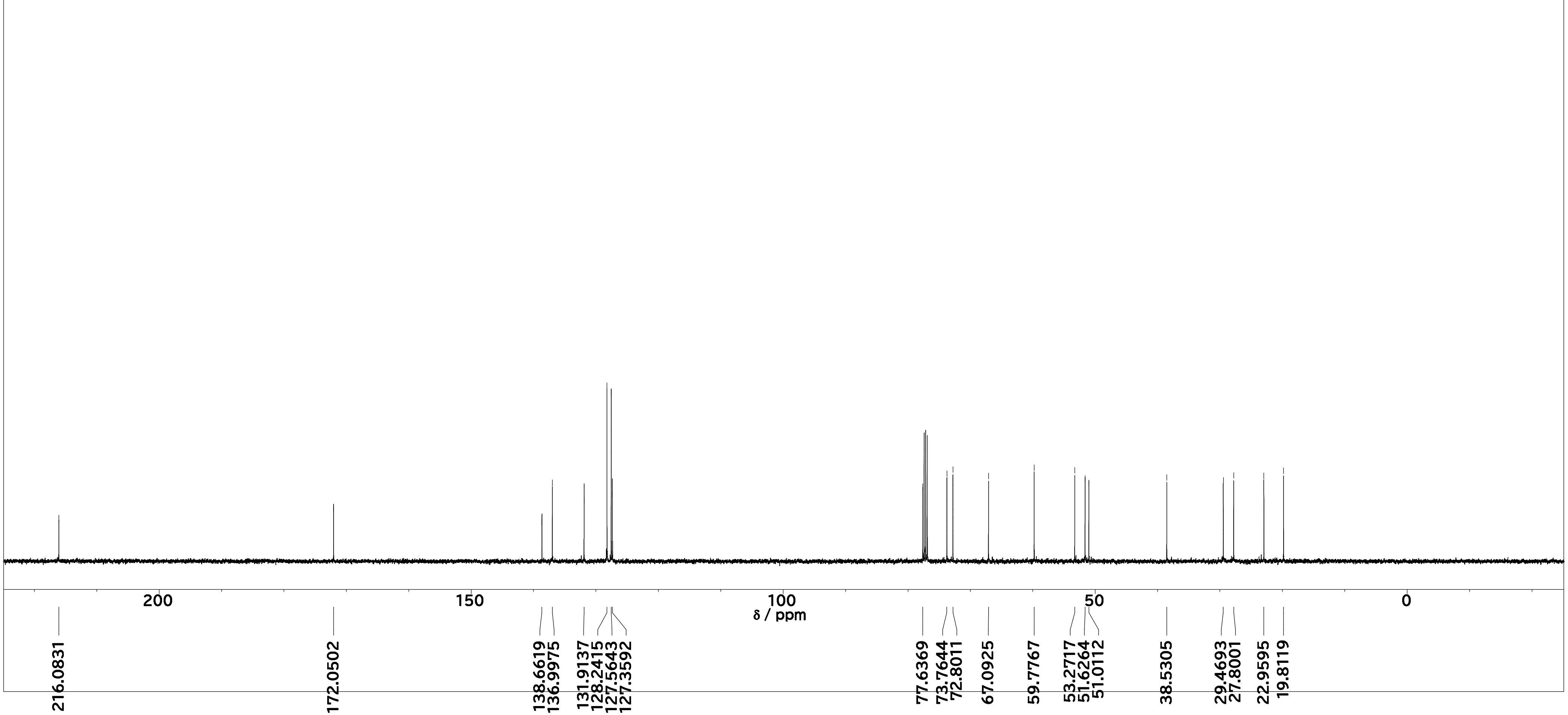

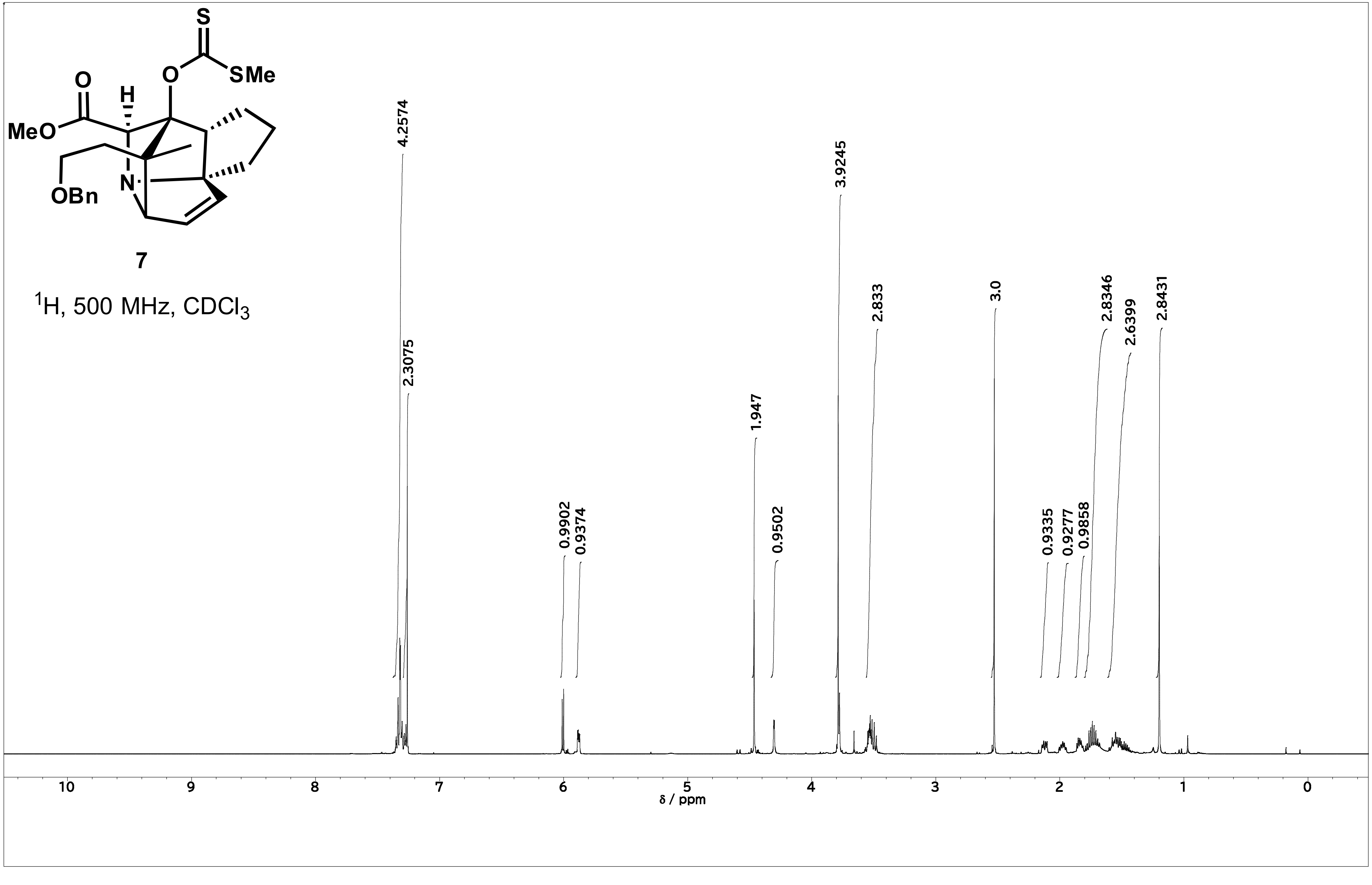
S
SMe
O
MeO
H
N
OBn
7
^1H, 500 MHz, $CDCl_3$
4.2574
2.3075
0.9902
0.9374
1.947
0.9502
3.9245
2.833
3.0
0.9335
0.9277
0.9858
2.8346
2.6399
2.8431
10
9
8
7
6
5
4
3
2
1
0
δ / ppm

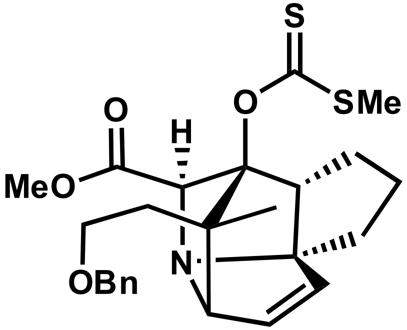

7

^{13}C, 125 MHz, CD_3OD

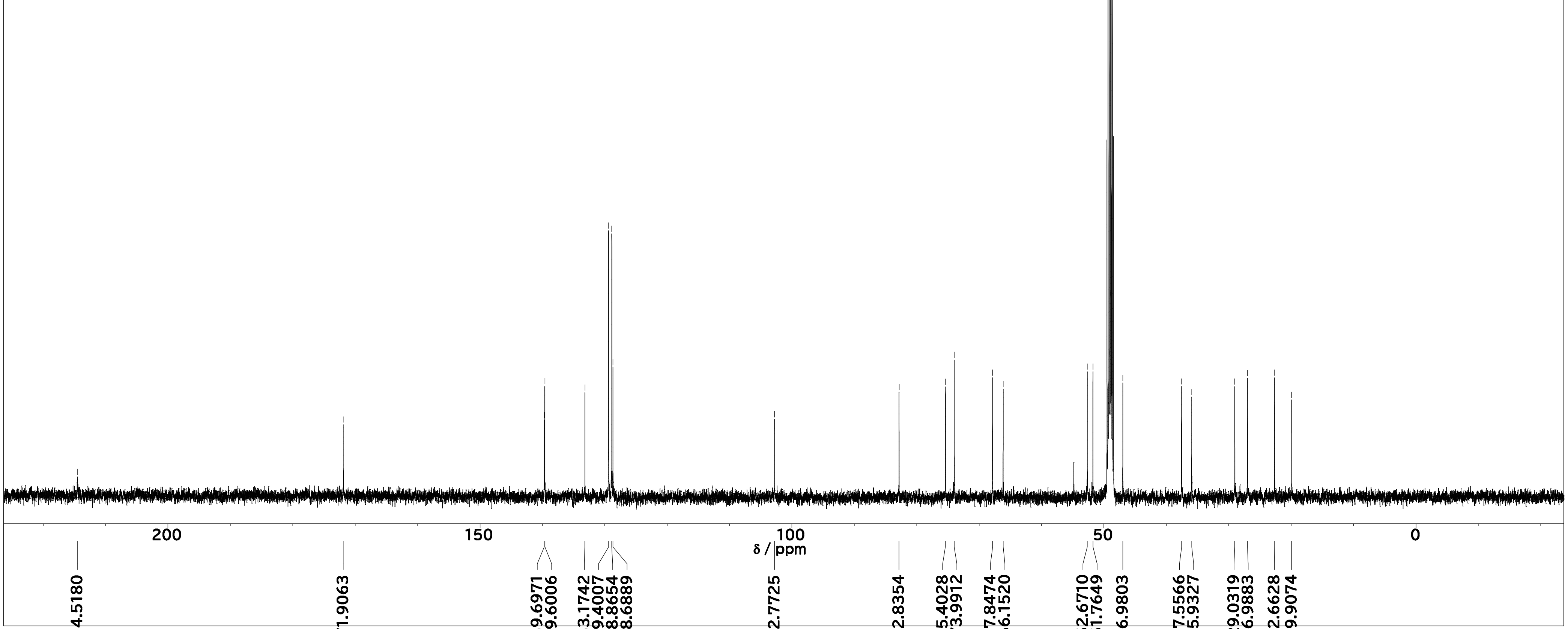

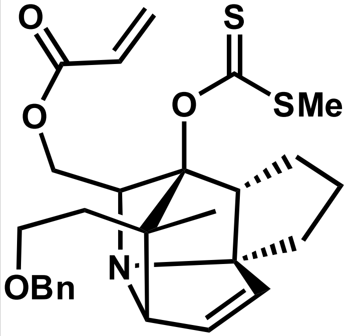

25

^{1}H, 500 MHz, $CDCl_3$

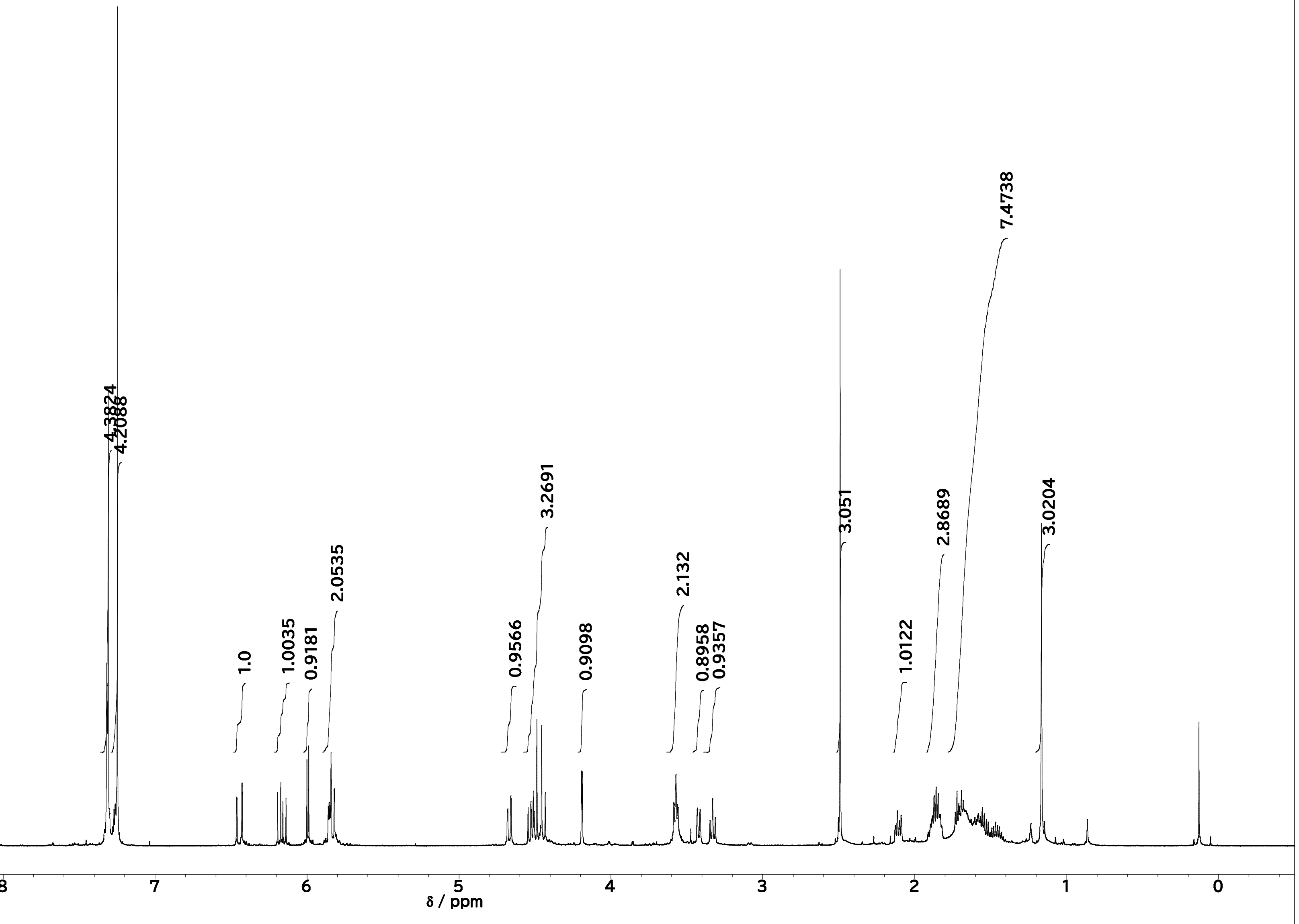

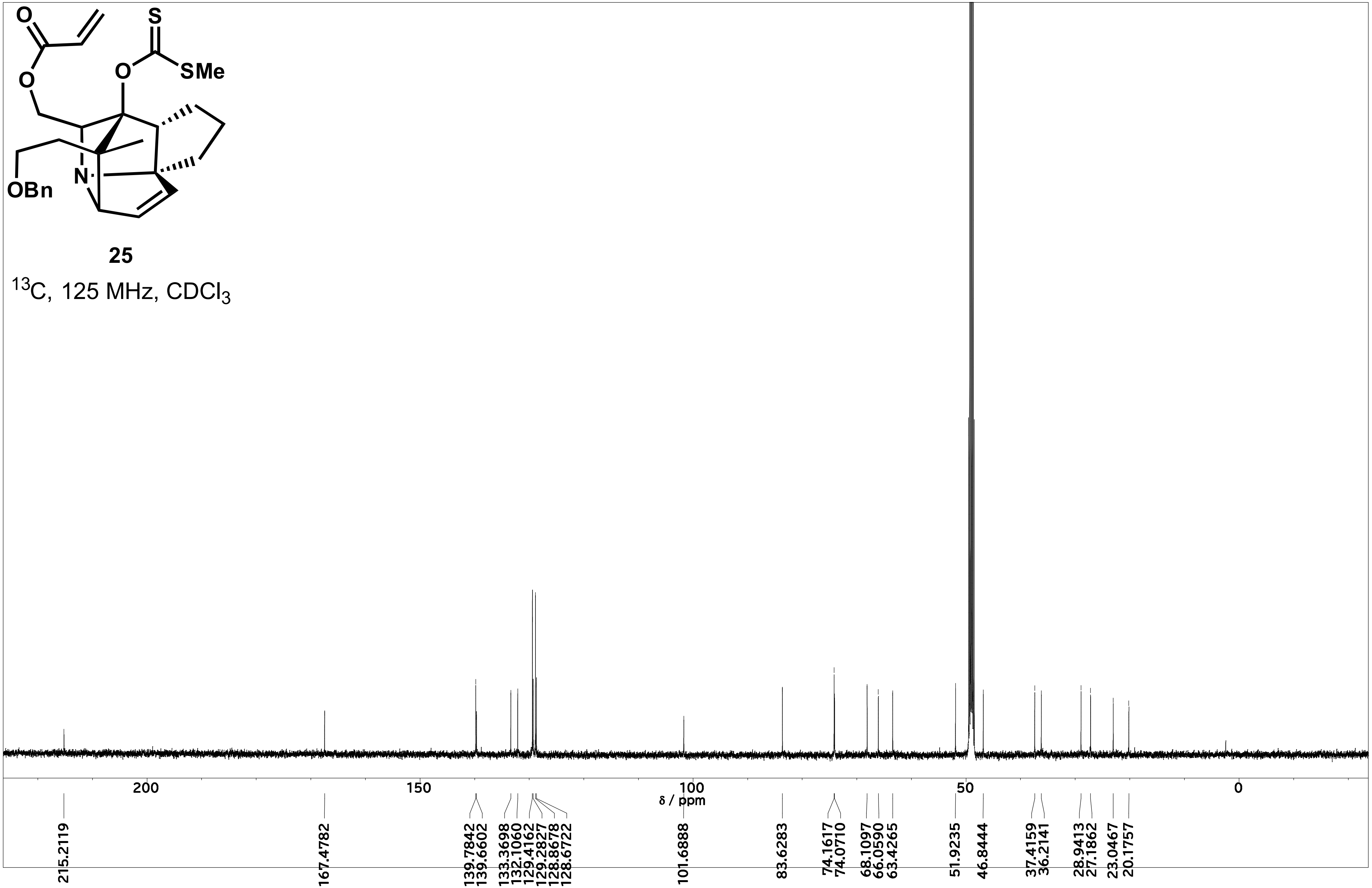

25
^{13}C, 125 MHz, $CDCl_3$
200
150
100
δ / ppm
50
0
215.2119
167.4782
139.7842
139.6602
133.3698
132.1060
129.4162
129.2827
128.8678
128.6722
101.6888
83.6283
74.1617
74.0710
68.1097
66.0590
63.4265
51.9235
46.8444
37.4159
36.2141
28.9413
27.1862
23.0467
20.1757

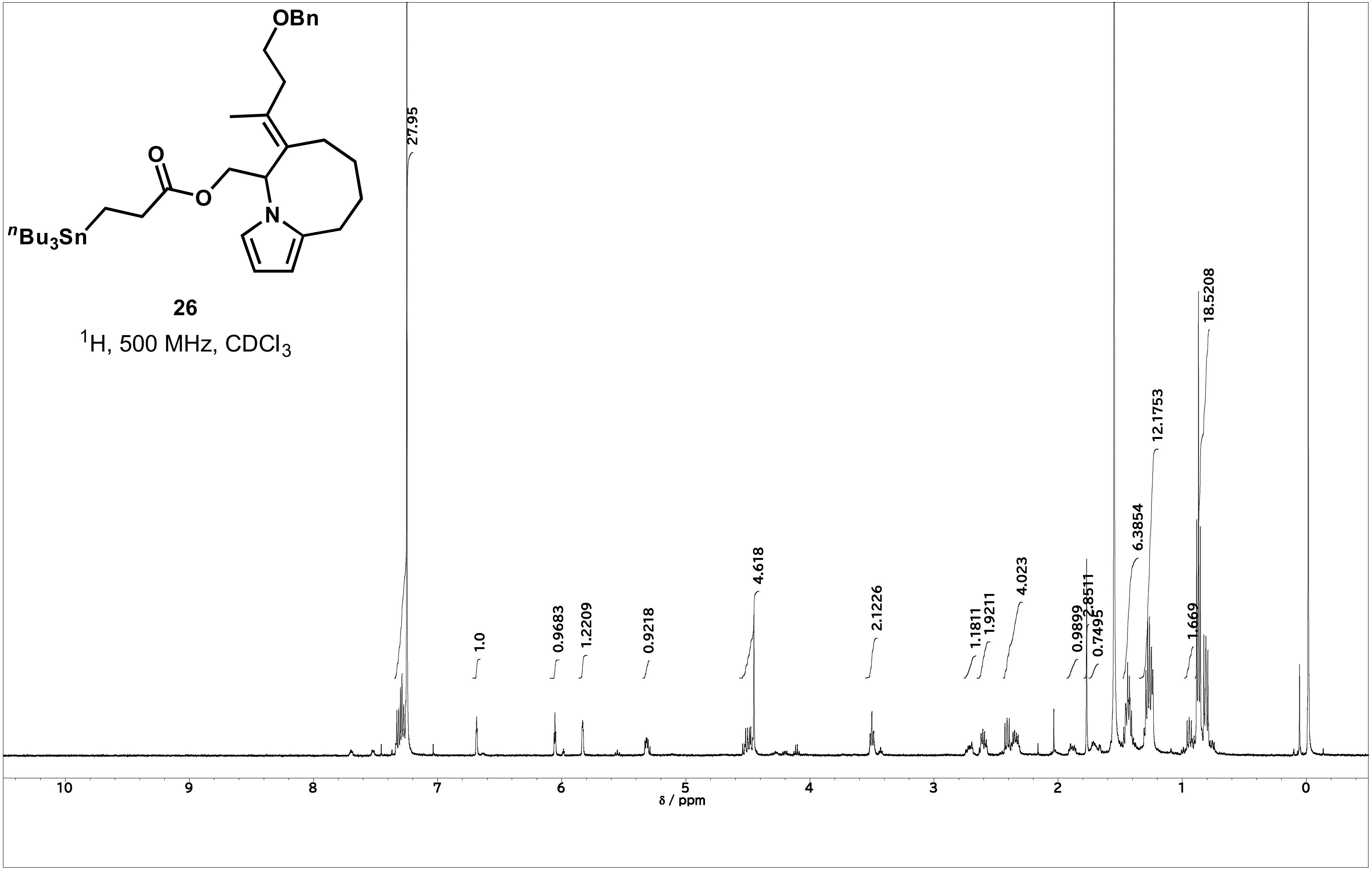
OBn
O
O
N
^{n}Bu$_3$Sn
26
^{1}H, 500 MHz, CDCl$_3$
27.95
1.0
0.9683
1.2209
0.9218
4.618
2.1226
1.1811
1.9211
4.023
0.9899
2.8511
0.7495
6.3854
12.1753
1.669
18.5208
10
9
8
7
6
5
4
3
2
1
0
δ / ppm

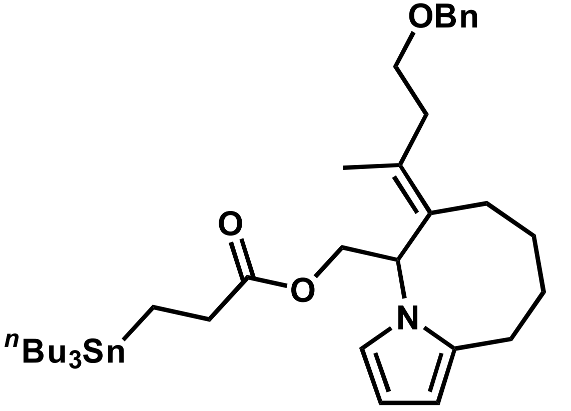

26

^{13}C, 125 MHz, $CDCl_3$

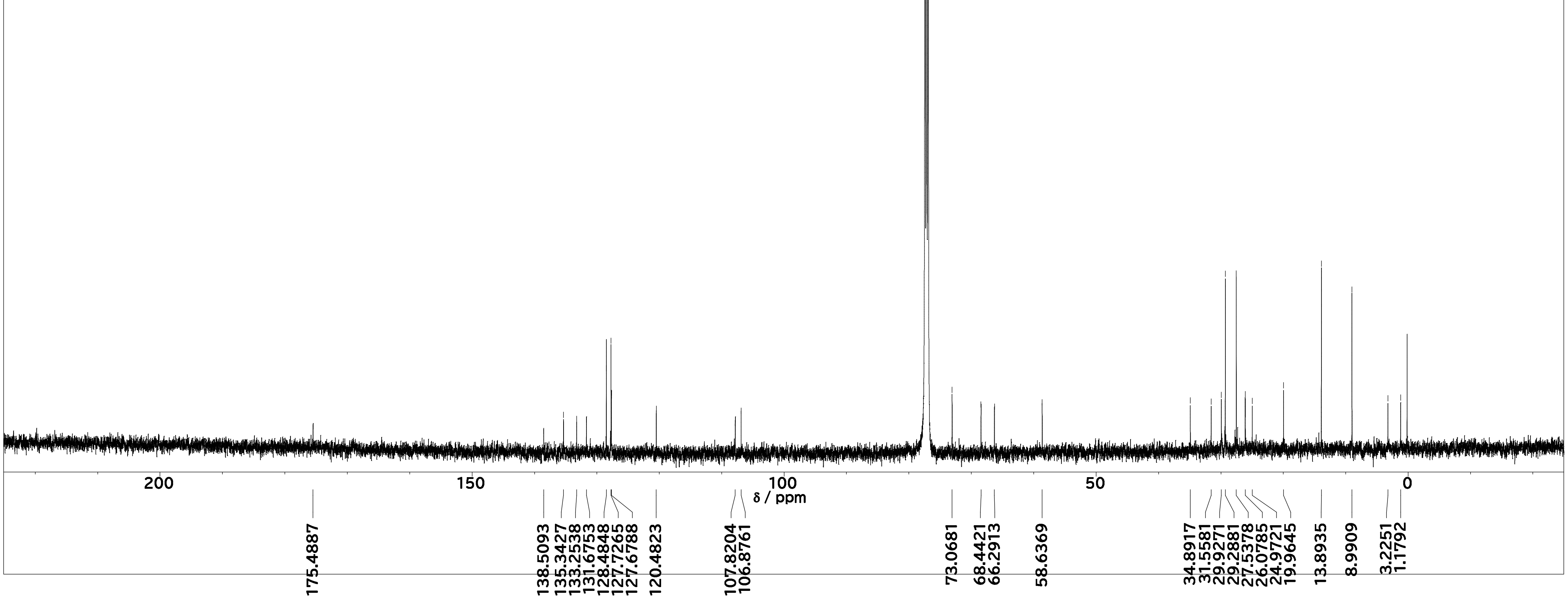

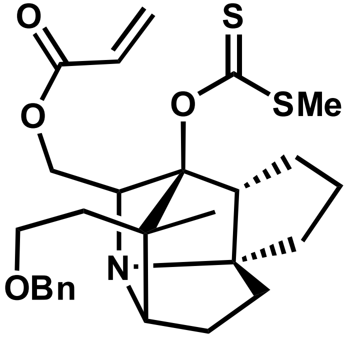

27

^1H, 500 MHz, $CDCl_3$

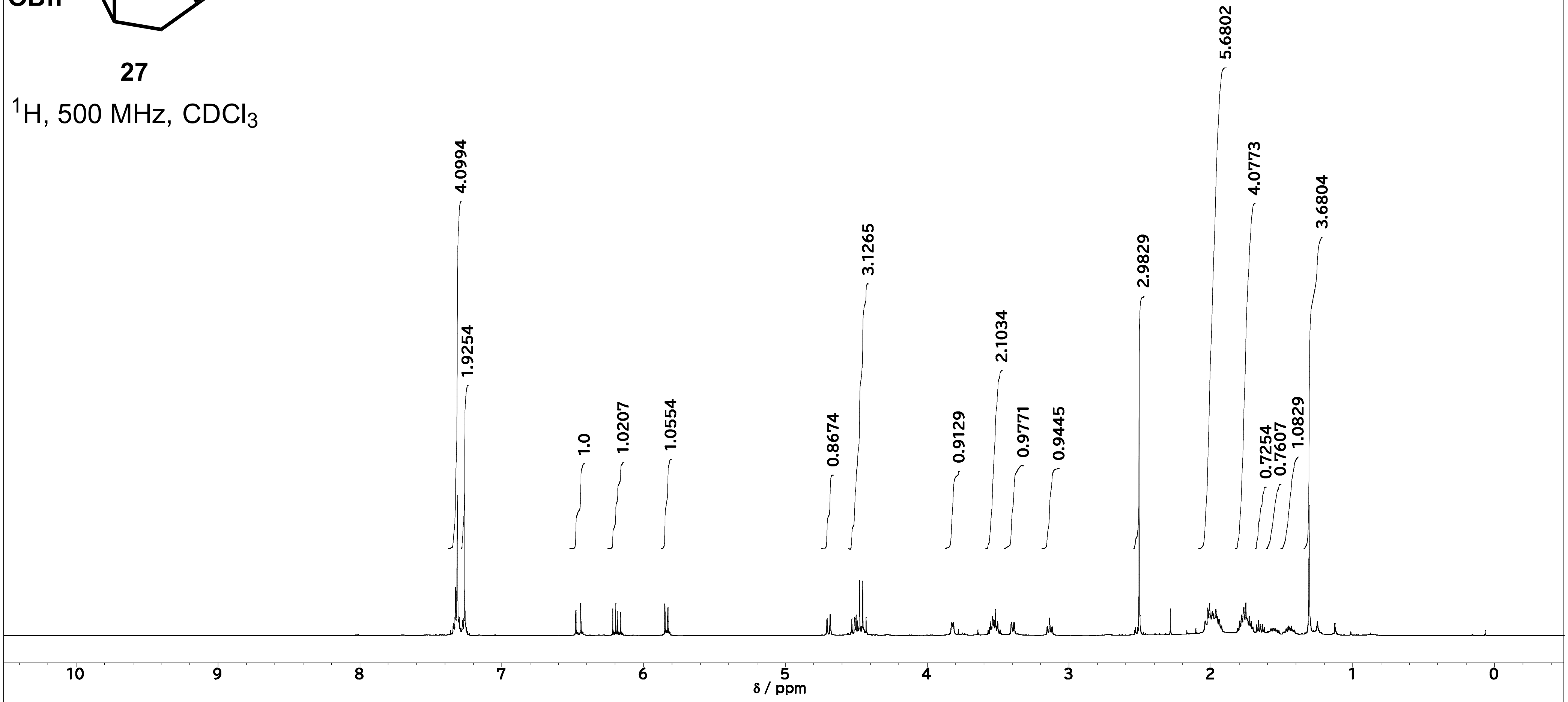

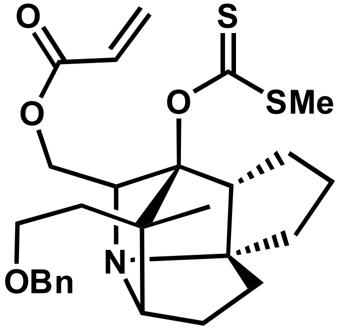

27

^{13}C, 125 MHz, $CDCl_3$

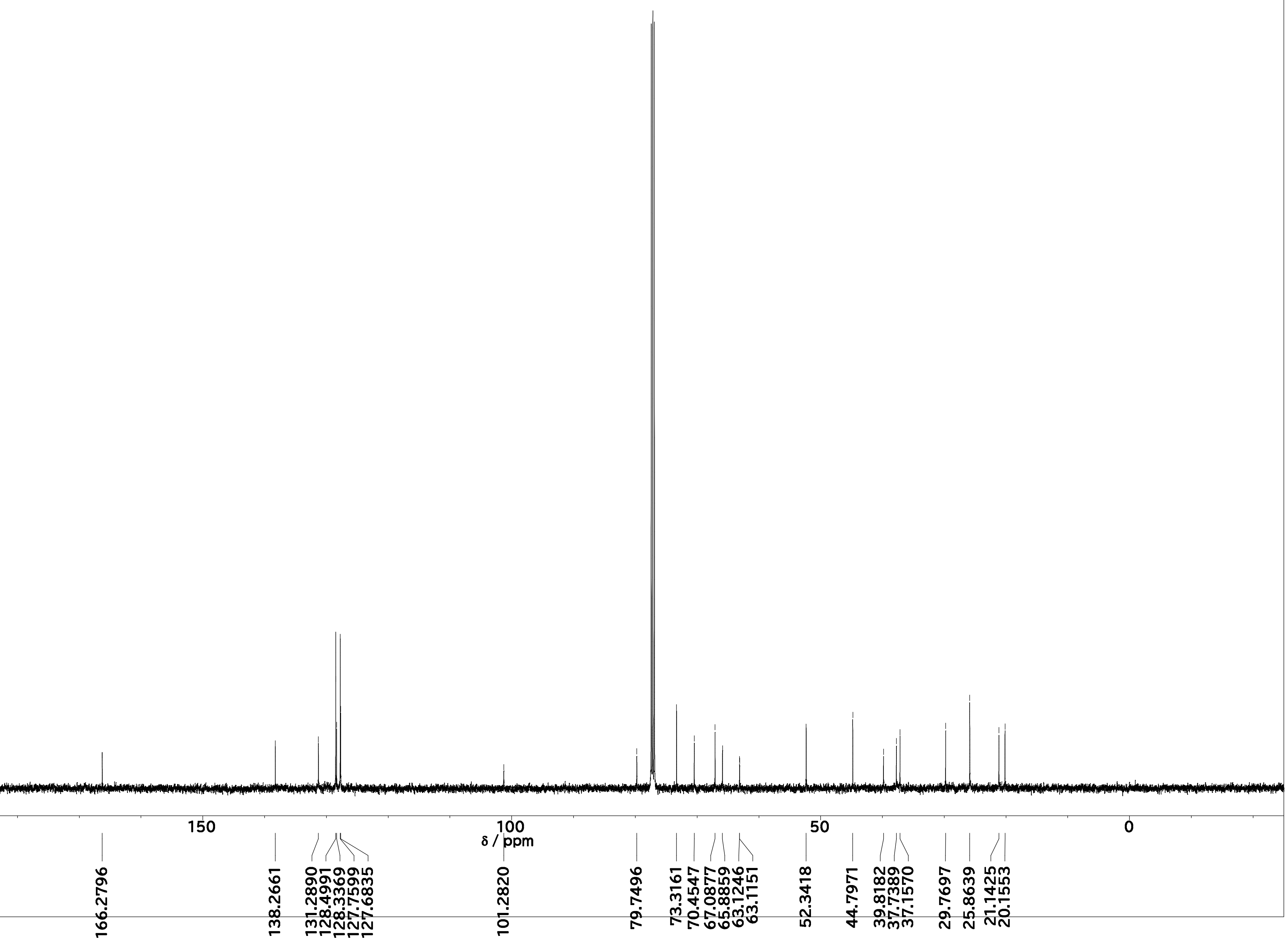

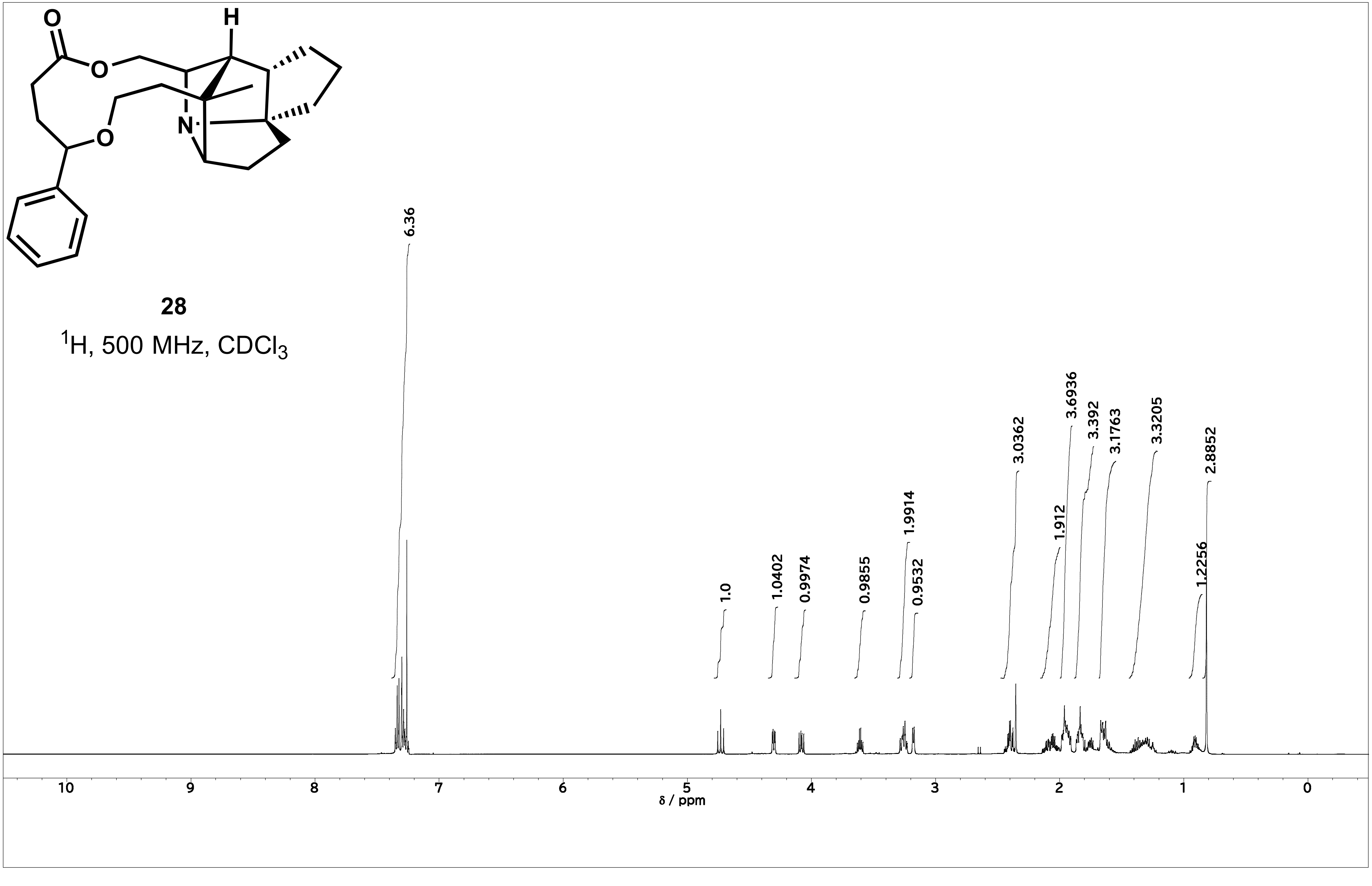

28
^{1}H, 500 MHz, $CDCl_3$
6.36
1.0
1.0402
0.9974
0.9855
1.9914
0.9532
3.0362
1.912
3.6936
3.392
3.1763
3.3205
1.2256
2.8852
10
9
8
7
6
5
4
3
2
1
0
δ / ppm

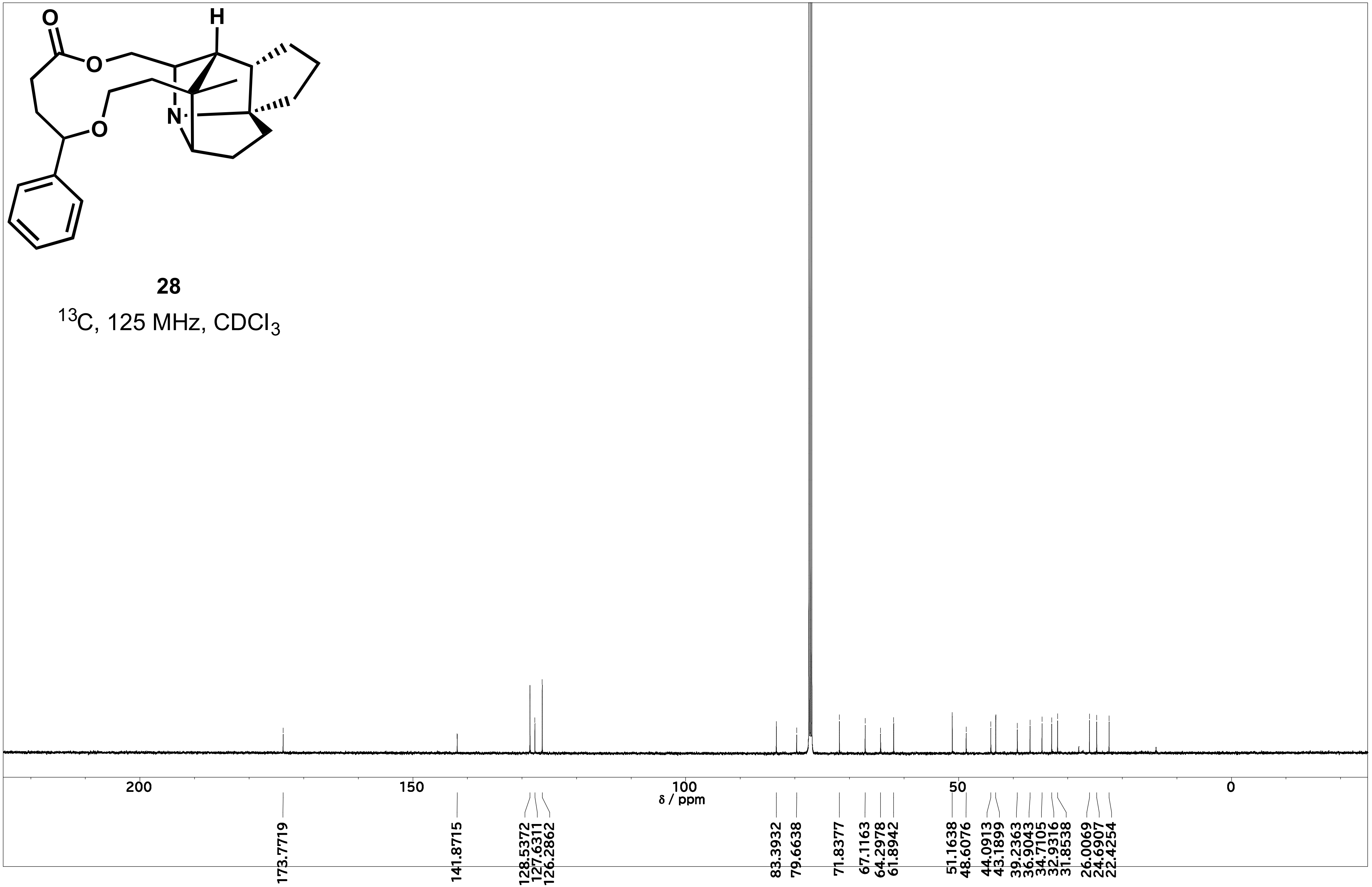
H
O
O
O
N
28
^{13}C, 125 MHz, CDCl$_3$
200
150
100
50
0
δ / ppm
173.7719
141.8715
128.5372
127.6311
126.2862
83.3932
79.6638
71.8377
67.1163
64.2978
61.8942
51.1638
48.6076
44.0913
43.1899
39.2363
36.9043
34.7105
32.9316
31.8538
26.0069
24.6907
22.4254

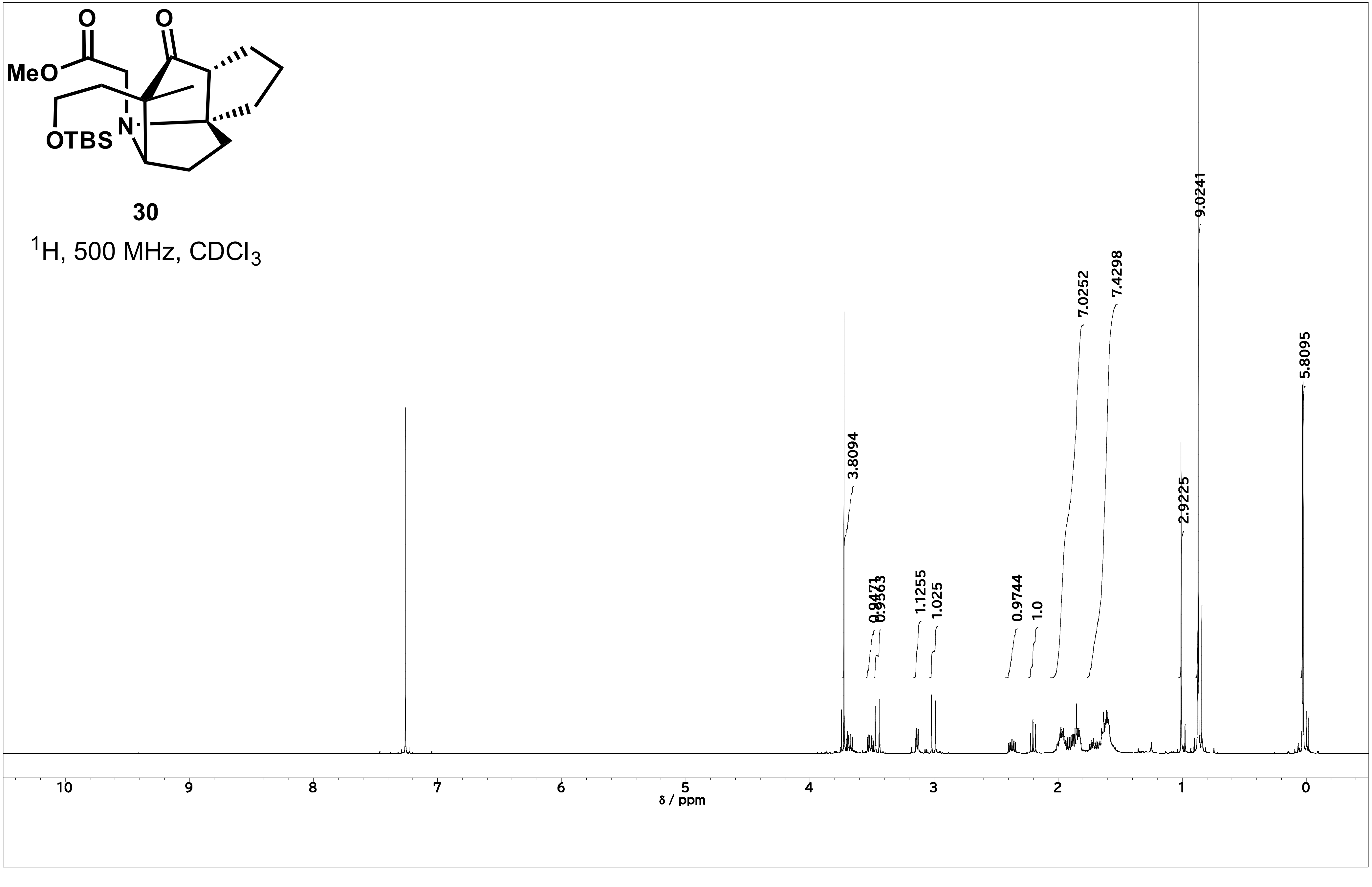
MeO
O
O
OTBS
N
30
^{1}H, 500 MHz, CDCl$_3$
3.8094
0.9471
0.9563
1.1255
1.025
0.9744
1.0
7.0252
7.4298
2.9225
9.0241
5.8095
10
9
8
7
6
5
4
3
2
1
0
δ / ppm

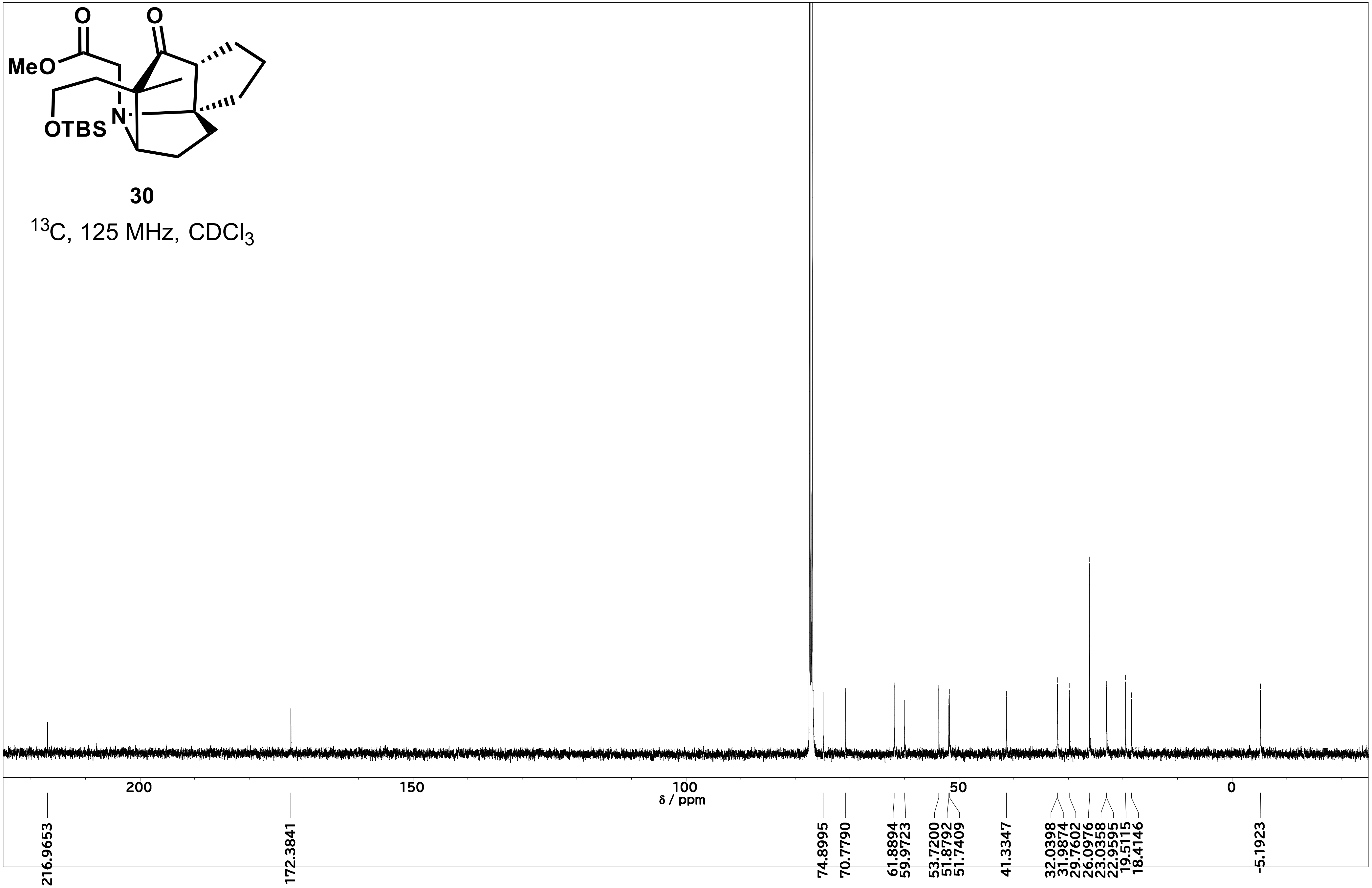
MeO
O
O
OTBS
N
30
^{13}C, 125 MHz, CDCl$_3$
200
150
100
δ / ppm
50
0
216.9653
172.3841
74.8995
70.7790
61.8894
59.9723
53.7200
51.8792
51.7409
41.3347
32.0398
31.9874
29.7602
26.0976
23.0358
22.9595
19.5115
18.4146
-5.1923

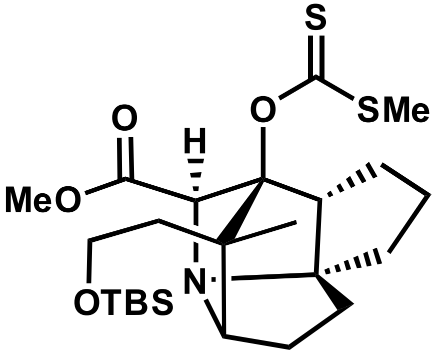

31

^1H, 500 MHz, $CDCl_3$

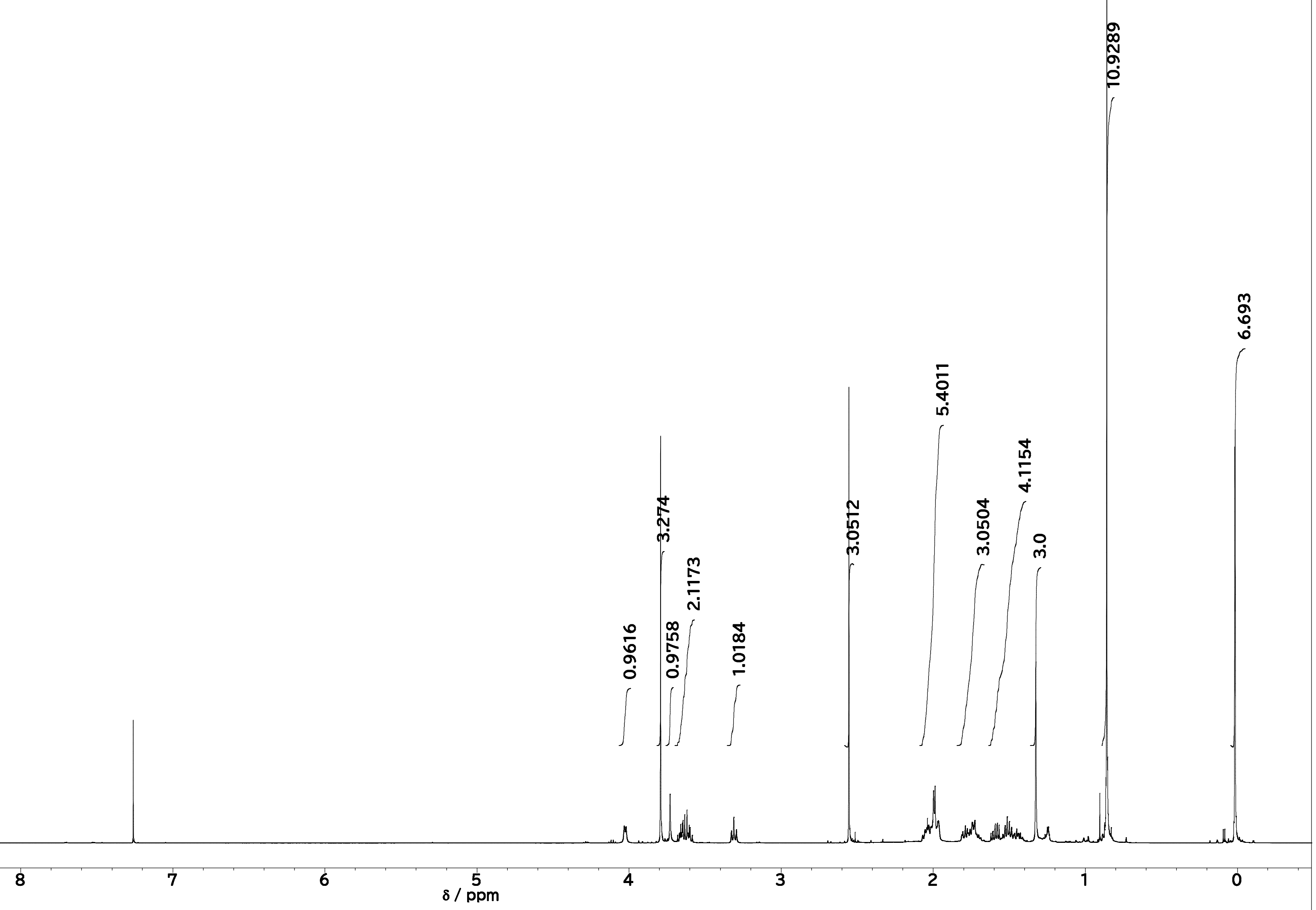

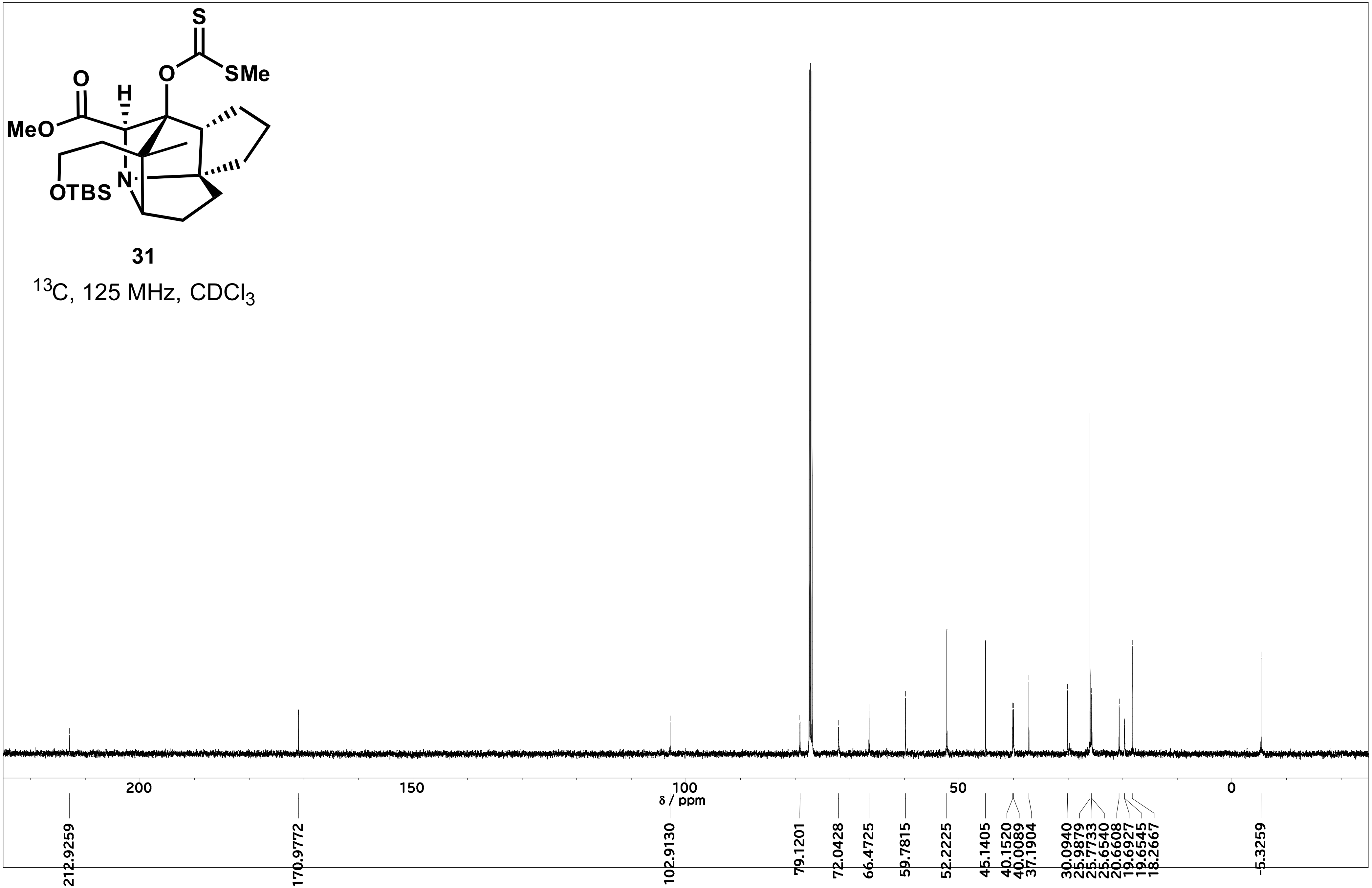
S
O
SMe
O
H
MeO
N
OTBS
31
^{13}C, 125 MHz, CDCl$_3$
200
150
100
50
0
δ / ppm
212.9259
170.9772
102.9130
79.1201
72.0428
66.4725
59.7815
52.2225
45.1405
40.1520
40.0089
37.1904
30.0940
25.9879
25.7733
25.6540
20.6608
19.6927
19.6545
18.2667
-5.3259

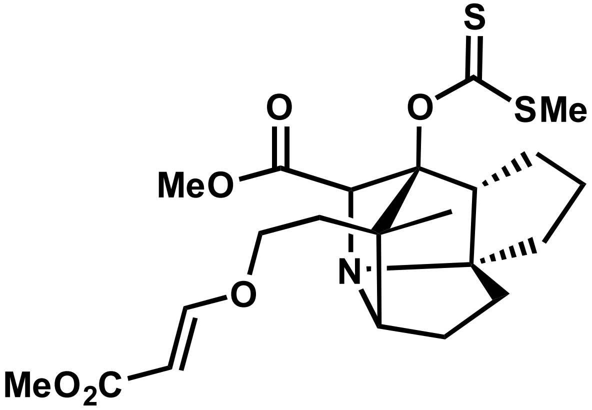

32

^1H, 500 MHz, $CDCl_3$

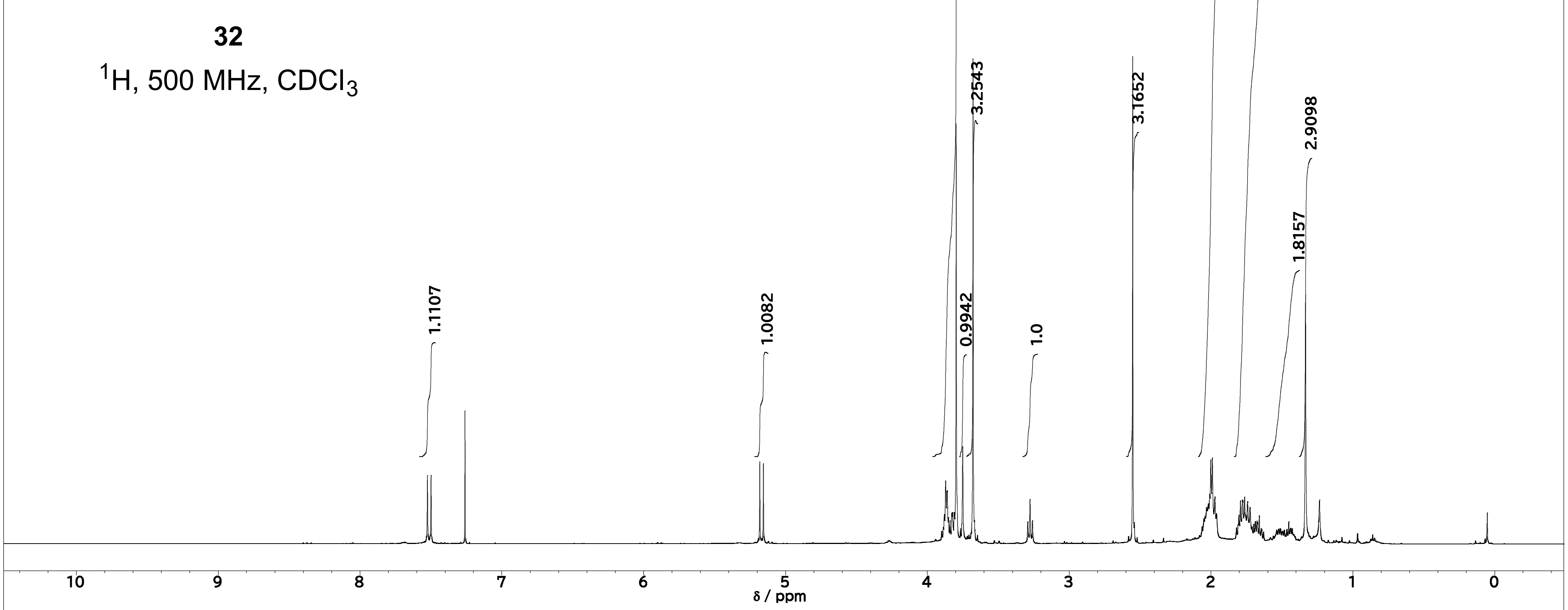

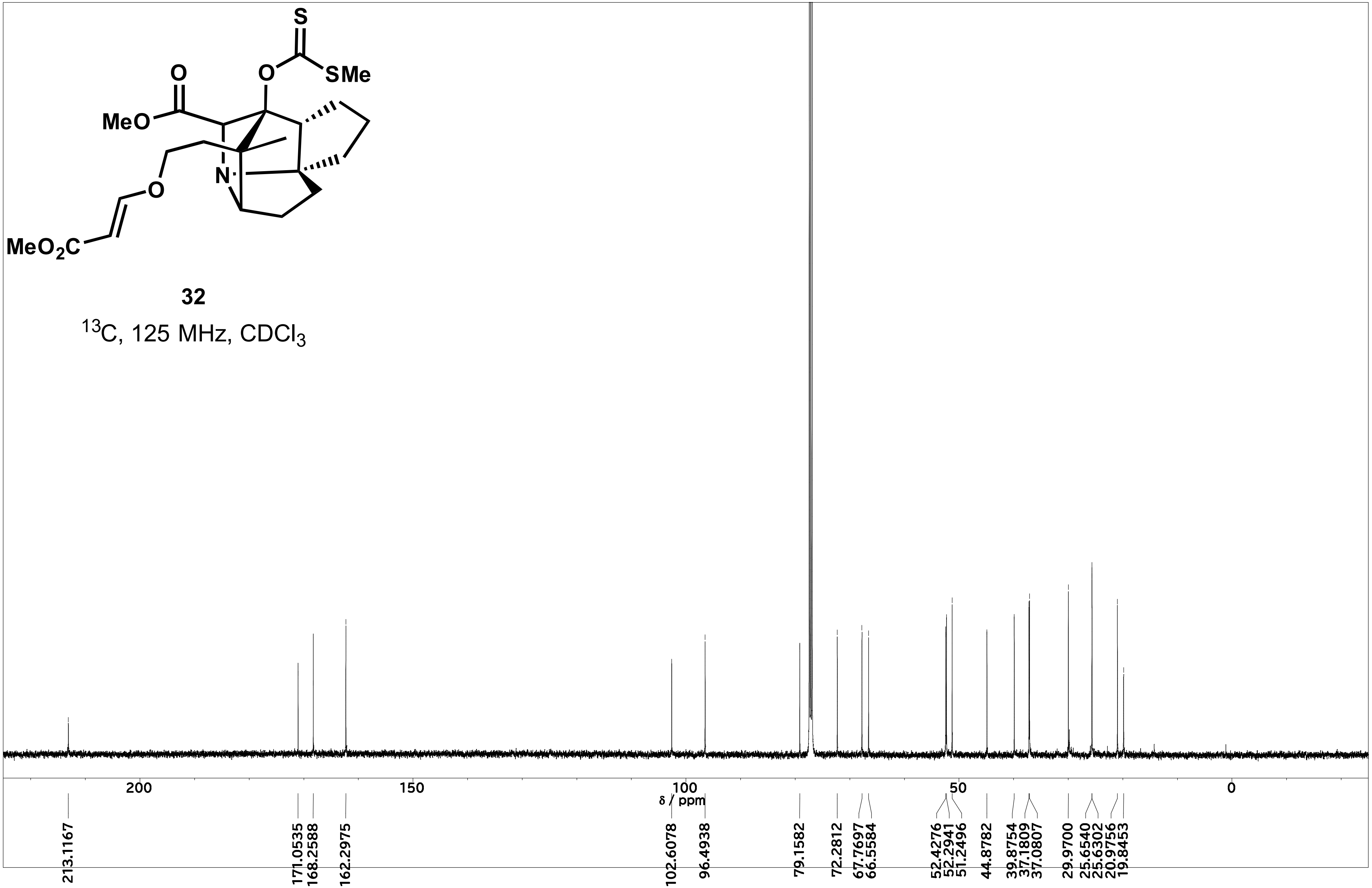
32
^{13}C, 125 MHz, $CDCl_3$
200
150
100
δ / ppm
50
0
213.1167
171.0535
168.2588
162.2975
102.6078
96.4938
79.1582
72.2812
67.7697
66.5584
52.4276
52.2941
51.2496
44.8782
39.8754
37.1809
37.0807
29.9700
25.6540
25.6302
20.9756
19.8453

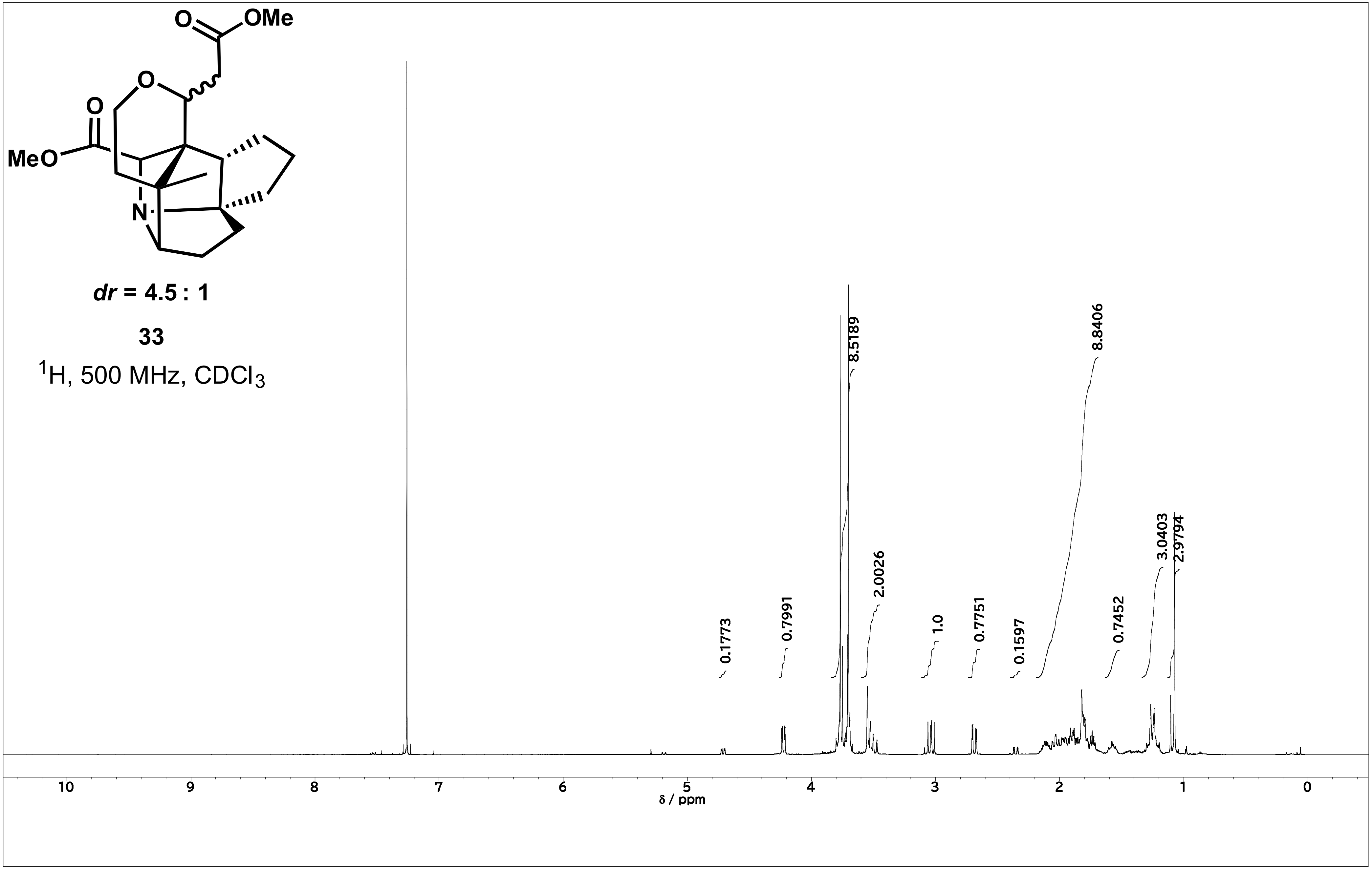
OMe
O
MeO
N
dr = 4.5 : 1
33
^{1}H, 500 MHz, $CDCl_3$
0.1773
0.7991
8.5189
2.0026
1.0
0.7751
0.1597
8.8406
0.7452
3.0403
2.9794
10
9
8
7
6
5
4
3
2
1
0
δ / ppm

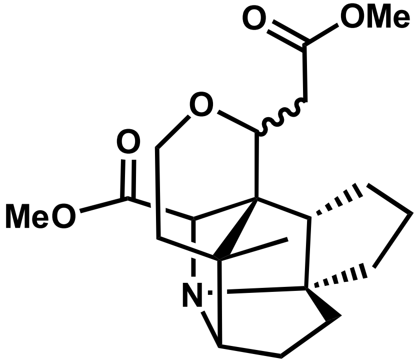

dr = 4.5 : 1

33

^{13}C, 125 MHz, $CDCl_3$

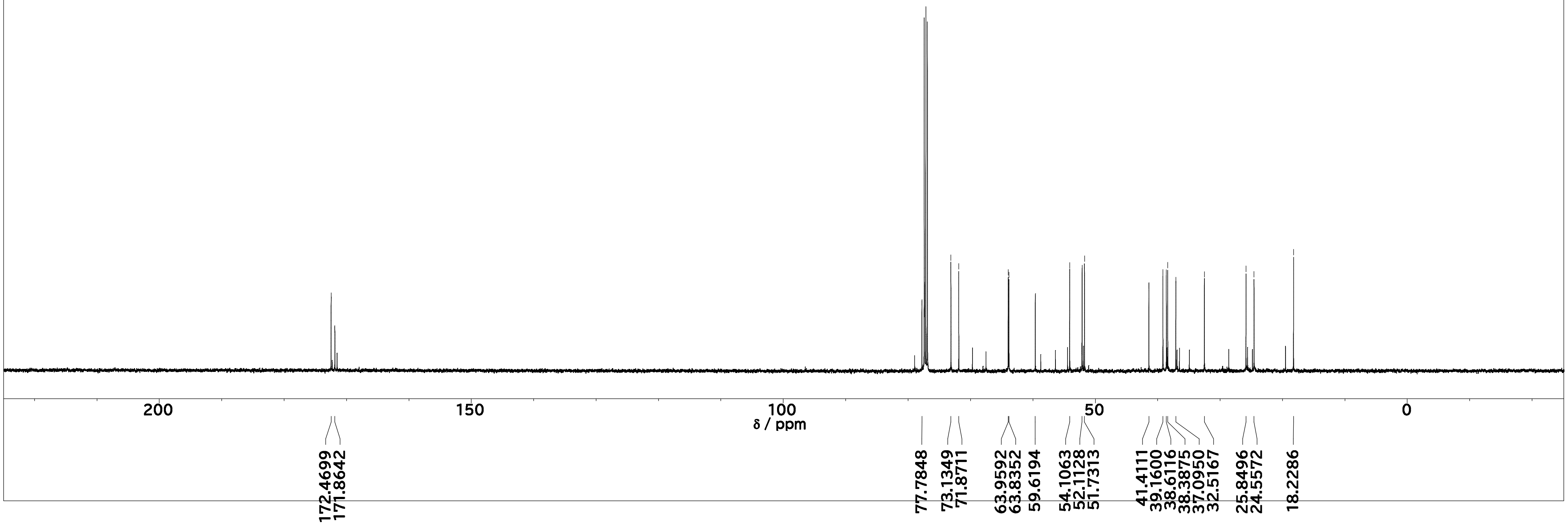

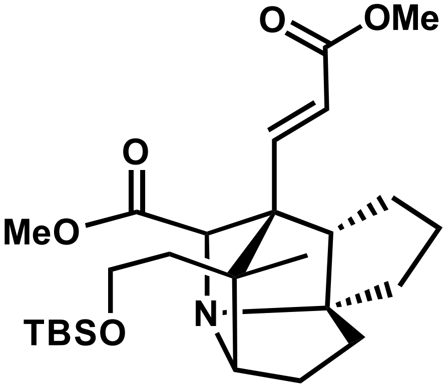

34

^{1}H, 500 MHz, $CDCl_3$

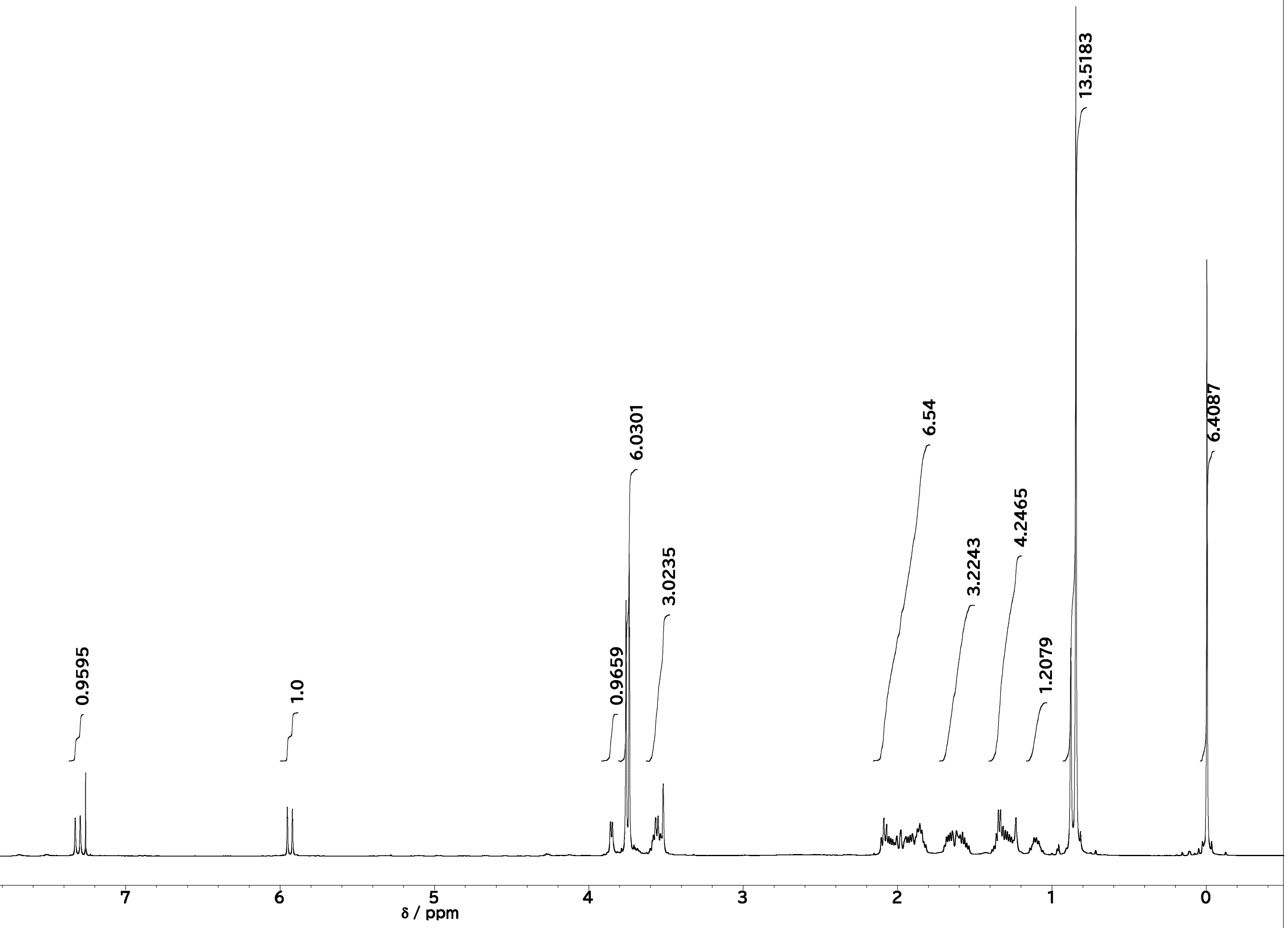

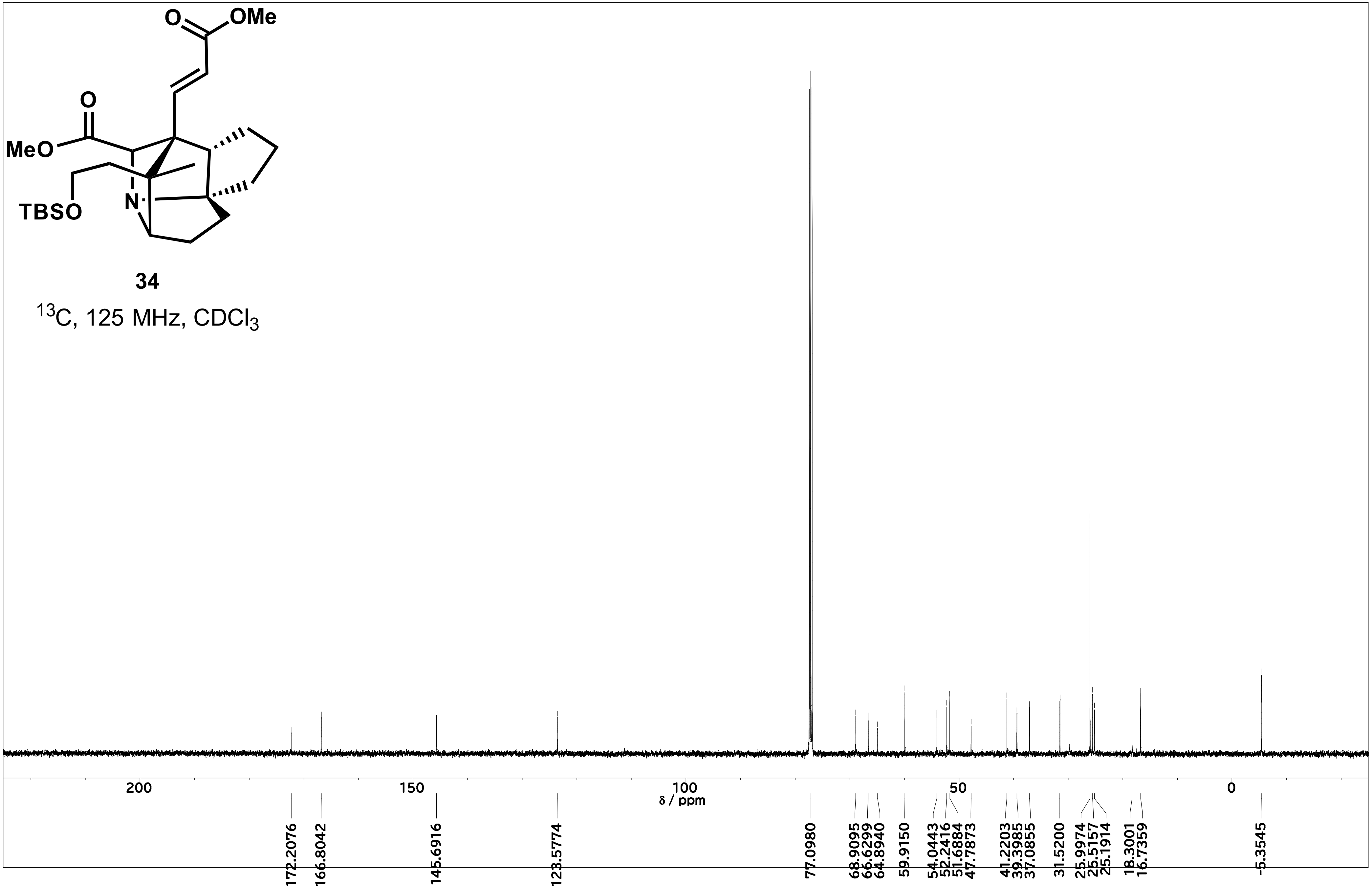
O
OMe
O
MeO
TBSO
N
34
13C, 125 MHz, CDCl3
200
150
100
δ / ppm
50
0
172.2076
166.8042
145.6916
123.5774
77.0980
68.9095
66.6299
64.8940
59.9150
54.0443
52.2416
51.6884
47.7873
41.2203
39.3985
37.0855
31.5200
25.9974
25.5157
25.1914
18.3001
16.7359
-5.3545

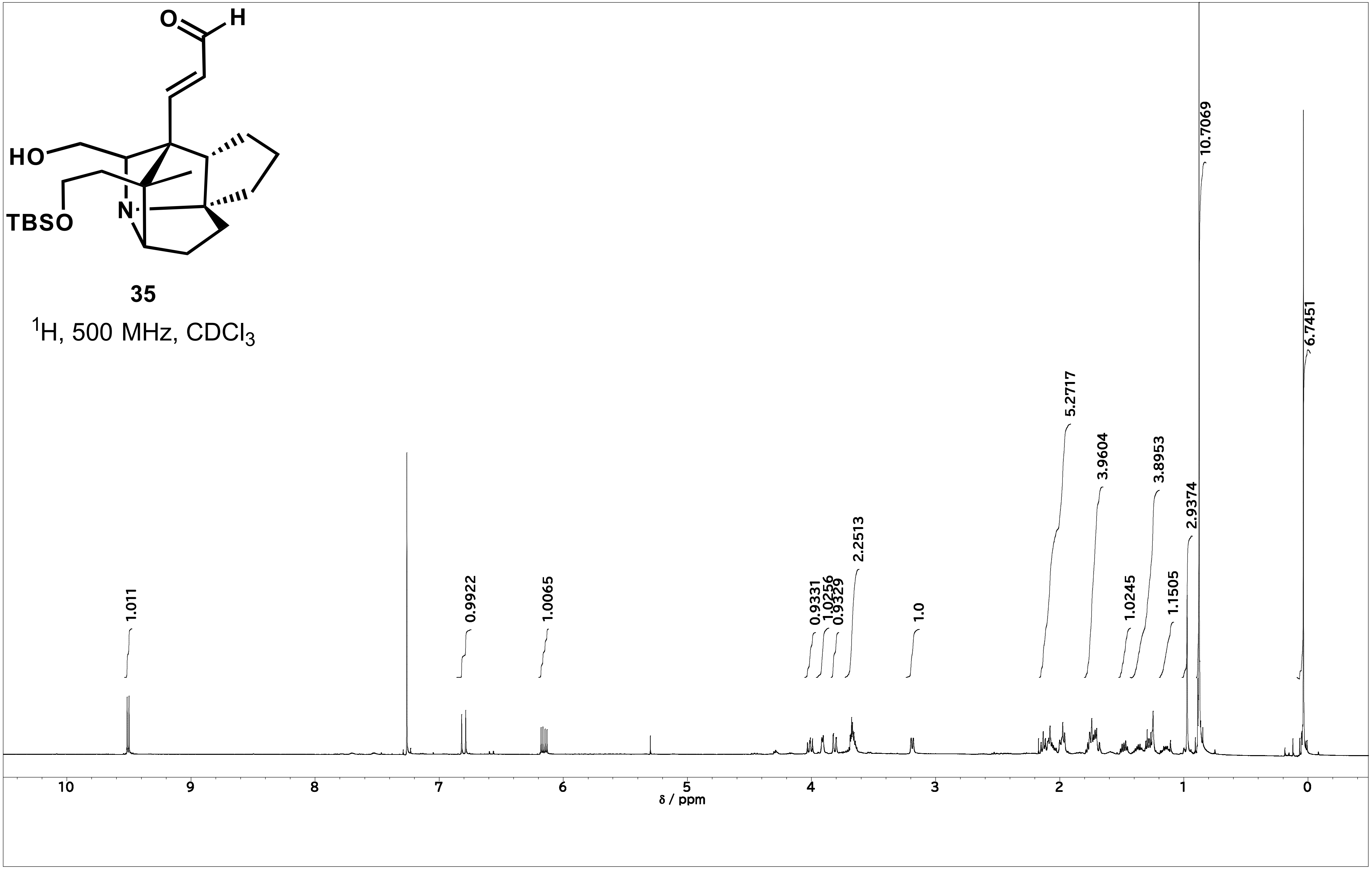

O
H
HO
TBSO
N
35
1H, 500 MHz, CDCl3
1.011
0.9922
1.0065
0.9331
1.0256
0.9329
2.2513
1.0
5.2717
3.9604
1.0245
3.8953
1.1505
2.9374
10.7069
6.7451
10
9
8
7
6
5
4
3
2
1
0
δ / ppm

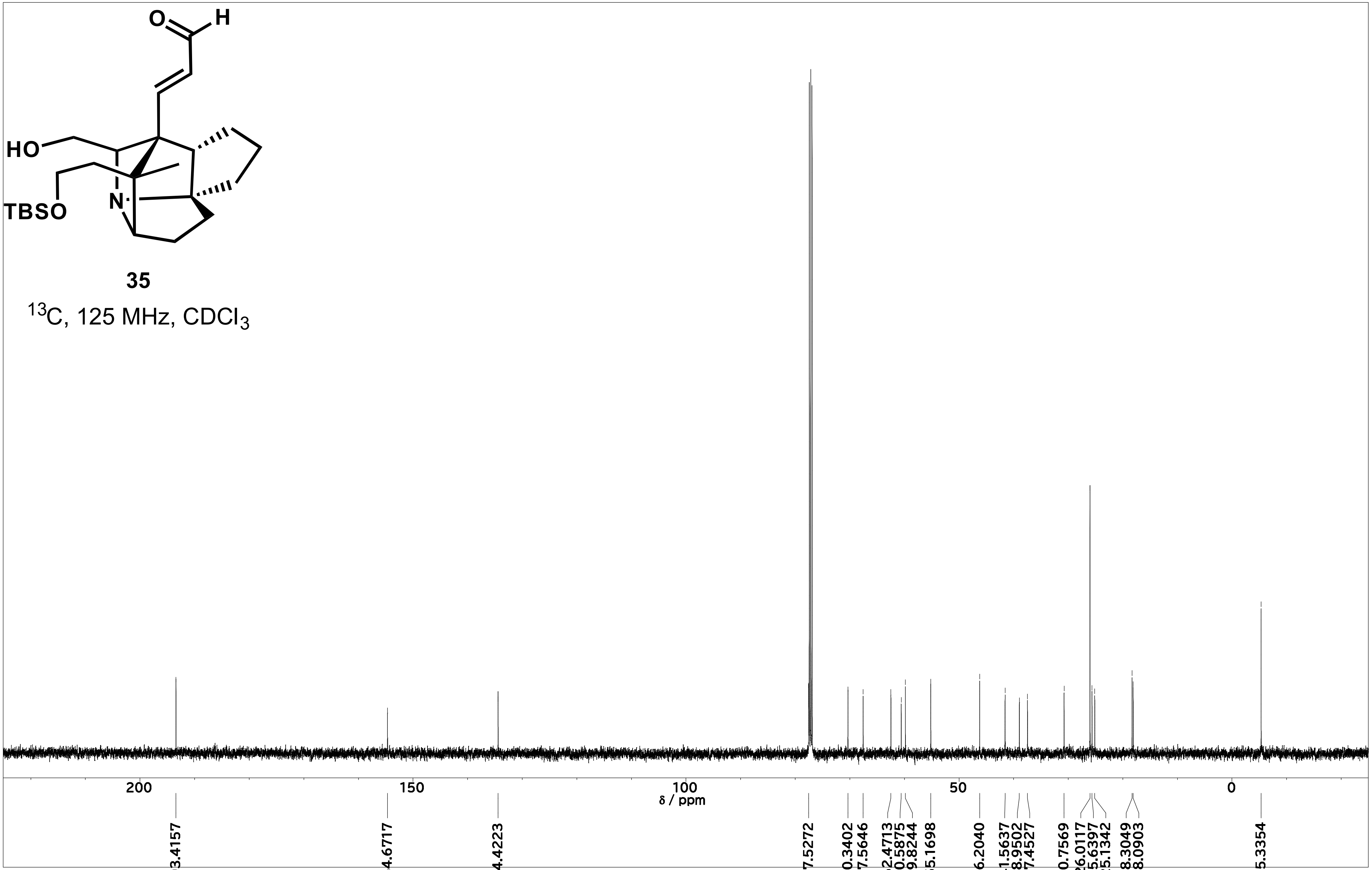

O
H
HO
TBSO
N
35
^{13}C, 125 MHz, $CDCl_3$
200
150
100
50
0
δ / ppm
193.4157
154.6717
134.4223
77.5272
70.3402
67.5646
62.4713
60.5875
59.8244
55.1698
46.2040
41.5637
38.9502
37.4527
30.7569
26.0117
25.6397
25.1342
18.3049
18.0903
-5.3354

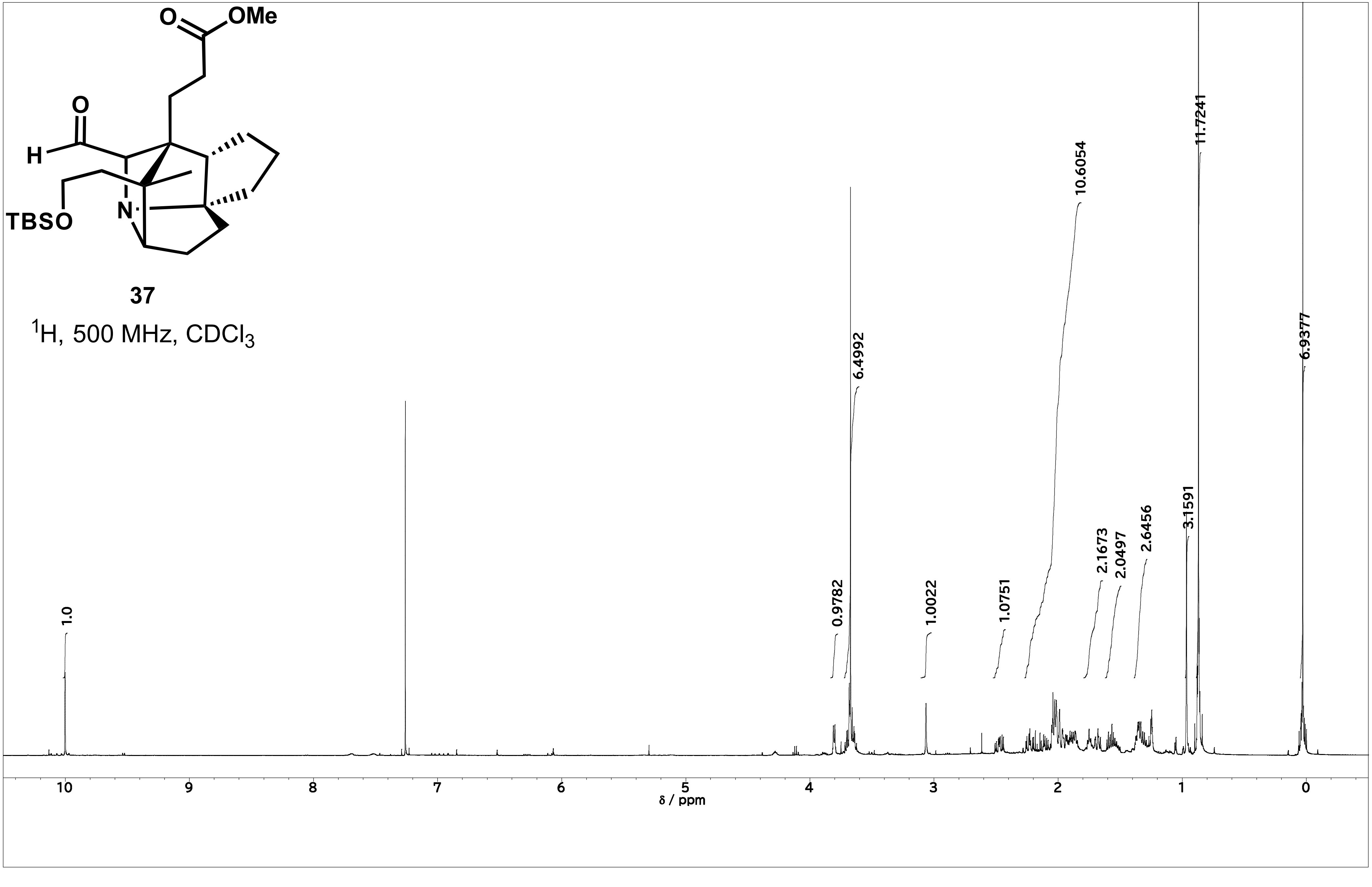

O
OMe
O
H
TBSO
N
37
^{1}H, 500 MHz, $CDCl_3$
1.0
0.9782
6.4992
1.0022
1.0751
10.6054
2.1673
2.0497
2.6456
3.1591
11.7241
6.9377
10
9
8
7
6
5
4
3
2
1
0
δ / ppm

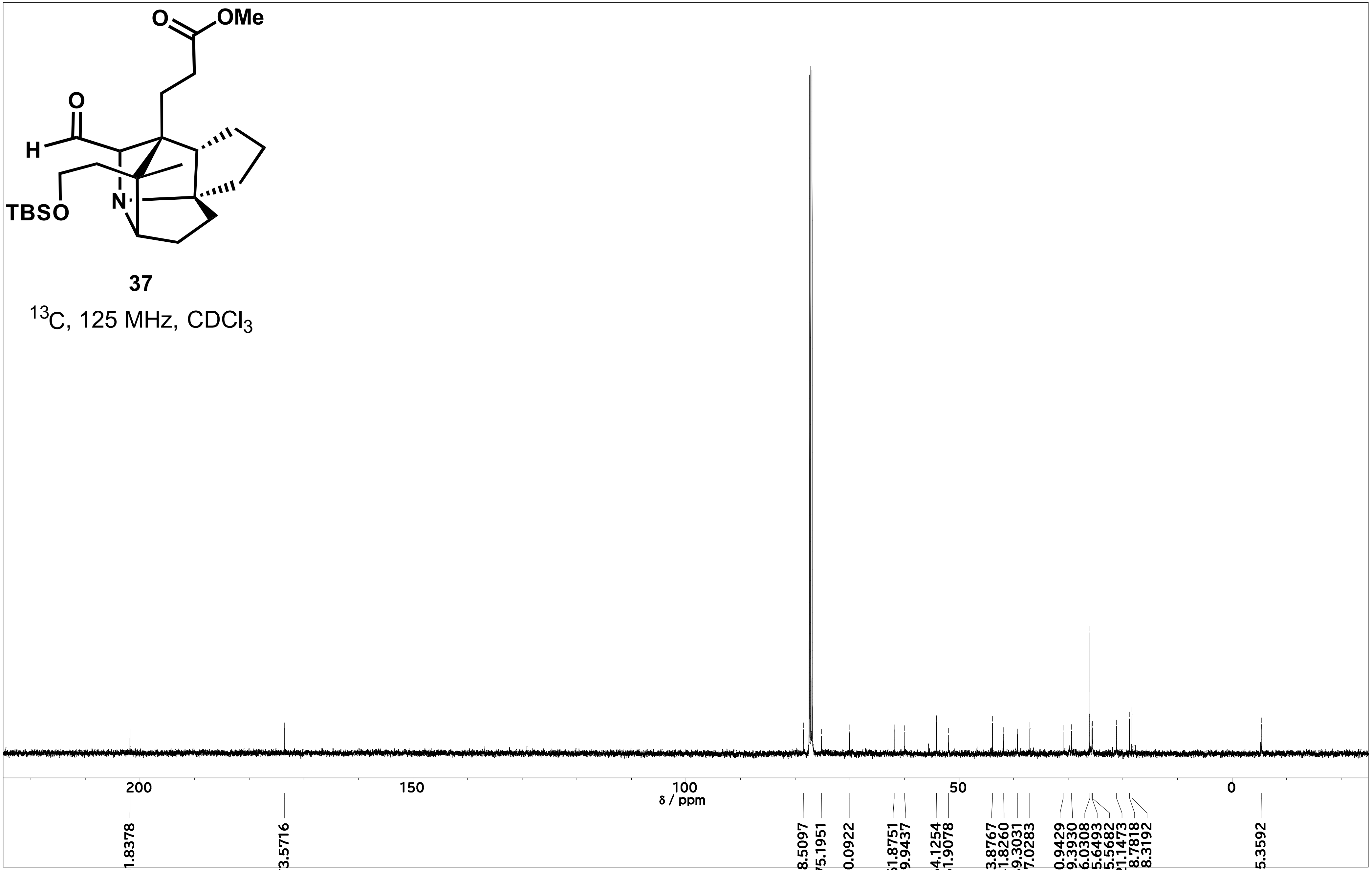
OMe
O
H
TBSO
N
37
^{13}C, 125 MHz, CDCl$_3$
200
150
100
50
0
δ / ppm
201.8378
173.5716
78.5097
75.1951
70.0922
61.8751
59.9437
54.1254
51.9078
43.8767
41.8260
39.3031
37.0283
30.9429
29.3930
26.0308
25.6493
25.5682
21.1473
18.7818
18.3192
-5.3592

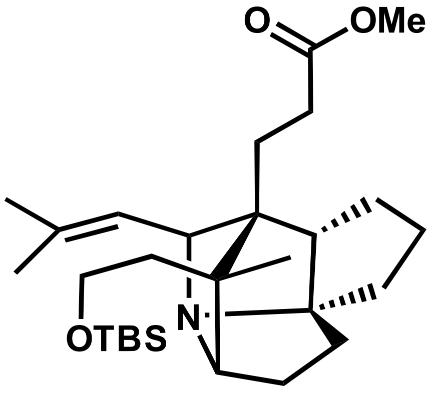

38

^{1}H, 500 MHz, C_6D_6

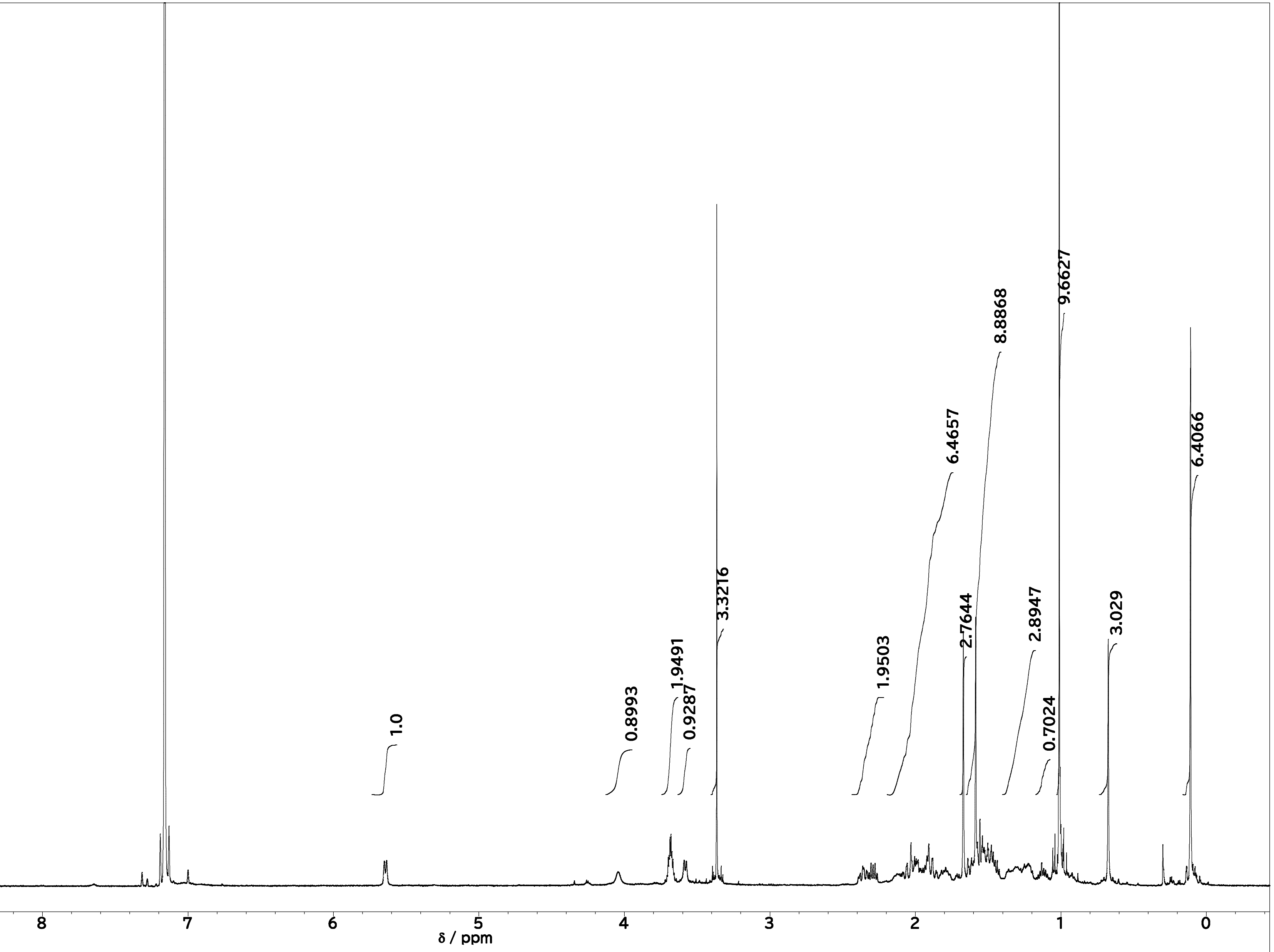

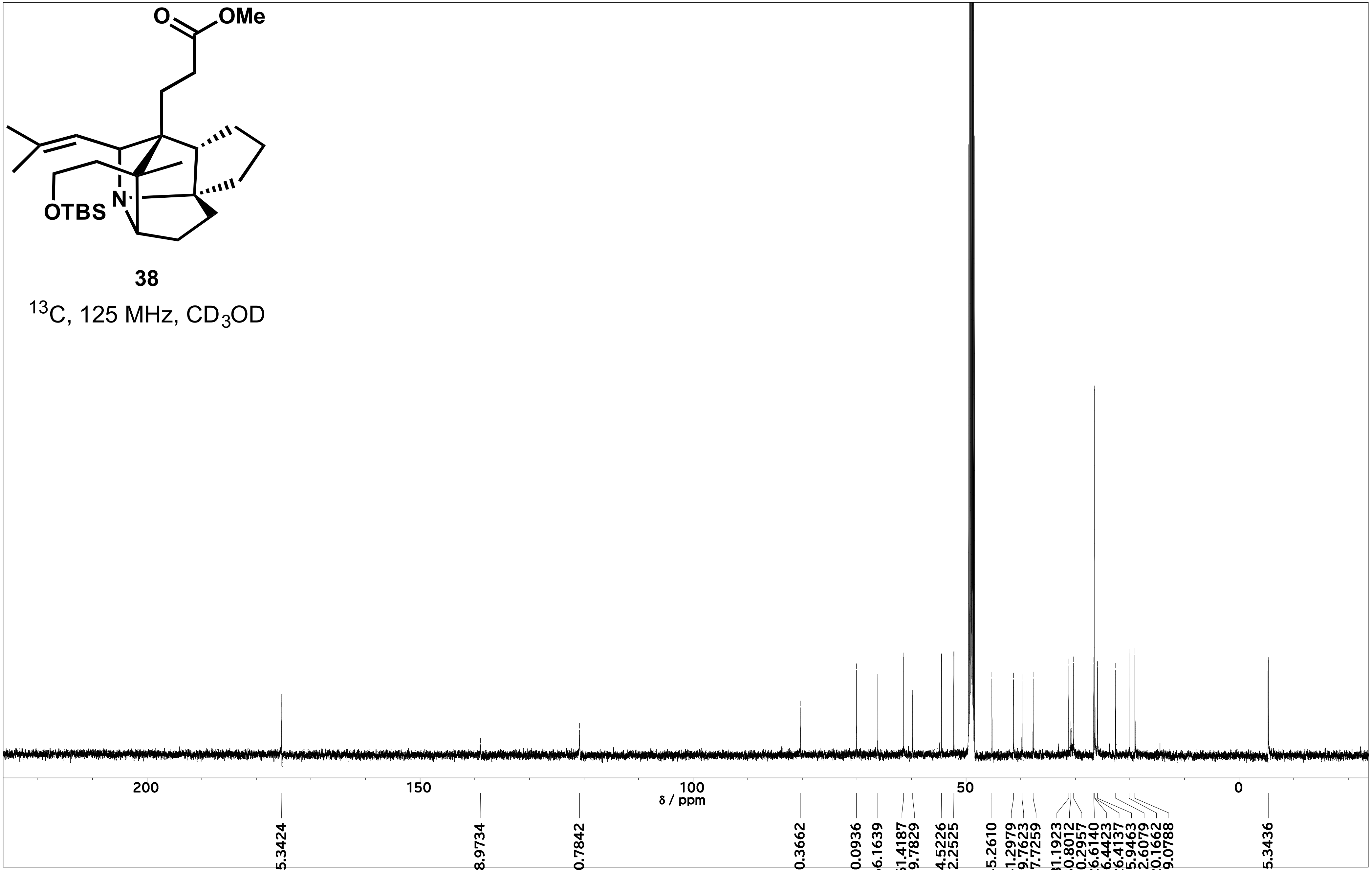

O
OMe
OTBS
N
38
^{13}C, 125 MHz, CD_3OD
200
150
100
50
0
δ / ppm
175.3424
138.9734
120.7842
80.3662
70.0936
66.1639
61.4187
59.7829
54.5226
52.2525
45.2610
41.2979
39.7623
37.7259
31.1923
30.8012
30.2957
26.6140
26.4423
26.4137
25.9463
22.6079
20.1662
19.0788
-5.3436

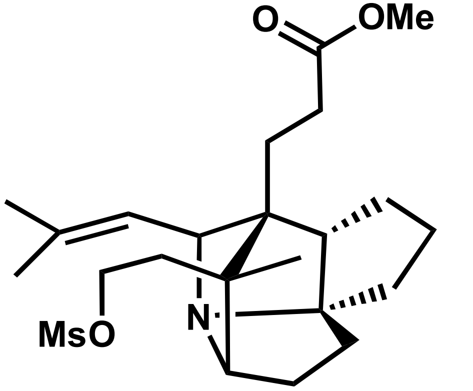

39

^{1}H, 500 MHz, $CDCl_3$

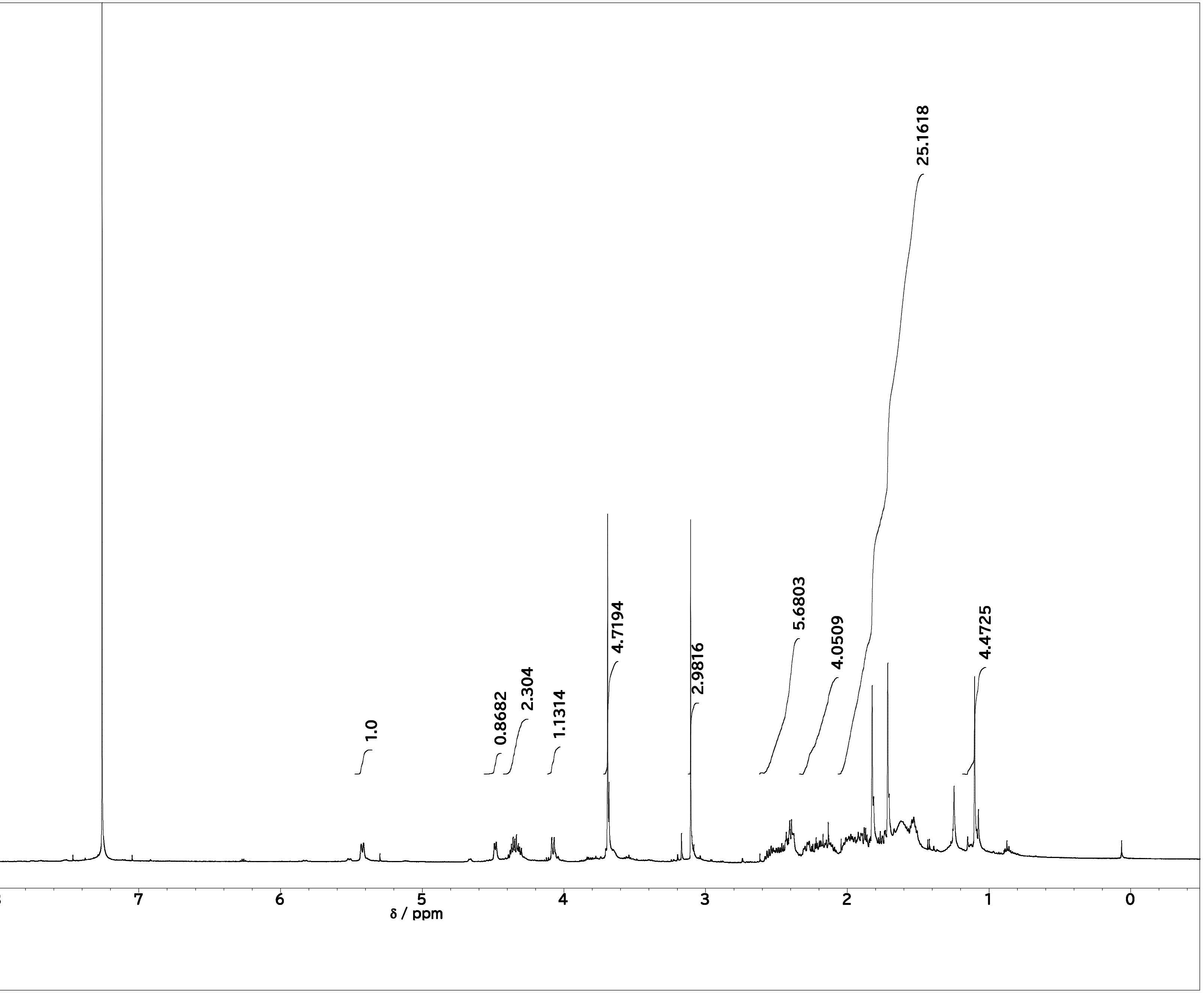

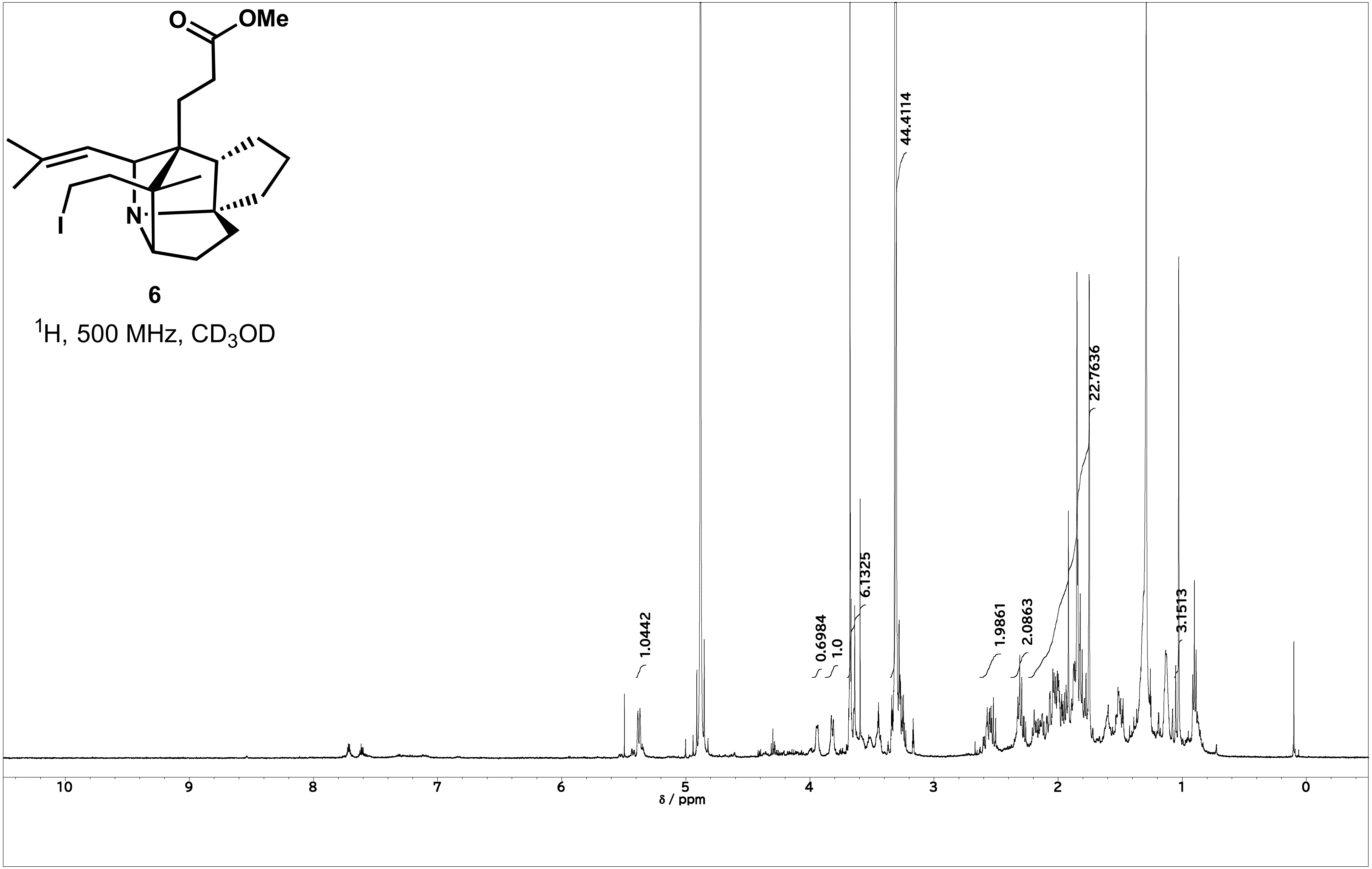
O
OMe
N
I
6
^{1}H, 500 MHz, CD_3OD
1.0442
0.6984
1.0
6.1325
44.4114
1.9861
2.0863
22.7636
3.1513
10
9
8
7
6
5
4
3
2
1
0
δ / ppm

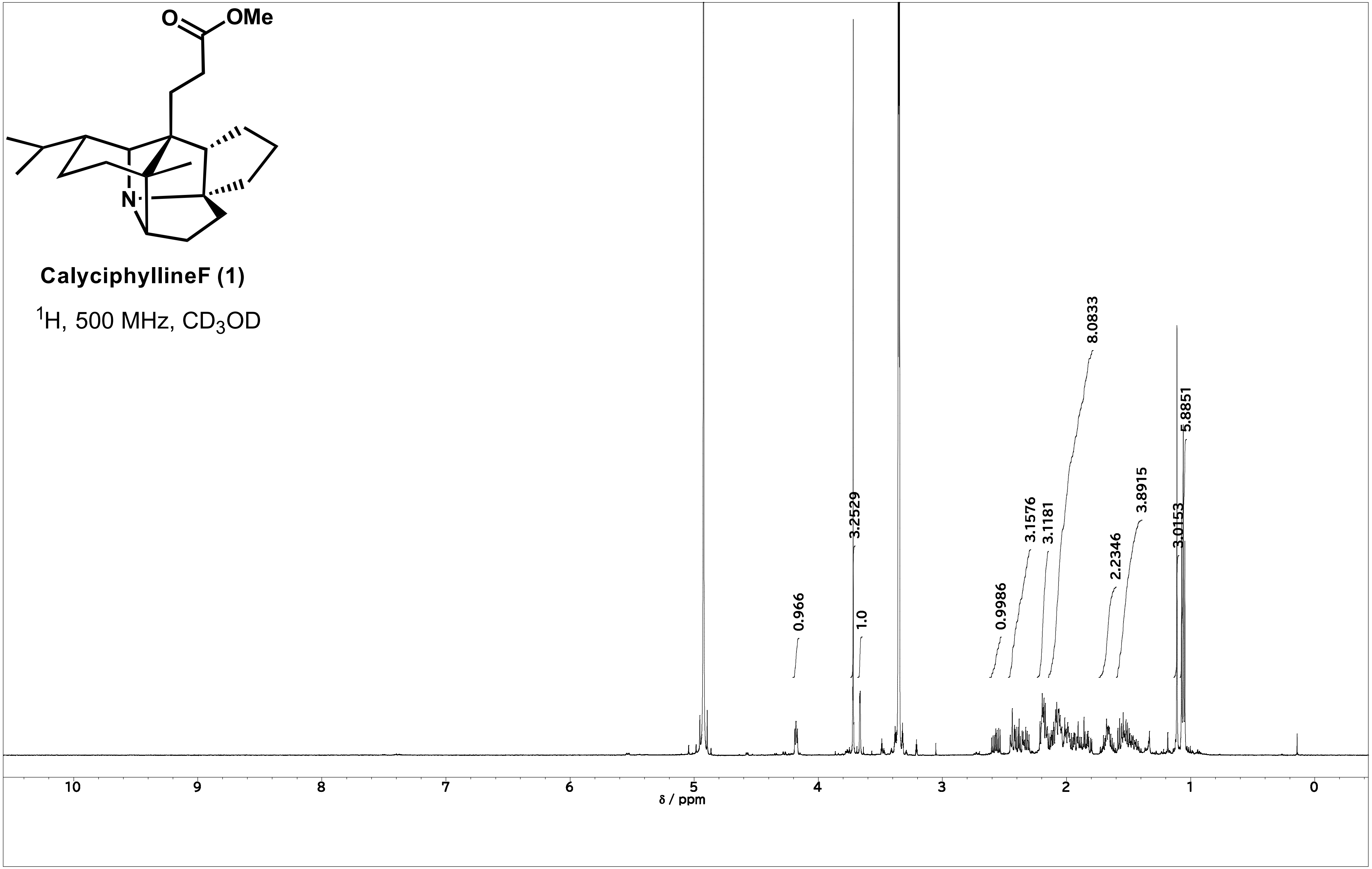

O
OMe
N
CalyciphyllineF (1)
1H, 500 MHz, CD3OD
0.966
3.2529
1.0
0.9986
3.1576
3.1181
8.0833
2.2346
3.8915
3.0153
5.8851
10
9
8
7
6
5
4
3
2
1
0
δ / ppm

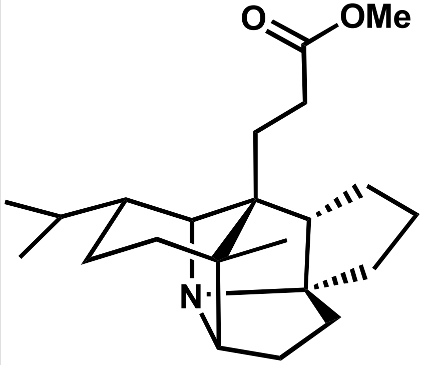

CalyciphyllineF (1)

^{13}C, 125 MHz, CD_3OD

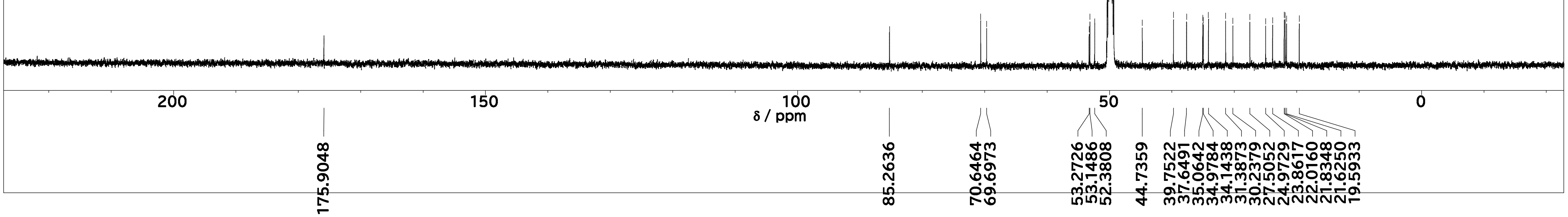

Supplement: Supplementary file 1 — Supporting Information [file ANIE-64-e202517671-s001.pdf]
